# Cation versus Radical: Studies on the C/O Regioselectivity in Electrophilic Tri-, Di- and Monofluoromethylations of β-Ketoesters

**DOI:** 10.1002/open.201200032

**Published:** 2012-10-11

**Authors:** Yu-Dong Yang, Xu Lu, Guokai Liu, Etsuko Tokunaga, Seiji Tsuzuki, Norio Shibata

**Affiliations:** a Department of Frontier Materials, Graduate School of Engineering, Nagoya Institute of TechnologyGokiso, Showa-ku, Nagoya 466-8555 (Japan) E-mail: nozshiba@nitech.ac.jp; b National Institute of Advanced Industrial Science and Technology (AIST)Tsukuba, Ibaraki 305-8568 (Japan) E-mail: s.tsuzuki@aist.go.jp

**Keywords:** C-alkylation, enolates, fluoromethylation, O-alkylation, regioselectivity

Efficient synthesis of fluorinated organic compounds, which plays an important role in the research of biological and medicinal chemistry, and material science, is now becoming one of the most dynamic aspects of modern organic chemistry.[1] Among several strategies for this purpose, late-stage fluoromethylation using easy-to-handle reagents under mild conditions is principally advantageous for the synthesis of complex molecules. Transferring a fluoromethyl group from the reagent to a target molecule is key for the reaction, and the reagents are classified according to their nucleophilic or electrophilic character.[2] Over the past two decades, electrophilic tri-, di- and monofluoromethylation have attracted considerable attention.[3–5] During our research program for the development of direct fluoromethylation reactions and the synthesis of biologically attractive organofluorine compounds,[6] we came across unique phenomena on C/O regioselectivity on the electrophilic tri- and monofluoromethylation reactions of β-ketoesters using fluorinated methylsulfoxinium salts **2 a** and **2 b**.3h, 5c Electrophilic trifluoromethylation of β-ketoesters **1** by **2 a** selectively occurs on the carbon centers of enolates, rather than on corresponding oxygen atoms,5c while monofluoromethylation by **2 b** takes place on the oxygen atoms completely regioselectively in the enolate alkylation.3h The curious results spurred us to investigate more closely the mechanistic aspect of the electrophilic fluoromethylation reactions of β-ketoesters. We herein disclose that different mechanisms are operating in the tri- and monofluoromethylation of β-ketoesters **1** from the view point of experimental results and computations. The C/O preference was found to be highly dependent on the number of fluorine atoms in the fluoromethyl group. Trifluoromethylation involves the formation of more cationic species represented by ^+^CF_3_ under the reaction conditions to provide complete C-alkylated products, while monofluoromethylation proceeds involving a radical-like species such as ^.^CFH_2_ to furnish completely O-alkylated products. Difluoromethylation of β-ketoesters **1** by difluoromethylsulfoxinium salts **2 c** was also investigated, and a mechanism joining the ^+^CF_2_H cation with the ^.^CF_2_H radical species is suggested (Scheme 1).

**Scheme 1:**
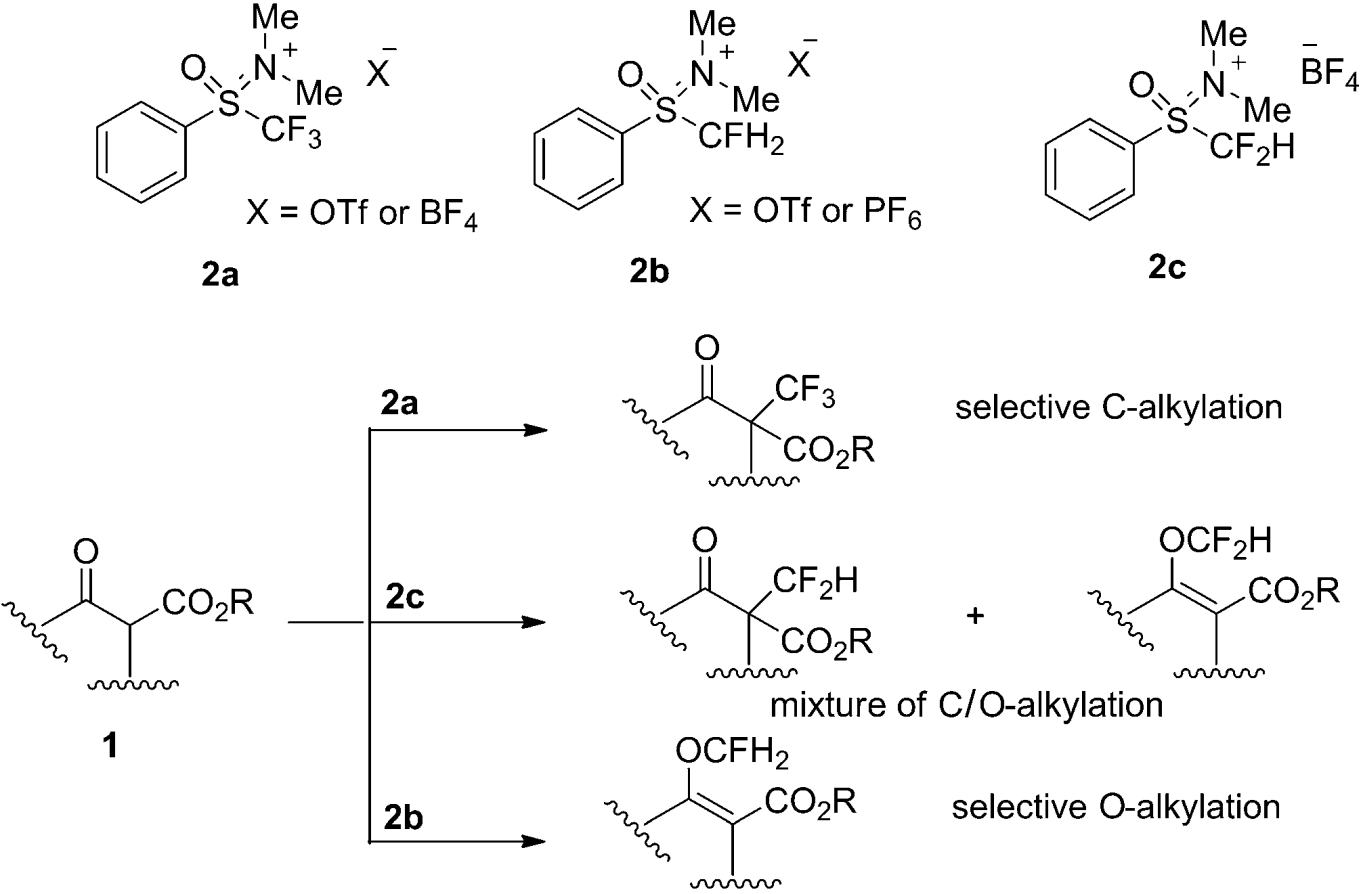
C/O selectivity of fluoromethylations of β-ketoesters.

Control of C and O regioselectivity in enolate alkylation is one of the oldest subjects in organic chemistry.3h, [7] The C/O-regioisomer ratio is sensitive to the extent of enolization of substrates that are highly dependent on the structure of carbonyl compounds and also the nature of alkylating reagents and reaction conditions, in particular the solvent and base. It has been shown that C-alkylation tends to be observed more frequently with softer electrophiles, while O-alkylation is preferred with harder electrophiles.[8, 9] However, the complete control of C/O regioselectivity is still a challenge, for example, in the O-regioselective methylation of β-ketoesters.3h, [9, 10] Matsuyama and co-workers carefully examined the methylation of methyl 1-indanone-2-carboxylate (**1 a**) using two types of methyl sulfonium salts A and B in the presence of K_2_CO_3_ in dichloromethane. Independent of the salts used, the C-methylation product was predominantly obtained. They also examined the same reaction using methyl sulfonium salts containing a chiral moiety to provide the C-methylation product with a low chiral induction. They concluded that the enolate ion of **1 a** attacks at the methyl carbon atom of the sulfonium salts through an ionic S_N_2 process after the formation of a S—O sulfurane intermediate (Scheme 2).[10] In this context, our findings of complete C selectivity in trifluoromethylation5c and O selectivity in monofluoromethylation3h are of great interest not only for the synthesis of fluorinated compounds but also for the mechanistic aspect of alkylations. The number of fluorine atoms should have an effect on C and O selectivities.

**Scheme 2:**
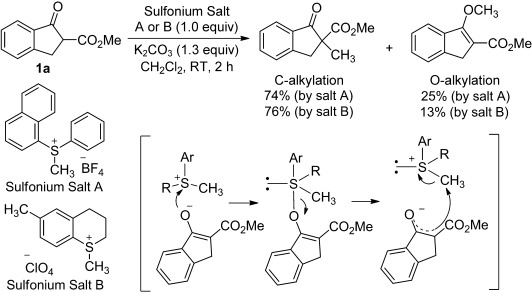
C/O selectivity of methylations of β-ketoester 1 a by methyl sulfonium salts A and B predominantly afford C-alkylated product.

Before initiating the computations, it is important to know the regioselectivity of difluoromethylation of β-ketoesters using **2 c**, which was not previously examined. Recently, Prakash and co-workers reported the synthesis of **2 c** and revealed that this reagent is effective for a broad spectrum of nucleophilic species;4i however, difluoromethylation of β-ketoesters by **2 c** was not examined. We began our investigation of difluoromethylation with **1 a** as a model substrate with difluoromethylating reagent **2 c** generated in situ under the conditions3h, 5c previously described for our fluoromethylations with **2 a** and **2 b** (Table 1). Different from mono- and trifluoromethylation, a mixture of C/O-alkylated compounds **3 a** and **4 a** was obtained in 43 % yield independent of the solvent used (**3 a**/**4 a**=53:47, Entries 1 and 2). By replacing P_1_-*t*Bu with other bases, such as TMG and DBU, lower yields but similar C/O regioselectivities were obtained (Entries 3 and 4). Weaker bases were ineffective for this transformation with **2 c**, and no desired product was obtained in the absence of a base (Entries 5–7). The nature and amount of base showed an obvious influence on yield but had little effect on the C/O regioselectivity. Only 12 % yield was obtained when a catalytic amount of base was used, and when the amount of base was increased, the reaction afforded better results with 47 % yield (Entries 8–10). However, increasing the amount of base to 2.5 equivalents could not further improve the yield at room temperature (Entry 11). This could be attributed to the instability of CF_2_H reagent **2 c** and partly to the decomposition in the exothermic reaction.4i C/O regioselectivity and yield increased slightly with a lower reaction temperature (Entry 12). The best result was obtained with a 69:31 C/O-alkylated mixture in 68 % yield in the presence of 2.5 equivalents of P_1_-*t*Bu at −78 °C (Entry 13).

**Table 1:** Optimization and regioselectivity for difluoromethylation of β-ketoester 1 a[a]

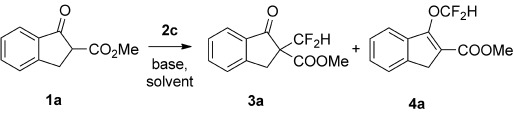						

Entry	2 c [equiv]	Base (equiv)[b]	Solvent	T [°C]	Yield [%][c]	Ratio 3 a/4 a[d]
1	2.0	P_1_-*t*Bu (1.5)	CH_3_CN	RT	43	53:47
2	2.0	P_1_-*t*Bu (1.5)	CH_2_Cl_2_	RT	43	53:47
3	2.0	TMG (1.5)	CH_3_CN	RT	34	47:53
4	2.0	DBU (1.5)	CH_3_CN	RT	21	53:47
5	2.0	Et_3_N (1.5)	CH_2_Cl_2_	RT	trace	–
6	2.0	Pyridine (1.5)	CH_2_Cl_2_	RT	trace	–
7	2.0	–	CH_2_Cl_2_	RT	0	–
8	2.0	P_1_-*t*Bu (0.1)	CH_2_Cl_2_	RT	12	58:42
9	2.0	P_1_-*t*Bu (1.05)	CH_2_Cl_2_	RT	30	50:50
10	3.0	P_1_-*t*Bu (1.5)	CH_2_Cl_2_	RT	47	55:45
11	3.0	P_1_-*t*Bu (2.5)	CH_2_Cl_2_	RT	47	55:45
12	3.0	P_1_-*t*Bu (1.5)	CH_2_Cl_2_	−78	52	69:31
13	3.0	P_1_-*t*Bu (2.5)	CH_2_Cl_2_	−78	68	69:31

[a] *Reagents and conditions:* A solution of **1 a** and base, which had been stirred in solvent for 15 min, was added to in situ generated **2 c** in CH_2_Cl_2_. The mixture was stirred at above given temperature for a further 2–3 h.

[b] P_1_-*t*Bu=*tert*-butylimino-tris(dimethylamino)phosphorane, TMG=tetramethylguanidine, DBU=1,8-diazabicyclo[5.4.0]undec-7-ene.

[c] Based on **1 a** and determined by ^19^F NMR using PhCF_3_ as the internal standard.

[d] Determined by ^19^F NMR of the crude products.

The scope of the difluoromethylation of β-ketoesters **1** with **2 c** was next investigated under the optimized condition. As shown in Table 2, C/O regioselectivity was almost independent of substrate **1**. When a bulkier ester moiety was introduced, the yield decreased but similar C/O selectivities were observed (Entries 1–3). The substituents on the aromatic moiety did not affect yield and regioselectivity significantly, and both electron-deficient and electron-rich substituents afforded similar yields with C/O selectivity (Entries 4–7).

**Table 2:** Scope of difluoromethylation of β-ketoesters 1[a]

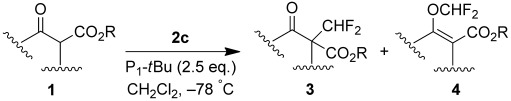					

Entry	β-Ketoester		Yield [%][b]	Ratio 3/4[c]	
1	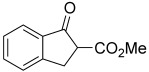	**1 a**	64	**3 a**/**4 a**	69:31
2	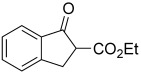	**1 b**	53	**3 b**/**4 b**	64:36
3	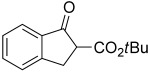	**1 c**	43	**3 c**/**4 c**	62:38
4	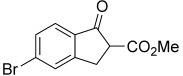	**1 d**	61	**3 d**/**4 d**	65:35
5	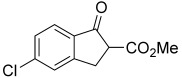	**1 e**	66	**3 e**/**4 e**	60:40
6	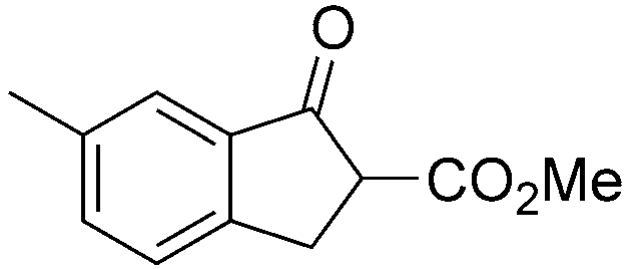	**1 f**	62	**3 f**/**4 f**	69:31
7	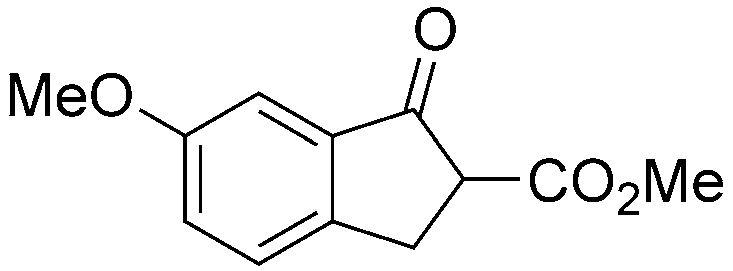	**1 g**	61	**3 g**/**4 g**	62:38

[a] *Reagents and conditions:* A solution of **1** and base, which had been stirred in CH_2_Cl_2_ for 15 min, was added to in situ generated **2 c** in CH_2_Cl_2_. The mixture was stirred at −78 °C for a further 3 h.

[b] Isolated yield.

[c] Determined by ^19^F NMR of the crude products.

These difluoromethylation experiments and our previous results for tri- and monofluoromethylations clearly reveal that C/O regioselectivity of the fluoromethylation of β-ketoesters **1** is highly dependent on the number of fluorine atoms on the fluoromethyl group, and is almost independent of the substrate structure of **1**, the solvent and the base used. Namely, C-alkylation tends to be observed with an increase in fluorine atoms, while O-alkylation is observed with a decrease in fluorine atoms in the fluoromethyl group. We hypothesize that C/O regioselectivity could be explained by the radical versus cationic species of CF_3_, CF_2_H and CFH_2_. The generation of a cation or radical species should be highly dependent on the number of fluorine atoms in the fluoromethyl group. The reaction process by electrophilic trifluoromethylation reagents is always a matter of debate, and there is no clear evidence to demonstrate that a cationic “^+^CF_3_” species is involved during the transition step.5a, [11] Umemoto and co-workers described that the reaction pathway can change from involving a CF_3_ radical to a CF_3_ cation depending on the nature of the nucleophile.[12] This hypothesis was later discussed by Magnier et al., who suggested a single electron-transfer (SET) pathway in their trifluoromethylation reaction through trapping experiments with a radical probe, at least in the case of nucleophiles such as enol silyl ethers.[11] To confirm our principal argument involving cationic versus radical processes, we examined tri-, di- and monofluoromethylations of **1 c** with **2 a–c** under optimized conditions in the presence of nitrobenzene, which is known for its ability to inhibit a radical pathway. However, the results were essentially the same as the results without nitrobenzene. We assume that these results do not rule out a radical pathway, because the entire process occurs in the solvent cage independent of the cationic or radical process, and so cannot be inhibited by a radical scavenger. Therefore, molecular orbital calculations were carried out for studying the reaction of β-ketoester anion **5** with cation or radical species of CF_3_, CF_2_H, or CFH_2_, providing C-alkylated or O-alkylated products (Scheme 3).[13]

**Scheme 3:**
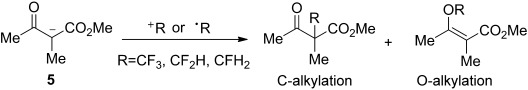
Model for computations.

The relative energies of four rotamers of anion **5** were optimized, and **5 a** was found to be the most stable (Figure 1 A). The atomic charge distributions of **5 a**, fluoromethyl cations (^+^CF_3_, ^+^CF_2_H, ^+^CFH_2_) and fluoromethyl radicals (^.^CF_3_, ^.^CF_2_H, ^.^CFH_2_) were next calculated (Figure 1 B). The negative charge of **5 a** was mainly located on the oxygen atoms of carbonyl groups and on the carbon atom between carbonyl groups. The calculated charges on the carbonyl oxygen atoms were −0.60 e and −0.61 e and that on the carbon atom was −0.51 e. The positive charge of the fluoromethyl cations ^+^CF_3_, ^+^CF_2_H, and ^+^CFH_2_ was mainly located on the carbon atoms (0.95 e, 0.72 e and 0.58 e, respectively). Next, the geometries of **5 a** complexed with fluoromethyl cations ^+^CF_3_, ^+^CF_2_H, and ^+^CFH_2_ were optimized (Figure 2). In the initial geometries for the trifluoromethylation, the ^+^CF_3_ cation was located close to the carbon atom between two carbonyl groups (**6 a**) or one of the oxygen atoms of carbonyl groups (**6 b** and **6 c**, Figure 2 A). The C- or O-alkylated products **7 a–c**, spontaneously produced by the geometry optimizations of complexes **6 a–c**, show that no potential energy barrier for the formation of C—C and C—O bonds exists during cationic trifluoromethylation (Figure 2 B).[14] The calculations of relative energies in Figure 2 B show that the C—CF_3_ products are significantly more stable than the O—CF_3_ products. O-alkylated **7 b** is 14.30 kcal mol^−1^ and O-alkylated **7 c** is 30.80 kcal mol^−1^ less stable than C-alkylated **7 a**. The geometries and relative energies of the alkylated products obtained by the geometry optimizations of **5** complexed with ^+^CF_2_H and ^+^CFH_2_ are shown in Figure 2 C and 2 D.[14] C-alkylated **8 a** and **9 a** were significantly more stable (14.83 to 33.48 kcal mol^−1^) than O-alkylated **8 b**, **8 c**, **9 b** and **9 c**, as in the case of **7**. The larger stability of C-alkylated **7 a**, **8 a** and **9 a** suggests that the reactions of **5** with cations ^+^CF_3_, ^+^CF_2_H, and ^+^CFH_2_ prefer to produce C-alkylated products independent of the number of fluorine molecules. The complete C regioselectivity for the trifluoromethylation can be explained by the cationic process. This hypothesis is also supported by calculations based on radical species in which a radical process would be ruled out for the trifluoromethylation (see below).

**Figure 1:**
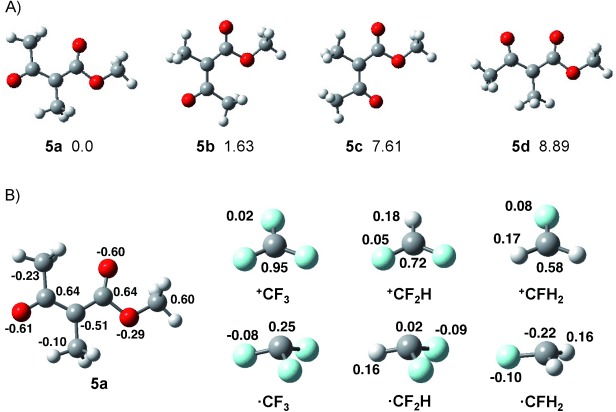
A) Relative energies of four rotamers of 5 at the MP2/6-311G** level. Energy in kcal mol^−1^. B) Atomic charge distributions of 5 a, ^+^CF_3_, ^+^CF_2_H, ^+^CFH_2_, ^.^CF_3_, ^.^CF_2_H, and ^.^CFH_2_. Atomic charges of methyl groups are summed.

**Figure 2:**
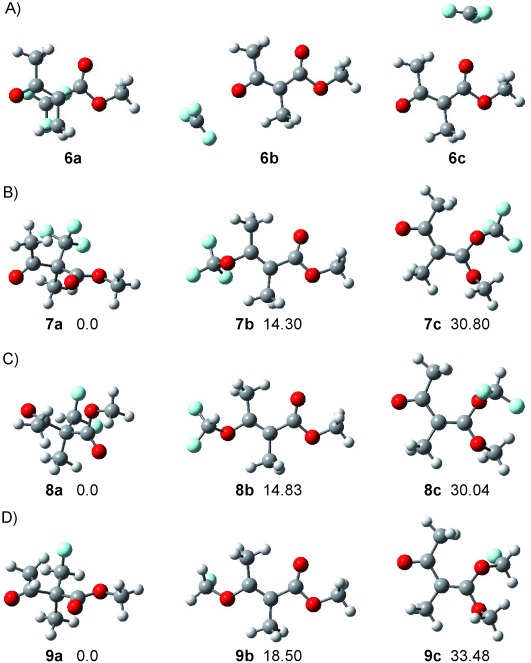
A) The initial geometries for trifluoromethylation before geometry optimizations; ^+^CF_3_ cation is located close to the carbon atom between two carbonyl groups (6 a), or close to one of the oxygen atoms of the carbonyl groups (6 b and 6 c). B) The optimized geometries and relative energies of C—CF_3_ product 7 a and O—CF_3_ products 7 b, c at the MP2/6-311G** level. Energy in kcal mol^−1^. C) The optimized geometries and relative energies of C—CF_2_H product 8 a and O—CF_2_H products 8 b, c. D) The optimized geometries and relative energies of C—CFH_2_ product 9 a and O—CFH_2_ products 9 b, c.

The geometries of anion **5** complexed with fluoromethyl radicals ^.^CF_3_, ^.^CF_2_H, ^.^CFH_2_ were investigated next (Figure 3). They were optimized starting from three initial geometries similar to the case of **5** and ^+^CF_3_, as shown in Figure 2 A.[15] The optimized geometries of complexes **10**–**12** and the stabilization energies (*E*_form_) are shown in Figure 3 A–C.[15] It is interesting to note that the stability of the complexes of **5** with fluoromethyl radicals ^.^CF_3_, ^.^CF_2_H, and ^.^CFH_2_ is highly dependent on the number of fluorine molecules in the fluoromethyl group. The interaction of **5** with the ^.^CF_3_ radical is very weak (<1 kcal mol^−1^, Figure 3 A), which could exclude a radical mechanism for trifluoromethylation. On the other hand, the corresponding interactions of **5** with ^.^CF_2_H and ^.^CFH_2_ radicals are much stronger than that of the ^.^CF_3_ radical (Figure 3 B and 3 C). The *E*_form_ of the most stable complexes **11 b** for ^.^CF_2_H and **12 a** for ^.^CFH_2_ are −8.46 and −5.66 kcal mol^−1^, respectively. Despite the initial geometries before calculations where the ^.^CF_2_H and ^.^CFH_2_ radicals are located near the carbon atom between the two carbonyl groups of **5**, the ^.^CF_2_H and ^.^CFH_2_ radicals were found near one of the oxygen atoms in the optimized geometries **11 a** and **12 a**. That is, the ^.^CF_2_H and ^.^CFH_2_ radicals prefer to locate close to one of the oxygen atoms of the carbonyl groups of **5** which produce O-alkylated products. The complete O regioselectivity found in the monofluoromethylation can be explained by the radical-like mechanism involving the SET process.

**Figure 3:**
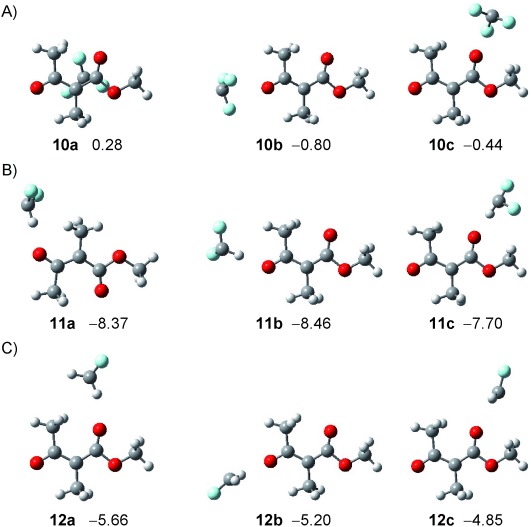
A) Three optimized geometries of 5 with ^.^CF_3_ and their stabilization energies at the MP2/6-311G** level. Energy in kcal mol^−1^. B) Three optimized geometries of 5 with ^.^CF_2_H and their stabilization energies. C) Three optimized geometries of 5 with ^.^CFH_2_ and their stabilization energies.

For the difluoromethylation of β-ketoesters with **2 c**, both cationic and radical processes are suggested based on the above calculations (Figure 2 C and 3 B). Prakash and co-workers elucidated through isotope-labeling experiments that the difluoromethylation of nucleophiles, including alcohols by **2 c**, proceeds in an electrophilic alkylation manner (^+^CF_2_H) instead of the commonly adopted difluorocarbene pathway.4i In our experimental results, the existence of a mixture of O- and C-alkylated products in difluoromethylation could be explained by the mechanism joining cation ^+^CF_2_H with radical ^.^CF_2_H species. The balance of ^+^CF_2_H/^.^CF_2_H species could be influenced slightly by the reaction temperature (Entries 10–13, Table 1), an observation which is not found for tri- and monofluoromethylations of β-ketoesters.3h, 5c

Based on the computations, plausible schematic reaction mechanisms for monofluoromethylation and trifluoromethylation are shown in Figure 4. Similar to the mechanism of methylation shown in Scheme 2 by Matsuyama and co-workers,[10] monofluoromethylation would proceed through an attack of the enolate oxygen to the sulfur center of **2 b** to afford a sulfurane-type intermediate **TS-I**, which generates O and CFH_2_ radicals with dimethylamino phenyl sulfinamide (Figure 4 A). On the other hand, due to an electron deficient character of the CF_3_ group, the enolate might attack directly at the more cationic trifluoromethyl carbon center of **2 a** to give the C-alkylated product through an ionic S_N_2 pathway (Figure 4 B).

**Figure 4:**
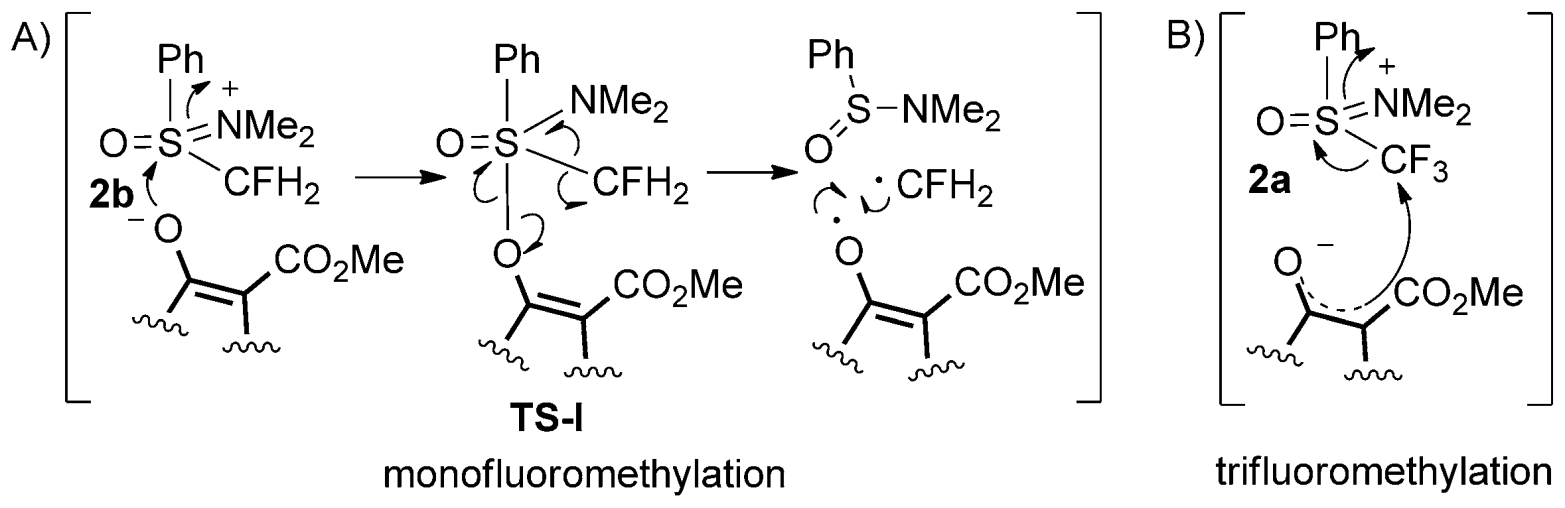
Proposed reaction mechanisms for A) monofluoromethylation and B) trifluoromethylation.

In conclusion, the C/O regioselectivity in fluoromethylations of β-ketoesters **1** with fluorinated methylsulfoxinium salts **2 a–c** was discussed based on experimental results and computations. The experimental result for the electrophilic difluoromethylation of β-ketoesters **1** with **2 c** giving a mixture of C and O isomers is very different from the results of tri- and monofluoromethylations of β-ketoesters by **2 a** or **2 b**. The computational studies disclosed that the C/O regioselectivity in fluoromethylations of β-ketoesters should be attributed to the character of mono-, di- and trifluoromethyl cations or radicals. Trifluoromethylation involves the formation of a more cationic species represented by ^+^CF_3_ to provide C-alkylated products, while monofluoromethylation possibly proceeds involving a more radical-like species such as ^.^CFH_2_ to give O-alkylated species. Difluoromethylation could involve both cationic and radical species to afford a mixture of C and O isomers. These mechanistic aspects of electrophilic fluoromethylations based on the preference of carbon or oxygen could provide another solution for the long-standing synthetic subject of C and O regioselectivity in enolate alkylation. More detailed calculations including solvent/base effects, structures of fluoromethylating reagents, and the Pearson acid base concept using a variety of substrates will be necessary for getting a final conclusion, and we are currently working in this direction.

## Experimental Section

**Computational methods**: The Gaussian 03 program[16] was used for the ab initio molecular orbital calculations. Electron correlation was accounted for by the second-order M*ϕ*ller–Plesset perturbation (MP2) method.[17, 18] The 6-311G** basis set was used for the calculations. The stabilization energy by the formation of a complex from isolated species (*E*_form_) was calculated as the sum of the interaction energy (*E*_int_) and the deformation energy (*E*_def_). *E*_def_ is the sum of the increase of the energies of monomers by the deformation associated with the formation of the complex. *E*_int_ was calculated by the supermolecule method. The basis set superposition error (BSSE)[19] was corrected for the interaction energy calculations using the counterpoise method.[20] The atomic charges were obtained by electrostatic potential fitting using the Merz–Singh–Kollman scheme[21, 22] from the MP2/6-311G** level wave functions of the isolated molecules. Further details on the molecular calculations can be found in the Supporting Information.

## Supplementary material

Detailed facts of importance to specialist readers are published as ”Supporting Information”. Such documents are peer-reviewed, but not copy-edited or typeset. They are made available as submitted by the authors.
