# Supplementary material for: Cation versus Radical: Studies on the C/O Regioselectivity in Electrophilic Tri-, Di- and Monofluoromethylations of β-Ketoesters
Source: ChemistryOpen. 2012 Oct 11;1(5):221–6. doi: 10.1002/open.201200032 (PMC3922593; doi:10.1002/open.201200032)
Supplement: Supplementary file 1 [file open0001-0221-SD1.pdf]

ChemistryOPEN

Supporting Information

© 2012 The Authors. Published by Wiley-VCH Verlag GmbH & Co. KGaA, Weinheim

Cation versus Radical: Studies on the C/O Regioselectivity in Electrophilic Tri-, Di- and Monofluoromethylations of β-Ketoesters

Yu-Dong Yang,[a] Xu Lu,[a] Guokai Liu,[a] Etsuko Tokunaga,[a] Seiji Tsuzuki,*[b] and Norio Shibata*[a]

open_201200032_sm_miscellaneous_information.pdf

Experimental

General experimental details

All reactions were performed in oven-dried glassware under a positive pressure of nitrogen. Solvents were transferred via syringe and were introduced into the reaction vessels through a rubber septum. All of the reactions were monitored by thin-layer chromatography (TLC) carried out on 0.25 mm Merck silica gel (60-F254). The TLC plates were visualized with UV light and 7% phosphomolybdic acid or $KMnO_4$ in water/heat. Column chromatography was carried out on a column packed with silica gel 60N spherical neutral size 63-210 μm. The ^{1}H NMR (300 MHz) and ^{19}F NMR (282 MHz) spectra for solution in $CDCl_3$, were recorded on a Varian Mercury 300. ^{13}C NMR (150.9 MHz) spectra were recorded on a BRUKER 600 UltraShield^{TR}. Chemical shifts (δ) are expressed in ppm downfield from internal TMS or $CFCl_3$. Mass spectra were recorded on a SHIMADZU GCMS-QP5050A (EI-MS) and SHIMAZU LCMS-2010EV (ESI-MS and APCI-MS). Infrared spectra were recorded on a JASCO FT/IR-200 or a JASCO FT/IR-4100 spectrometer. All the substrates (**1a**–**1g**) were prepared according to the literature procedure.[1]

The Gaussian 03 program[2] was used for the ab initio molecular orbital calculations. Electron correlation was accounted for by the second-order Mϕller-Plesset perturbation (MP2) method.[3,4] The 6-311G** basis set was used for the calculations. The stabilization energy by the formation of the complex from isolated species (E_{form}) was calculated as the sum of the interaction energy (E_{int}) and the deformation energy (E_{def}). The E_{def} is the sum of the increase of the energies of monomers by the deformation associated with the formation of the complex. The E_{int} was calculated by the supermolecule method. The basis set superposition error (BSSE)[5] was corrected for the interaction energy calculations using the counterpoise method.[6] The atomic charges were obtained by electrostatic potential fitting using Merz-Singh-Kollman scheme[7,8] from the MP2/6-311G** level wave functions of the isolated molecules.

Computations:

Relative energies for rotamers of $MeCOCMeCOOMe^-$

The four stable rotamers were obtained for the $MeCOCMeCOOMe^-$ anion (**5**) by geometry optimizations. The optimized geometries and relative energies for the rotamers are shown in Figure 1S. The rotamer **5a** was the most stable. The geometry of **5a** was used for preparing initial geometries for the geometry optimizations of the $MeCOCMeCOOMe^-$ complexes with cations and radicals.

Charge distributions

The atomic charges calculated for the $MeCOCMeCOOMe^-$ anion, $^+CF_3$, $^+CHF_2$, $^+CH_2F$ cations and $\cdot CF_3$, $\cdot CHF_2$, $\cdot CH_2F$ radicals are summarized in Figure 2S. The negative charge of the

$MeCOCMeCOOMe^-$ anion mainly locates on the oxygen atoms of carbonyl groups and the carbon atom between the carbonyl groups. The calculated charges on the carbonyl oxygen atoms are -0.60 e and -0.61 e and that on the carbon atom is -0.51 e. The positive charge of the $^+CF_3$, $^+CHF_2$, and $^+CH_2F$ cations mainly locates on the carbon atoms (0.58 e to 0.95 e). The positive charges on the hydrogen atoms of the $^+CHF_2$, $^+CH_2F$ cations are smaller (0.17 e to 0.18 e). The hydrogen atoms of the $\cdot CHF_2$, $\cdot CH_2F$ radicals have positive charge (0.16 e).

Geometry optimizations of $MeCOCMeCOOMe^-$ complexes with $^+CF_3$, $^+CHF_2$, $^+CH_2F$ cations

The geometries of the $MeCOCMeCOOMe^-$ complex with the $^+CF_3$ cation were optimized from three initial geometries shown in Figure 3S. The $^+CF_3$ cation locates near the carbon atom between the two carbonyl groups in **6a**, while it locates near one of the oxygen atoms of the two carbonyl groups in **6b** and **6c**. The C- or O-alkylated products (**7**) were obtained by the geometry optimizations, which show that there exists no potential energy barrier for the formation of the C-C and C-O bonds. The optimized geometries and relative energies of the products are shown in Figure 4S. The geometries of the $MeCOCMeCOOMe^-$ complexes with $^+CHF_2$ and $^+CH_2F$ cations were optimized from initial geometries similar to **6**. The C- or O-alkylated products (**8** and **9**) were obtained by the geometry optimizations as in the case of **7**. The optimized geometries and relative energies of the products are shown in Figures 5S and 6S.

The calculations show that the C-alkylated products are significantly more stable than the O-alkylated products. The O-alkylated products with the $^+CF_3$ cation (**7b** and **7c**) are 14.30 and 30.80 kcal/mol less stable than the C-alkylated product (**7a**), respectively. The O-alkylated products with the $^+CHF_2$ (**8b** and **8c**) are 14.83 and 30.04 kcal/mol less stable than the C-alkylated products (**8a**). The O-alkylated products with the $^+CH_2F$ (**9b** and **9c**) are 18.50 and 33.48 kcal/mol less stable than the C-alkylated products (**9a**). The larger stability of the C-alkylated products suggests that the reaction of the $MeCOCMeCOOMe^-$ anion with the cations prefers to produce C-alkylated products. The C-regioselectivity of the trifluoromethylation will be explained if the $^+CF_3$ cation is the reactive species in the trifluoromethylation reaction.

Geometry optimizations of $MeCOCMeCOOMe^-$ complexes with $\cdot CF_3$, $\cdot CHF_2$ and $\cdot CH_2F$ radicals

The geometries of the $MeCOCMeCOOMe^-$ complex with $\cdot CF_3$ radical (**10**) were optimized from three initial geometries similar to those of the $MeCOCMeCOOMe^-$ complex with the $^+CF_3$ cation. The optimized geometries and the stabilization energies (E_{form}) of the complex are shown in Figure 7S. The interaction between the $MeCOCMeCOOMe^-$ anion and the $\cdot CF_3$ radical is very weak.

The $\cdot CF_3$ radical locates close to the carbon atom between the two carbonyl groups of $MeCOCMeCOOMe^-$ anion in **10a**. The **10a** has positive E_{form} (0.28 kcal/mol), which shows that **10a** is less stable than the isolated species, although the geometry of **10a** corresponds to a local minimum on the potential energy surface. The $\cdot CF_3$ radical locates close to one of the oxygen atoms of the two carbonyl groups in **10b** and **10c**. The E_{form} for **10b** and **10c** are very small (-0.80 kcal/mol and -0.44 kcal/mol, respectively).

The geometries of the $MeCOCMeCOOMe^-$ complexes with $\cdot CHF_2$ and $\cdot CH_2F$ radicals (**11** and **12**) were optimized from similar initial geometries. The optimized geometries and the stabilization energies (E_{form}) of the complexes are shown in Figures 8S and 9S. Although the geometry optimizations of **11a** and **12a** were started from the initial geometries where the $\cdot CHF_2$ and $\cdot CH_2F$ radicals locate near the carbon atom between the two carbonyl groups of the $MeCOCMeCOOMe^-$ anion, the radicals locate near one of the oxygen atoms of the carbonyl groups in the optimized geometries (**11a** and **12a**). The radicals locate close to one of the oxygen atoms of carbonyl groups in other optimized geometries in Figures 8S and 9S. These geometries were obtained from the initial geometries where the radicals locate near one of the oxygen atoms of the carbonyl groups. The E_{form} calculated for the most stable $MeCOCMeCOOMe^-$ complexes with $\cdot CHF_2$ and $\cdot CH_2F$ radicals (**11** and **12**) are -8.46 and -5.66 kcal/mol. The hydrogen atoms of the $\cdot CHF_2$ and $\cdot CH_2F$ radicals have contact with the oxygen atom of the carbonyl groups.

The calculations show that the $\cdot CHF_2$ and $\cdot CH_2F$ radicals prefer to have contact with one of the oxygen atoms of carbonyl groups of the $MeCOCMeCOOMe^-$ anion in the complexes due to the substantial attraction between the radicals and the oxygen atoms. The stable structures of the complexes suggest that the $\cdot CHF_2$ and $\cdot CH_2F$ radicals prefer to produce O-alkylated products. The O-regioselectivity of the di- and mono-fluoromethylation will be explained if the $\cdot CHF_2$ and $\cdot CH_2F$ radicals are the reactive species in the di- and mono-fluoromethylation reactions.

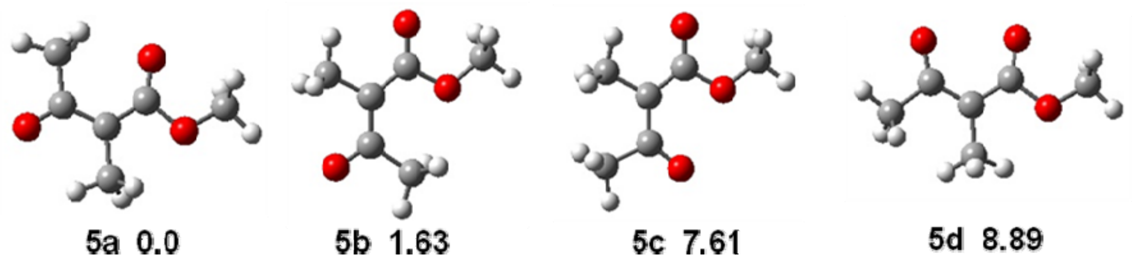

Figure 1S. Optimized geometries and calculated relative energies for four rotamers of $MeCOCMeCOOMe^-$ anion (**5**) at the MP2/6-311G** level. Energy in kcal/mol.

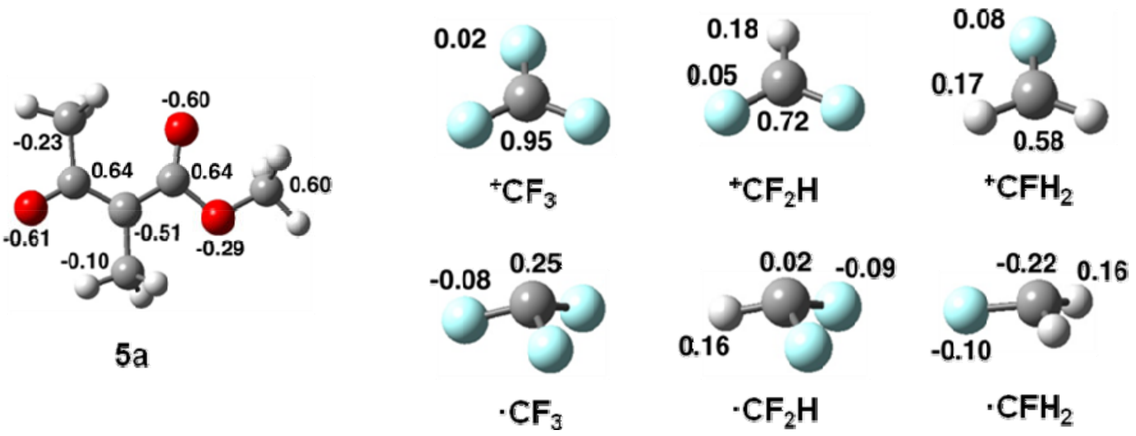

Figure 2S. The atomic charges for the MeCOCMeCOOMe$^-$ anion (**5**), $^+CF_3$, $^+CHF_2$, $^+CH_2F$ cations and $\cdot CF_3$, $\cdot CHF_2$, $\cdot CH_2F$ radicals obtained by electrostatic potential fitting from the MP2/6-311G** level wave functions. Atomic charges with methyl hydrogens were summed into the carbon atoms.

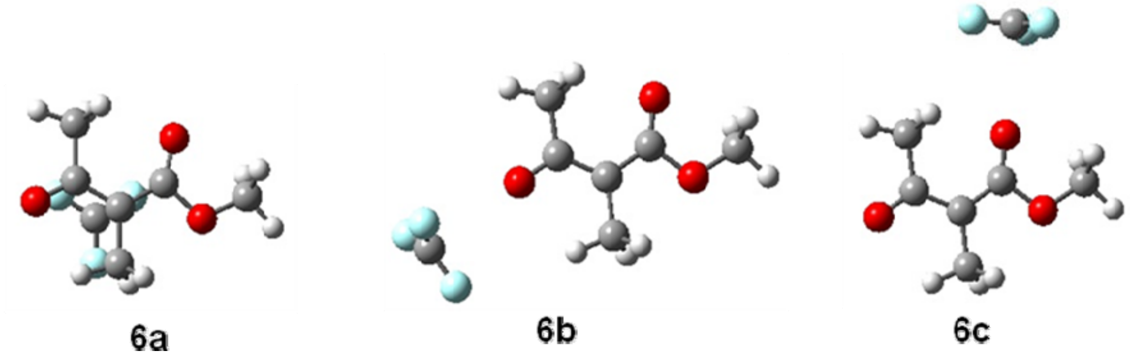

Figure 3S. Initial geometries for geometry optimizations of the MeCOCMeCOOMe$^-$ complex with CF_3^+.

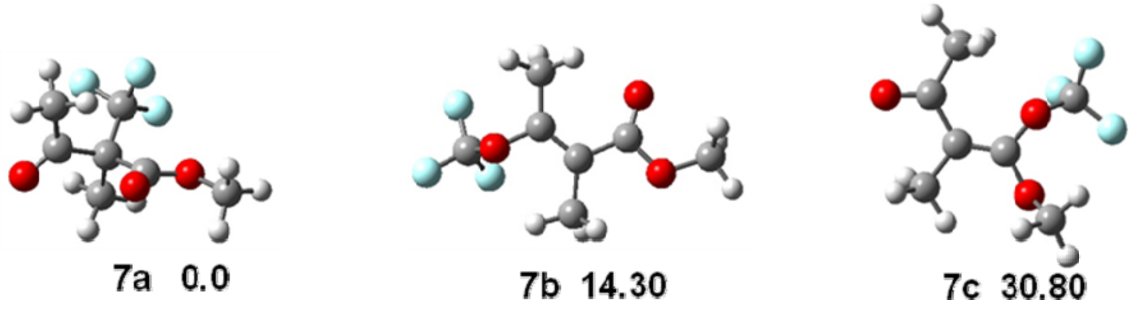

Figure 4S. Optimized geometries and relative energies calculated for the C- or O-alkylated

products of MeCOCMeCOOMe$^-$ with $^+CF_3$ at the MP2/6-311G** level. Energy in kcal/mol.

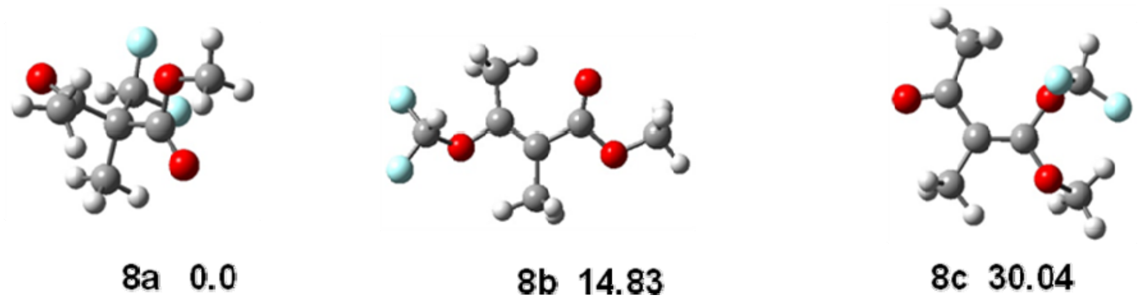

Figure 5S. Optimized geometries and relative energies calculated for the C- or O-alkylated products of MeCOCMeCOOMe$^-$ with $^+CHF_2$ at the MP2/6-311G** level. Energy in kcal/mol.

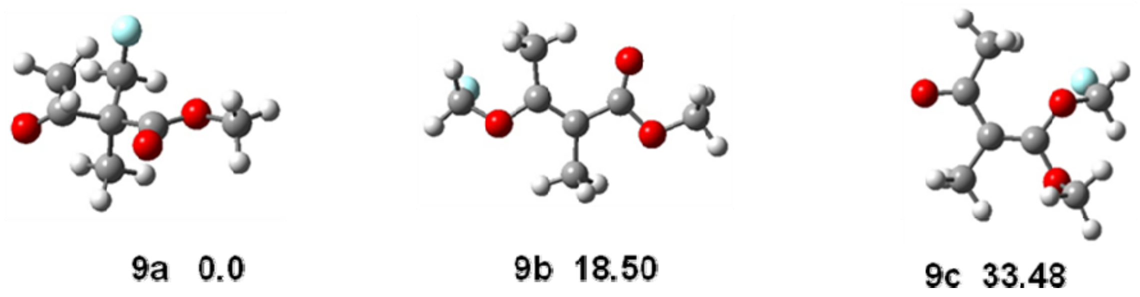

Figure 6S. Optimized geometries and relative energies calculated for the C- or O-alkylated products of MeCOCMeCOOMe$^-$ with $^+CH_2F$ at the MP2/6-311G** level. Energy in kcal/mol.

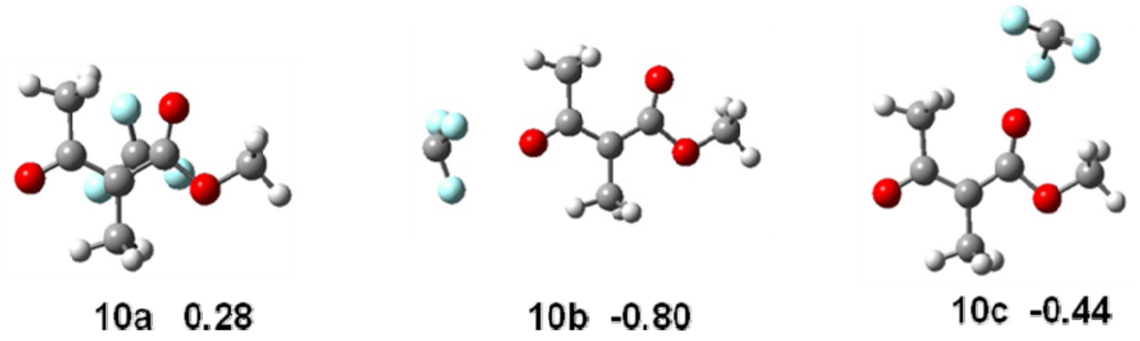

Figure 7S. Optimized geometries and stabilization energies calculated for the MeCOCMeCOOMe$^-$ complex with $\cdot CF_3$ radical at the MP2/6-311G** level. Energy in kcal/mol.

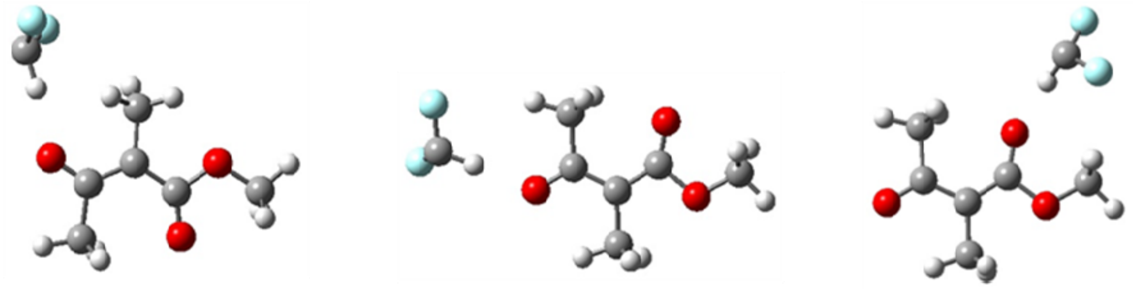

11a -8.37 11b -8.46 11c -7.70

Figure 8S. Optimized geometries and stabilization energies calculated for the $MeCOCMeCOOMe^-$ complex with $\cdot CHF_2$ radical at the MP2/6-311G** level. Energy in kcal/mol.

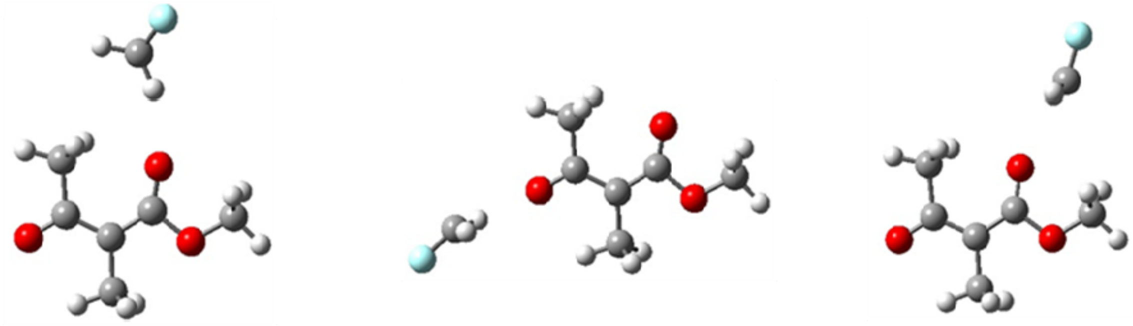

12a -5.66 12b -5.20 12c -4.85

Figure 9S. Optimized geometries and stabilization energies calculated for the $MeCOCMeCOOMe^-$ complex with $\cdot CH_2F$ radical at the MP2/6-311G** level. Energy in kcal/mol.

References

1. A. M. R. Smith, D. Billen, K. K. Hii, *Chem. Commun.*, **2009**, 3925–3927.
2. J. Frisch, G. W. Trucks, H. B. Schlegel, G. E. Scuseria, M. A. Robb, J. R. Cheeseman, J. A. Montgomery, Jr., T. Vreven, K. N. Kudin, J. C. Burant, J. M. Millam, S. S. Iyengar, J. Tomasi, V. Barone, B. Mennucci, M. Cossi, G. Scalmani, N. Rega, G. A. Petersson, H. Nakatsuji, M. Hada, M. Ehara, K. Toyota, R. Fukuda, J. Hasegawa, M. Ishida, T. Nakajima, Y. Honda, O. Kitao, H. Nakai, M. Klene, X. Li, J. E. Knox, H. P. Hratchian, J. B. Cross, V. Bakken, C. Adamo, J. Jaramillo, R. Gomperts, R. E. Stratmann, O. Yazyev, A. J. Austin, R. Cammi, C. Pomelli, J. W. Ochterski, P. Y. Ayala, K. Morokuma, G. A. Voth, P. Salvador, J. J. Dannenberg, V. G. Zakrzewski, S. Dapprich, A. D. Daniels, M. C. Strain, O. Farkas, D. K. Malick, A. D. Rabuck, K. Raghavachari, J. B. Foresman, J. V. Ortiz, Q. Cui, A. G. Baboul, S. Clifford, J. Cioslowski, B. B. Stefanov, G. Liu, A. Liashenko, P. Piskorz, I. Komaromi, R. L. Martin, D. J. Fox, T. Keith, M. A. Al–Laham, C. Y. Peng, A. Nanayakkara, M. Challacombe, P. M. W. Gill,

B. Johnson, W. Chen, M. W. Wong, C. Gonzalez, J. A. Pople, GAUSSIAN 03 (Revision E.01), Gaussian, Inc.,.Wallingford, CT, 2004.

3. C. Møller, M. S. Plesset, *Phys. Rev.* **1934**, *46*, 618–622.
4. M. Head-Gordon, J. A. Pople, M. J. Frisch, *Chem. Phys. Lett.* **1988**, *153,* 503–506.
5. B. J. Ransil, *J. Chem. Phys*. **1961**, *34,* 2109–2118.
6. S. F. Boys, F. Bernardi, *Mol. Phys*. **1970***, 19,* 553–566.
7. U, C, Singh, P. A. Kollman, *J. Comput. Chem.,* **1984**, *5*, 129–145.
8. B. H. Besler, K. M. Mertz, P. A. Kollman, *J. Comput. Chem.,* **1990**, *11,* 431–439.

General procedure

To a stirred solution of N-methyl-S-difluoromethyl-S-phenyl-sulfoximine (61.0 mg, 0.3 mmol) in anhydrous CH_2Cl_2 (0.2 mL), trimethyloxonium tetrafluoroborate (49.0 mg, 0.33 mol) was added under N_2. The reaction mixture was stirred for 1 h and then cooled to –78 °C. A solution of substrate **1a** (19.0 mg, 0.1 mmol) and P_1-*t*Bu (63 μL, 0.25 mmol) in anhydrous CH_2Cl_2 (0.4 mL) which had been stirred over 15 min at room temperature was added to the above mixture. Then this mixture was stirred over further 3 h under –78 °C. The solvent was evaporated under vacuum and the residue was purified through column chromatography on silica gel (Hexane/EtOAc = 80/20) to give difluoromethylated compounds **3a** and **4a**. Pure **3a** and **4a** were afforded by further purified through column chromatography on silica gel (Toluene for **3a** and Hexane/CH_2Cl_2 = 3/1 to 1/1 for **4a**), respectively.

Methyl 2-(difluoromethyl)-1-oxo-2,3-dihydro-1H-indene-2-carboxylate (3a).

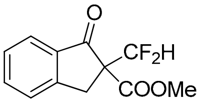

Purified through column chromatography on silica gel (Hexane/EtOAc = 80/20) to give **3a** and **4a.** Further purification was afforded pure **3a** (10.0 mg, 42%) through column chromatography on silica gel (Toluene) as a colorless oil.

^{1}H NMR (300 MHz, $CDCl_3$) δ 7.78 (d, *J* = 7.5 Hz, 1H), 7.68 (t, *J* = 7.2 Hz, 1H), 7.58-7.56 (m, 1H), 7.43 (t, *J* = 7.5 Hz, 1H), 6.60 (t, *J* = 55.2 Hz, 1H), 3.97 (s, 3H), 3.73, 3.56 (AB quartet, *J* = 17.4 Hz, 2H); ^{13}C NMR (150.9MHz, $CDCl_3$) δ 195.9 (d, *J* = 7.5 Hz), 166.6 (d, *J* = 12.0 Hz), 154.0, 136.3, 134.1 (d, *J* = 3.0 Hz), 128.3, 126.6, 125.5, 115.6 (t, *J* = 244.4 Hz), 64.7 (t, *J* = 24.1 Hz), 53.6, 30.0 (d, *J* = 3.0 Hz); ^{19}F NMR (282 MHz, $CDCl_3$) δ –126.5 (dd, *J* = 287.9, 55.5 Hz, 1F), –129.8 (dd, *J* = 287.0, 55.5 Hz, 1F); IR (neat) 3007, 2958, 1748, 1720, 1606, 1590, 1465, 1213, 1076, 1046, 758 cm^{-1} ; MS (ESI, m/z) 263.0 $[M+Na]^+$; HRMS (ESI) calcd. for $C_{12}H_{10}F_2O_3Na$ $[M+Na]^+$: 263.0496, Found: 263.0503.

Methyl 3-(difluoromethoxy)-1H-indene-2-carboxylate (4a)

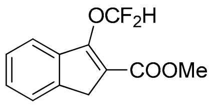

Further purification was afforded pure **4a** (5.3 mg, 22%) through column chromatography on silica gel (Hexane/CH_2Cl_2 = 3/1 to 1/1) as a white solid.

m.p.: 60–61 °C; ^{1}H NMR (300 MHz, $CDCl_3$) δ 7.65-7.63 (m, 1H), 7.49–7.38 (m, 3H, partly overlapping signal), 7.18 (t, J = 75.0 Hz, 1H, partly overlapping signal), 3.89 (s, 3H), 3.73 (s, 2H); ^{13}C NMR (150.9MHz, $CDCl_3$) δ 164.1, 156.2, 141.6, 138.6, 129.4, 127.4, 124.5, 121.2, 117.0, 116.7 (t, J = 262.1 Hz), 51.9, 36.2; ^{19}F NMR (282 MHz, $CDCl_3$) δ –82.3 (d, J = 74.4 Hz); IR (KBr) 3094, 2954, 1708, 1616, 1598, 1578, 1469, 1400, 1354, 1266, 1152, 1099, 761 cm^{-1}; MS (ESI, m/z) 263.3 $[M+Na]^+$; HRMS (ESI) calcd. for $C_{12}H_{10}F_2O_3Na$ $[M+Na]^+$: 263.0496, Found: 263.0502.

Ethyl 2-(difluoromethyl)-1-oxo-2,3-dihydro-1H-indene-2-carboxylate (3b)

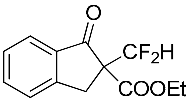

Purified through column chromatography on silica gel (Hexane/EtOAc = 85/15) to give **3b** and **4b.** Further purification was afforded pure **3b** (8.7 mg, 34%) through column chromatography on silica gel (Toluene) as a colorless oil.

^{1}H NMR (300 MHz, $CDCl_3$) δ 7.78 (d, J = 7.5 Hz, 1H), 7.68 (t, J = 7.5 Hz, 1H), 7.57 (d, J = 7.5 Hz, 1H), 7.43 (t, J = 7.5 Hz, 1H), 6.60 (t, J = 55.5 Hz, 1H), 4.26 (q, J = 7.5 Hz, 2H), 3.73, 3.55 (AB quartet, J = 17.7 Hz, 2H), 1.27 (t, J = 6.9 Hz, 3H); ^{13}C NMR (150.9MHz, $CDCl_3$) δ 196.0, 166.0 (d, J = 12.0 Hz), 154.1, 136.2, 134.2 (d, J = 4.5 Hz), 128.2, 126.6, 125.4, 115.6 (t, J = 247.5 Hz), 64.8 (t, J = 22.6 Hz), 62.8, 30.0, 14.11; ^{19}F NMR (282 MHz, $CDCl_3$) δ –126.6 (dd, J = 287.0, 54.4 Hz, 1F), –129.7 (dd, J = 287.0, 55.5 Hz, 1F); IR (neat) 2985, 2939, 1746, 1721, 1607, 1590, 1433, 1370, 1260, 1076, 757 cm^{-1}; MS (ESI, m/z) 277.2 $[M+Na]^+$; HRMS (ESI) calcd. for $C_{13}H_{12}F_2O_3Na$ $[M+Na]^+$: 277.0652, Found: 277.0663.

Ethyl 3-(difluoromethoxy)-1H-indene-2-carboxylate (4b)

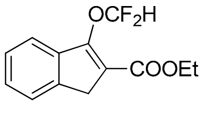

Further purification was afforded pure **4b** (4.9 mg, 19%) through column chromatography on silica gel (Hexane/CH_2Cl_2 = 3/1 to 1/1) as a yellow solid.

m.p.: 40–42 °C; ^{1}H NMR (300 MHz, $CDCl_3$) δ 7.65–7.62 (m, 1H), 7.49–7.38 (m, 3H, partly overlapping signal), 7.16 (t, *J* = 75 Hz, 1H, partly overlapping signal), 4.32 (q, *J* = 7.2 Hz, 2H), 3.73 (s, 2H), 1.37 (t, *J* =6.9 Hz, 3H); ^{13}C NMR (150.9MHz, $CDCl_3$) δ 163.6, 156.0, 141.6, 138.7, 129.3, 127.4, 124.5, 121.2, 117.6, 116.7 (t, *J* = 260.1 Hz), 60.9, 36.2, 14.4; ^{19}F NMR (282 MHz, $CDCl_3$) δ –82.1 (d, *J* = 75.0 Hz); IR (KBr) 2982, 2932, 1693, 1617, 1600, 1580, 1478, 1387, 1256, 1164, 1119, 761 cm^{-1}; MS (ESI, m/z) 277.3 $[M+Na]^+$; HRMS (ESI) calcd. for $C_{13}H_{12}F_2O_3Na$ $[M+Na]^+$: 277.0652, Found: 277.0653.

tert-Butyl 2-(difluoromethyl)-1-oxo-2,3-dihydro-1H-indene-2-carboxylate (3c)

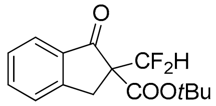

Purified through column chromatography on silica gel (Hexane/EtOAc = 90/10) to give **3c** and **4c.** Further purification was afforded pure **3c** (7.8 mg, 28%) through column chromatography on silica gel (Toluene) as a white semi-solid.

^{1}H NMR (300 MHz, $CDCl_3$) δ 7.77 (d, *J* = 7.8 Hz, 1H), 7.68–7.63 (m, 1H), 7.56–7.54 (m, 1H), 7.43–7.38 (m, 1H), 6.54 (t, *J* = 55.2 Hz, 1H), 3.67, 3.50 (AB quartet, *J* = 17.7 Hz, 2H), 1.50 (s, 9H); ^{13}C NMR (150.9MHz, $CDCl_3$) δ 196.4 (d, *J* = 7.5 Hz), 164.8 (d, *J* = 12.0 Hz), 154.1, 136.1, 134.3 (d, *J* = 4.5 Hz), 128.0, 126.6, 125.3, 115.8 (t, *J* = 247.5 Hz), 84.0, 65.6 (t, *J* = 24.1 Hz), 29.9, 27.9; ^{19}F NMR (282 MHz, $CDCl_3$) δ –127.1 (dd, *J* = 285.9, 54.4 Hz, 1F), –129.2 (dd, *J* = 287.0, 56.4 Hz, 1F); IR (KBr) 2987, 1711, 1604, 1588, 1465, 1372, 1272, 1147, 1069, 841, 741 cm^{-1}; MS (ESI, m/z) 305.6 $[M+Na]^+$; HRMS (ESI) calcd. for $C_{15}H_{16}F_2O_3Na$ $[M+Na]^+$: 305.0965, Found: 305.0968.

tert-Butyl 3-(difluoromethoxy)-1H-indene-2-carboxylate (4c)

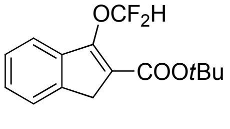

Further purification was afforded pure **4c** (4.3 mg, 15%) through column chromatography on silica gel (Hexane/CH_2Cl_2 = 3/1 to 1/1) as a slightly yellow semi-solid.

^{1}H NMR (300 MHz, $CDCl_3$) δ 7.62–7.61 (m, 1H), 7.46–7.38 (m, 2H), 7.08 (t, *J* = 75.3 Hz, 1H), 3.67 (s, 3H), 1.56 (s, 9H); ^{13}C NMR (150.9MHz, $CDCl_3$) δ 163.0, 154.8, 141.5, 139.0, 129.0, 127.3, 124.4, 121.0, 120.0, 116.7 (t, *J* = 261.0 Hz, 1H), 81.8, 36.4, 28.4; ^{19}F NMR (282 MHz, $CDCl_3$) δ –82.0 (d, *J* = 75.0 Hz); IR (KBr) 2985, 2933, 1696, 1601, 1580, 1456, 1369, 1353, 1267, 1164, 1128, 1047, 757 cm^{-1}; MS (ESI, m/z) 305.2 $[M+Na]^+$; HRMS (ESI) calcd. for $C_{15}H_{16}F_2O_3Na$ $[M+Na]^+$: 305.0965, Found: 305.0967.

Methyl 5-bromo-2-(difluoromethyl)-1-oxo-2,3-dihydro-1H-indene-2-carboxylate (3d)

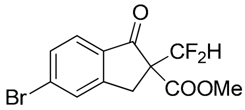

Purified through column chromatography on silica gel (Hexane/EtOAc = 99/1) to give **3d** and **4d.** Further purification was afforded pure **3d** (12.4 mg, 39%) through column chromatography on silica gel (Toluene) as a white solid.

m.p.: 87–88 °C; ^{1}H NMR (300 MHz, $CDCl_3$) δ 7.76 (s, 1H), 7.60 (dd, *J* = 19.8, 8.4 Hz, 2H), 6.58 (t, *J* = 55.2 Hz, 1H), 3.80 (s, 3H), 3.71, 3.54 (AB quartet, *J* = 18.0 Hz, 2H); ^{13}C NMR (150.9MHz, $CDCl_3$) δ 194.7 (d, *J* = 6.0 Hz), 166.1 (d, *J* = 12.1 Hz), 155.4, 133.0 (d, *J* = 3.0 Hz), 132.0 (d, *J* = 7.5 Hz), 130.0, 126.5, 115.3 (t, *J* = 245.9 Hz), 64.8 (t, *J* = 21.1 Hz), 53.7, 29.7; ^{19}F NMR (282 MHz, $CDCl_3$) δ –126.4 (dd, *J* = 287.9, 54.4 Hz, 1F), –129.6 (dd, *J* = 288.2, 55.5 Hz, 1F); IR (KBr) 2965, 1752, 1714, 1598, 1576, 1432, 1323, 1272, 1261, 1167, 1082, 827 cm^{-1}; MS (ESI, m/z) 341.3 $[M+Na]^+$; HRMS (ESI) calcd. for $C_{12}H_9F_2O_3NaBr$ $[M+Na]^+$: 340.9601, Found: 340.9610.

Methyl 6-bromo-3-(difluoromethoxy)-1H-indene-2-carboxylate (4d)

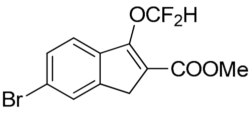

Further purification was afforded pure **4d** (7.0 mg, 22%) through column chromatography on silica gel (Hexane/CH_2Cl_2 = 3/1 to 1/1) as a white solid.

m.p.: 83–85 °C; ^{1}H NMR (300 MHz, $CDCl_3$) δ 7.63 (s, 1H), 7.52 (dd, *J* = 19.5, 8.4 Hz, 2H), 7.19 (t, *J* = 75.3 Hz, 1H), 3.85 (s, 3H), 3.70 (s, 2H); ^{13}C NMR (150.9MHz, $CDCl_3$) δ 163.8, 155.4, 143.3, 137.6, 130.8, 127.9, 124.0, 122.5, 117.1, 116.5 (t, *J* = 262.6 Hz), 52.1, 36.0; ^{19}F NMR (282 MHz, $CDCl_3$) δ –82.5 (d, *J* = 74.2 Hz); IR (KBr) 3030, 2954, 2927, 1610, 1593, 1568, 1434, 1360, 1323, 1255, 1126, 1085, 1046, 835 cm^{-1}; MS (ESI, m/z) 341.5 $[M+Na]^+$; HRMS (ESI) calcd. for $C_{12}H_9F_2O_3NaBr$ $[M+Na]^+$: 340.9601, Found: 340.9600.

Methyl 5-chloro-2-(difluoromethyl)-1-oxo-2,3-dihydro-1H-indene-2-carboxylate (3e)

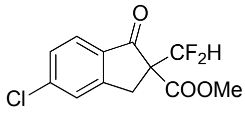

Purified through column chromatography on silica gel (Hexane/EtOAc = 99/1) to give pure **3e** (11.0 mg, 40%, slightly yellow solid) and **4e.**

m.p.: 88–89 °C; ^{1}H NMR (300 MHz, $CDCl_3$) δ 7.71 (d, *J* = 8.1 Hz, 1H), 7.57 (s, 1H), 7.41 (d, *J* = 8.4 Hz, 1H), 6.58 (t, *J* = 55.2 Hz, 1H), 3.80 (s, 3H), 3.70, 3.53 (AB quartet, *J* = 18.0 Hz, 2H); ^{13}C

NMR (150.9MHz, $CDCl_3$) δ 194.4 (d, J = 6.0 Hz), 166.2 (d, J = 12.1 Hz), 155.3, 143.2, 132.6 (d, J = 4.5 Hz), 129.2, 126.9, 126.5, 115.3 (t, J = 244.4 Hz), 64.9 (t, J = 21.1 Hz), 53.7, 29.7; ^{19}F NMR (282 MHz, $CDCl_3$) δ –126.4 (dd, J = 286.8, 54.4 Hz, 1F), –129.6 (dd, J = 287.9, 55.3 Hz, 1F); IR (KBr) 2965, 1752, 1714, 1600, 1579, 1430, 1375, 1326, 1274, 1257, 1082, 1045, 834, 707 cm^{-1}; MS (ESI, m/z) 297.1 $[M+Na]^+$; HRMS (ESI) calcd. for $C_{12}H_9F_2O_3NaCl$ $[M+Na]^+$: 297.0106, Found: 297.0106.

Methyl 6-chloro-3-(difluoromethoxy)-1H-indene-2-carboxylate (4e)

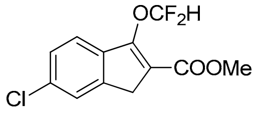

Further purification was afforded pure **4e** (7.2 mg, 26%) through column chromatography on silica gel (Hexane/CH_2Cl_2 = 3/1 to 1/1) as a white solid.

m.p.: 88–90 °C; ^1H NMR (300 MHz, $CDCl_3$) δ 7.56–7.38 (m, 3H), 7.19 (t, J = 75.0 Hz, 1H), 3.85 (s, 3H), 3.71 (s, 2H); ^{13}C NMR (150.9MHz, $CDCl_3$) δ 163.8, 155.3, 143.0, 137.2, 135.8, 128.0, 125.0, 122.2, 117.2, 116.5 (t, J = 262.6 Hz), 52.1, 36.0; ^{19}F NMR (282 MHz, $CDCl_3$) δ –82.4 (d, J = 75.3 Hz); IR (KBr) 2957, 1702, 1618, 1571, 1434, 1357, 1260, 1123, 1065, 820 cm^{-1}; MS (ESI, m/z) 297.3 $[M+Na]^+$; HRMS (ESI) calcd. for $C_{12}H_9F_2O_3NaCl$ $[M+Na]^+$: 297.0106, Found: 297.0114.

Methyl 2-(difluoromethyl)-6-methyl-1-oxo-2,3-dihydro-1H-indene-2-carboxylate (3f)

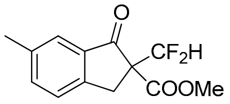

Purified through column chromatography on silica gel (Hexane/EtOAc = 80/20) to give **3f** and **4f.** Further purification was afforded pure **3f** (10.6 mg, 42%) through column chromatography on silica gel (Toluene) as a white solid.

m.p.: 64-66 °C; ^1H NMR (300 MHz, $CDCl_3$) δ 7.57 (s, 1H), 7.47 (dd, J = 15.9, 8.4 Hz, 2H), 6.58 (t, J = 54.9 Hz, 1H), 3.78 (s, 3H), 3.66, 3.50 (AB quartet, J = 17.4 Hz, 2H), 2.41 (s, 3H); ^{13}C NMR (150.9MHz, $CDCl_3$) δ 195.9 (d, J = 6.0 Hz), 166.7 (d, J = 12.1 Hz), 151.5, 138.4, 137.6, 134.3 (d, J = 10.6 Hz), 126.3, 125.3, 115.6 (t, J = 244.5 Hz), 65.0 (t, J = 22.6 Hz), 53.5, 29.7, 21.1; ^{19}F NMR (282 MHz, $CDCl_3$) δ –126.5 (dd, J = 287.1, 54.4 Hz, 1F),–129.9 (dd, J = 286.8, 55.3 Hz, 1F); IR (KBr) 3031, 2959, 1617, 1583, 1494, 1381, 1284, 1212, 1078, 1042, 824 cm^{-1}; MS (ESI, m/z) 277.0 $[M+Na]^+$; HRMS (ESI) calcd. for $C_{13}H_{12}F_2O_3Na$ $[M+Na]^+$: 277.0652, Found: 277.0661.

Methyl 3-(difluoromethoxy)-5-methyl-1H-indene-2-carboxylate (4f)

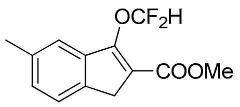

Further purification was afforded pure **4f** (5.2 mg, 20%) through column chromatography on silica gel (Hexane/CH_2Cl_2 = 3/1 to 1/1) as a white solid.

m.p.: 62–64 °C; ^{1}H NMR (300 MHz, $CDCl_3$) δ 7.44-7.23 (m, 3H, partly overlapping signal), 7.17 (t, *J* = 75.0 Hz, 1H, partly overlapping signal), 3.85 (s, 3H), 3.67 (s, 2H), 2.44 (s, 3H); ^{13}C NMR (150.9MHz, $CDCl_3$) δ 164.1, 156.3 (d, *J* = 4.5 Hz), 138.9, 138.8, 137.4, 130.5, 124.2, 121.5, 117.1, 116.7 (t, *J* = 262.6 Hz), 51.9, 35.8, 21.6; ^{19}F NMR (282 MHz, $CDCl_3$) δ –82.2 (d, *J* = 75.0 Hz); IR (KBr) 2952, 1704, 1623, 1601, 1578, 1436, 1355, 1255, 1200, 1145, 1025, 823 cm^{-1}; MS (ESI, m/z) 277.2 $[M+Na]^+$; HRMS (ESI) calcd. for $C_{13}H_{12}F_2O_3Na$ $[M+Na]^+$: 277.0652, Found: 277.0679.

Methyl 2-(difluoromethyl)-6-methoxy-1-oxo-2,3-dihydro-1H-indene-2-carboxylate (3g)

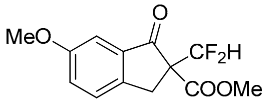

Purified through column chromatography on silica gel (Hexane/EtOAc = 80/20) to give **3g** and **4g.** Further purification was afforded pure **3g** (11.0 mg, 41%) through column chromatography on silica gel (Toluene) as a white solid.

m.p.: 77–78 °C; ^{1}H NMR (300 MHz, $CDCl_3$) δ 7.46 (d, *J* = 8.4 Hz, 1H), 7.28–7.18 (m, 2H), 6.58 (t, *J* = 54.9 Hz, 1H), 3.84 (s, 3H), 3.79 (s, 3H), 3.63, 3.47 (AB quartet, *J* = 17.1 Hz, 2H); ^{13}C NMR (150.9MHz, $CDCl_3$) δ 195.8 (d, *J* = 7.5 Hz), 166.6 (d, *J* = 12.1 Hz), 160.0, 147.1, 135.3 (d, *J* = 4.5 Hz), 127.3, 126.0, 115.5 (t *J* = 244.5 Hz), 106.2, 65.4 (t, *J* = 22.6 Hz), 55.8, 53.6, 29.4; ^{19}F NMR (282 MHz, $CDCl_3$) δ –126.5 (dd, *J* = 286.8, 54.4 Hz, 1F), –130.0 (dd, *J* = 288.2, 55.6 Hz, 1F); IR (KBr) 3041, 2957, 2834, 1743, 1709, 1617, 1492, 1451, 1434, 1313, 1279, 1200, 1074, 845, 763 cm^{-1}; MS (ESI, m/z) 293.1 $[M+Na]^+$; HRMS (ESI) calcd. for $C_{13}H_{12}F_2O_4Na$ $[M+Na]^+$: 293.0601, Found: 293.0617.

Methyl 3-(difluoromethoxy)-5-methoxy-1H-indene-2-carboxylate (4g)

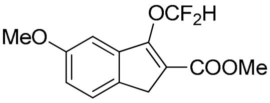

Further purification was afforded pure **4g** (5.5 mg, 20%) through column chromatography on silica gel (Hexane/CH_2Cl_2 = 3/1 to 1/1) as a white solid.

m.p.: 62–64 °C; ^{1}H NMR (300 MHz, $CDCl_3$) δ 7.44-6.94 (m, 4H), 3.87 (s, 3H), 3.85 (s, 3H), 3.66 (s, 2H); ^{13}C NMR (150.9MHz, $CDCl_3$) δ 164.0, 159.6, 156.1, 139.9, 133.9, 125.2, 118.0, 117.2, 116.7 (t,

J = 262.6 Hz), 104.8, 55.7, 51.9, 35.5; ^{19}F NMR (282 MHz, $CDCl_3$) δ –82.2 (d, *J* = 75.3 Hz); IR (KBr) 2949, 1706, 1605, 1580, 1487, 1356, 1260, 1231, 1129, 1082, 808, 745 cm^{-1}; MS (ESI, m/z) 293.2 $[M+Na]^+$; HRMS (ESI) calcd. for $C_{13}H_{12}F_2O_4Na$ $[M+Na]^+$: 293.0601, Found: 293.0603.

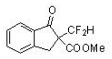
O
CF2H
COOMe

3a

^{1}H NMR

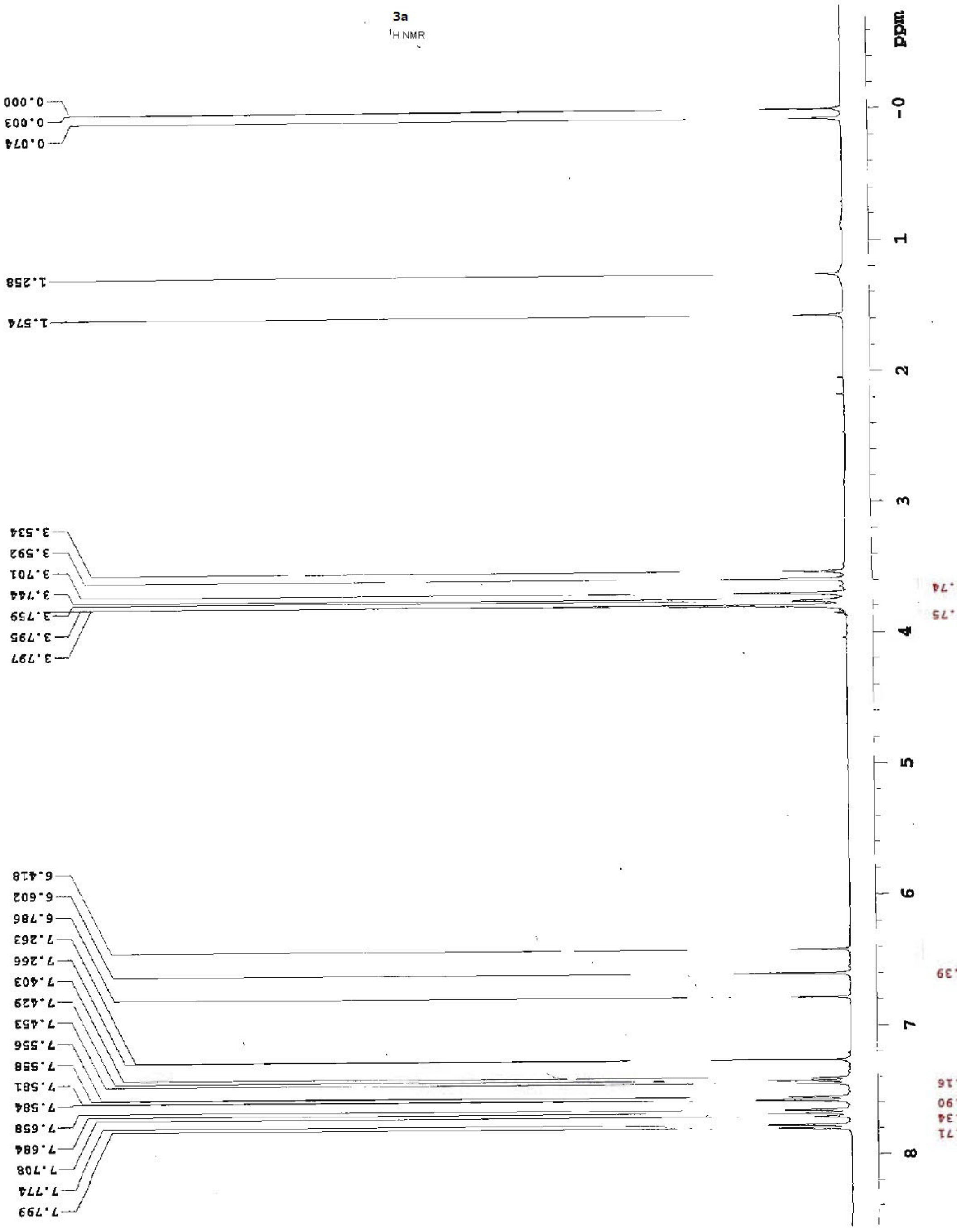
ppm
0
1
2
3
4
5
6
7
8
0.000
0.003
0.074
1.258
1.574
3.534
3.592
3.701
3.744
3.759
3.795
3.797
6.418
6.602
6.786
7.263
7.266
7.403
7.429
7.453
7.556
7.558
7.581
7.584
7.658
7.684
7.708
7.774
7.799
23.74
27.75
10.39
10.16
9.90
9.34
8.71

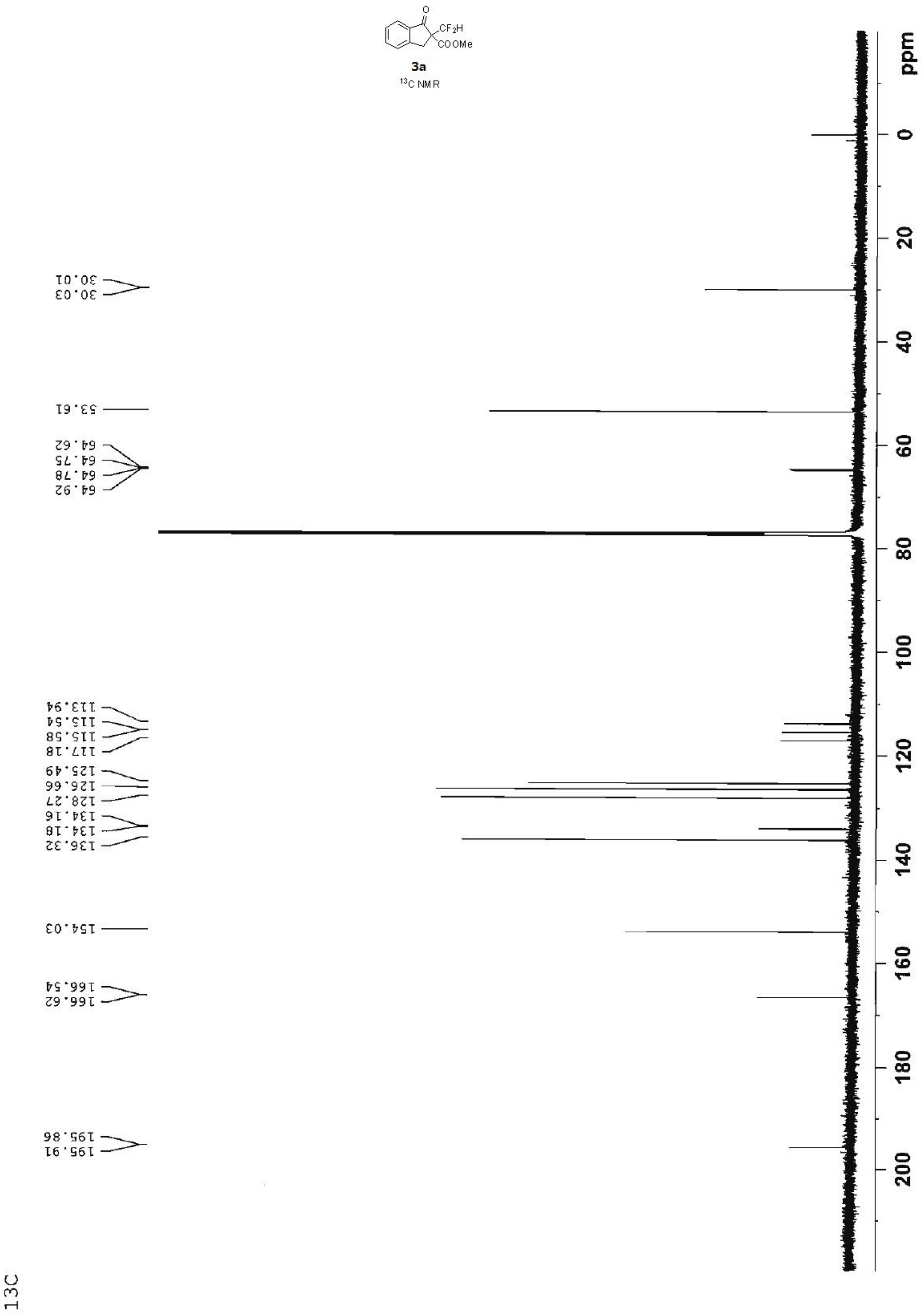
3a
13C NMR
13C
ppm
30.01
30.03
53.61
64.62
64.75
64.78
64.92
113.94
115.54
115.58
117.18
125.49
126.66
128.27
134.16
134.18
136.32
154.03
166.54
166.62
195.86
195.91

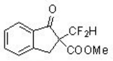

O
CF_2H
COOMe

3a

^{19}F NMR

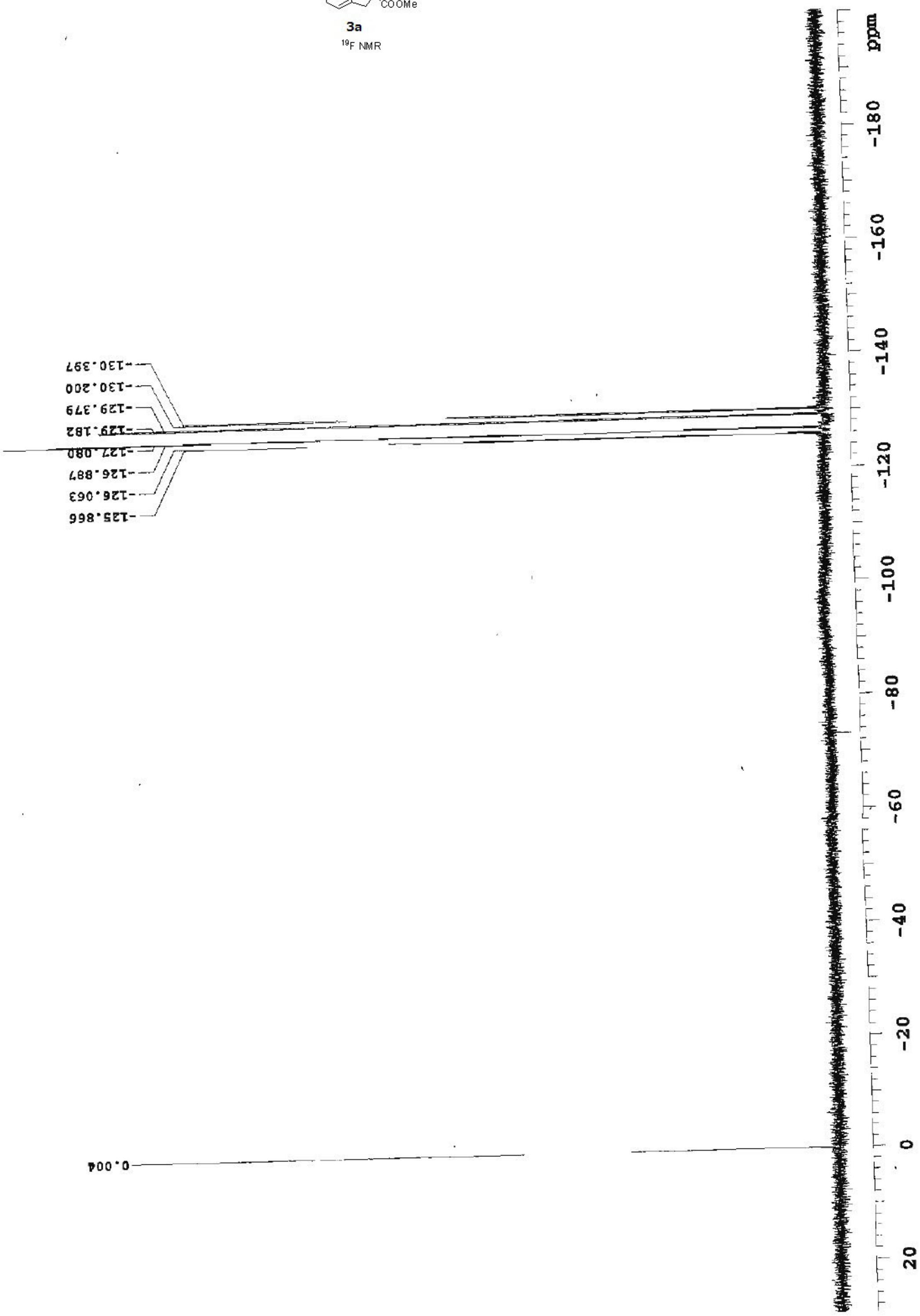

-130.397
-130.200
-129.379
-129.182
-127.080
-126.887
-126.063
-125.866
0.004
ppm
-180
-160
-140
-120
-100
-80
-60
-40
-20
0
20

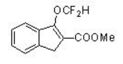

4a

^{1}H NMR

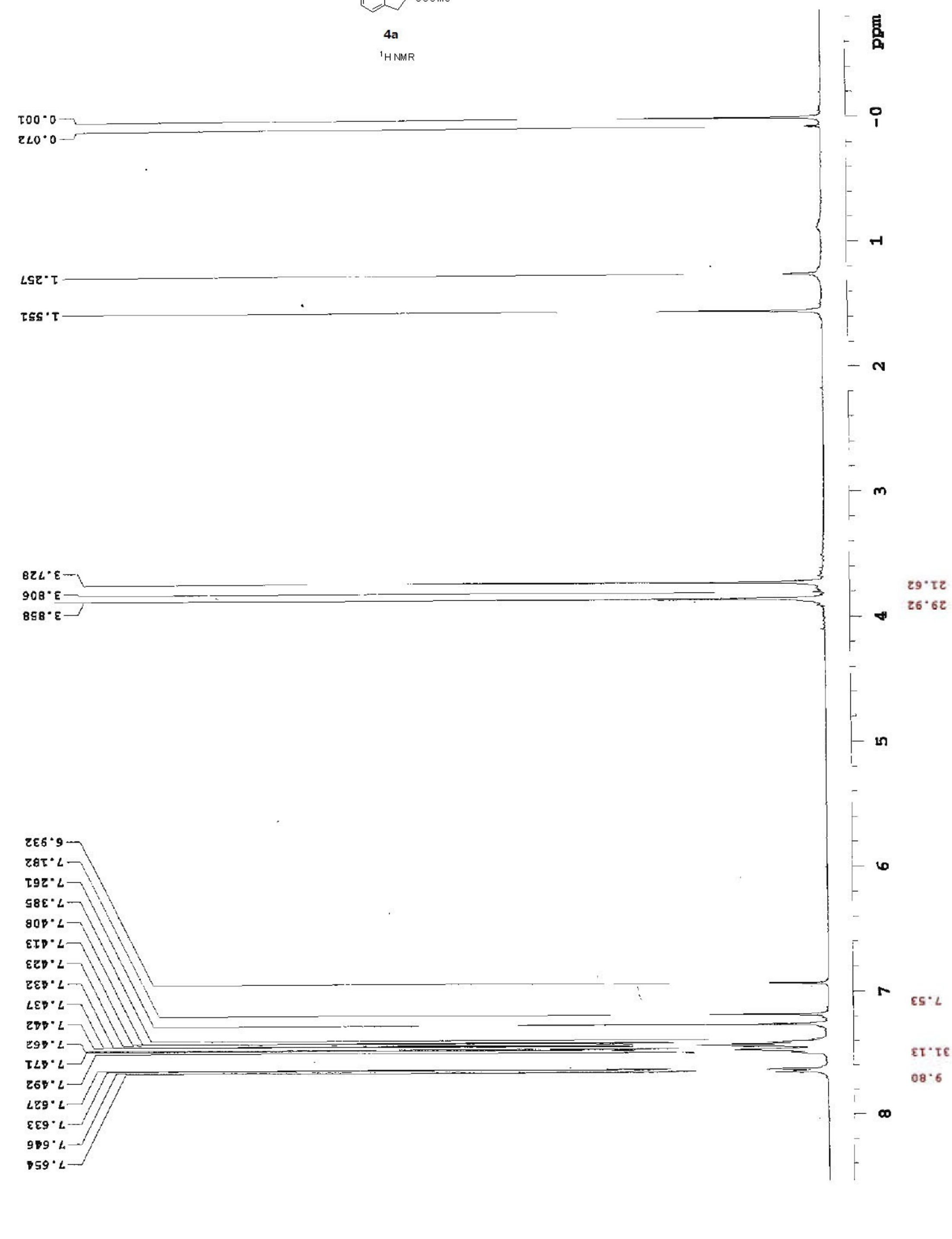
0.001
0.073
1.257
1.551
3.728
3.806
3.858
6.932
7.182
7.261
7.385
7.408
7.413
7.423
7.432
7.437
7.442
7.462
7.471
7.492
7.627
7.633
7.646
7.654
21.62
29.92
7.53
31.13
9.80
ppm

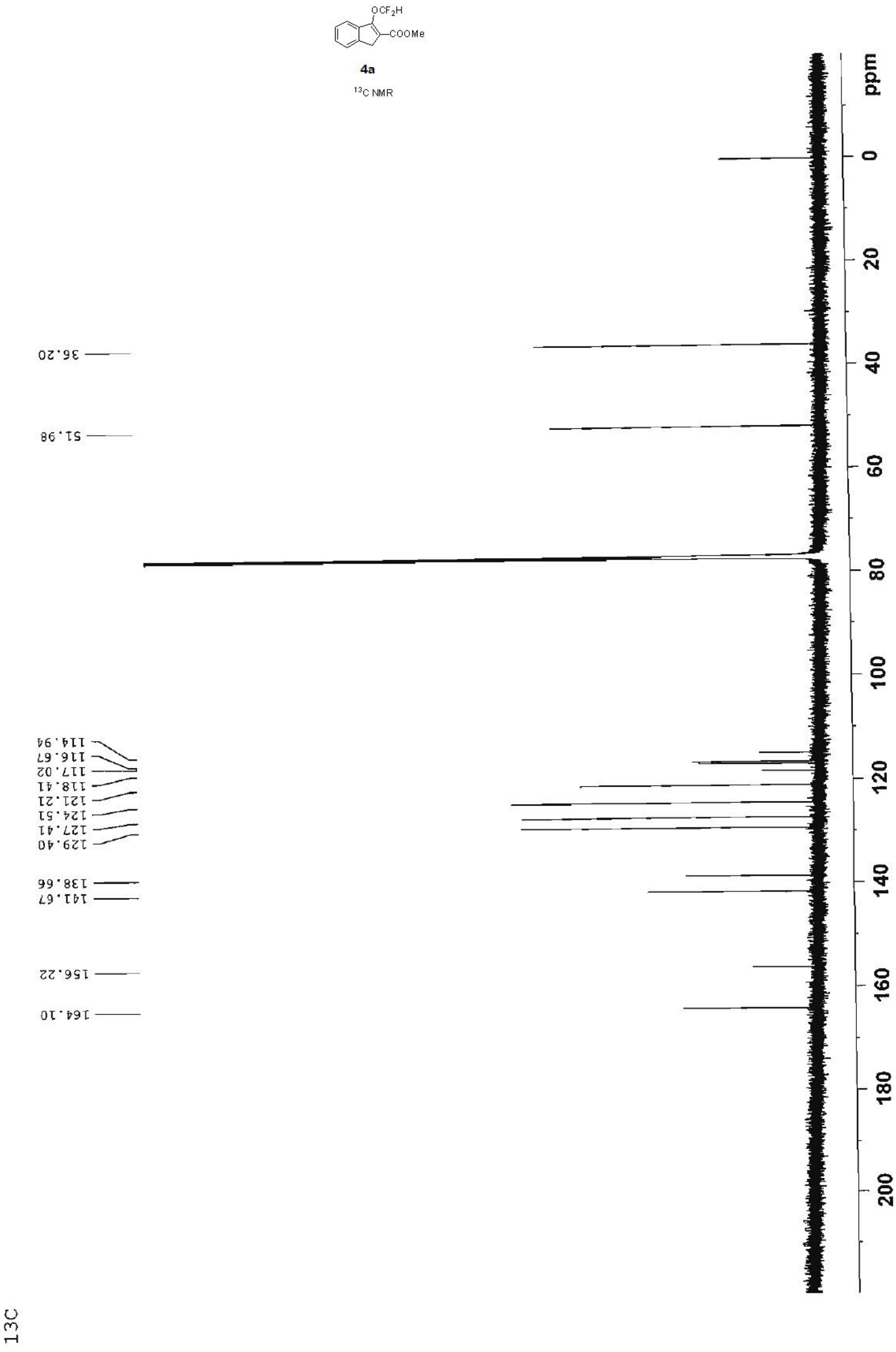
OCF2H
COOMe
4a
13C NMR
ppm
0
20
40
60
80
100
120
140
160
180
200
36.20
51.98
114.94
116.67
117.02
118.41
121.21
124.51
127.41
129.40
138.66
141.67
156.22
164.10
13C

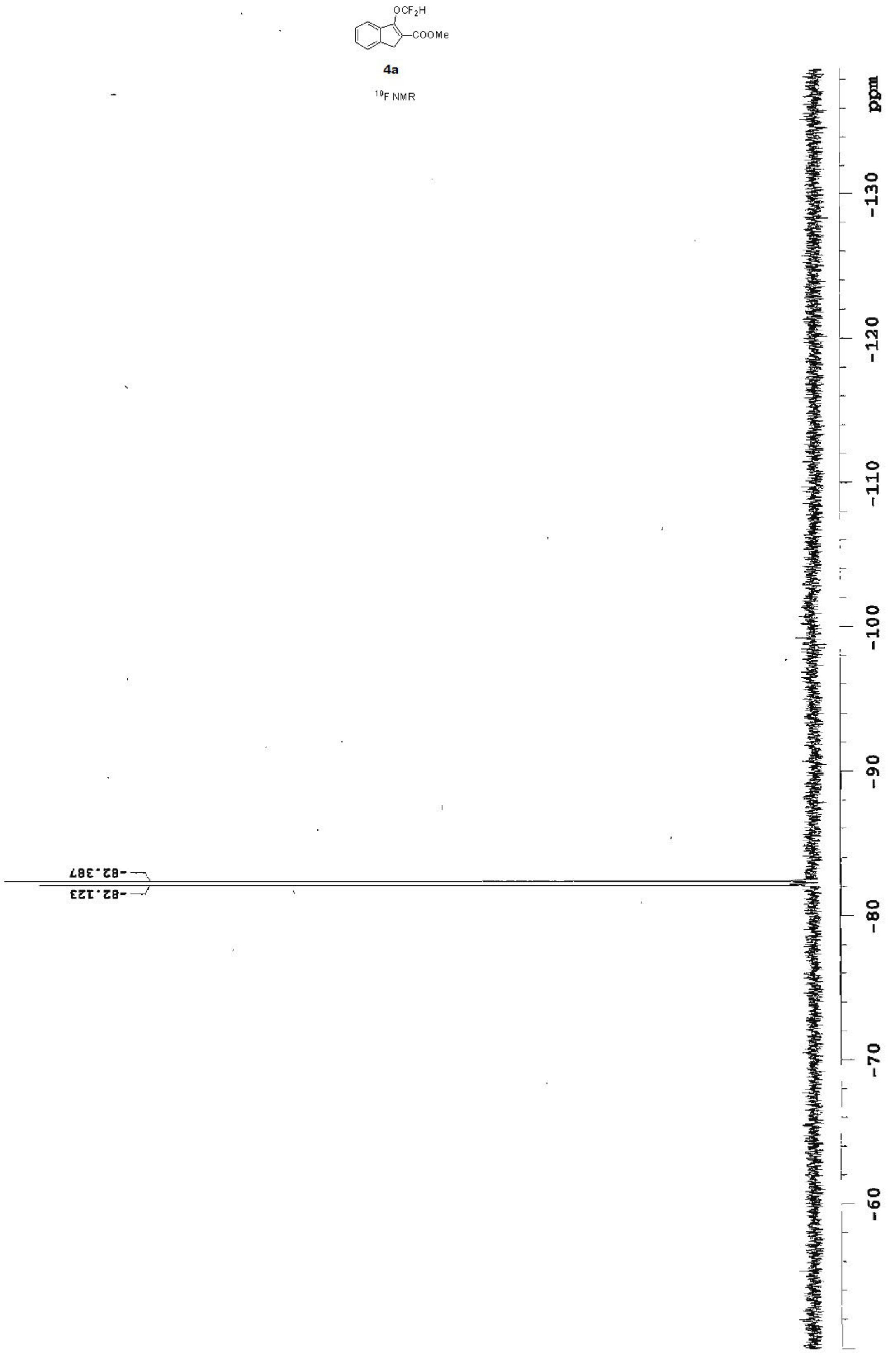
OCF2H
COOMe
4a
19F NMR
-82.387
-82.123
ppm
-130
-120
-110
-100
-90
-80
-70
-60

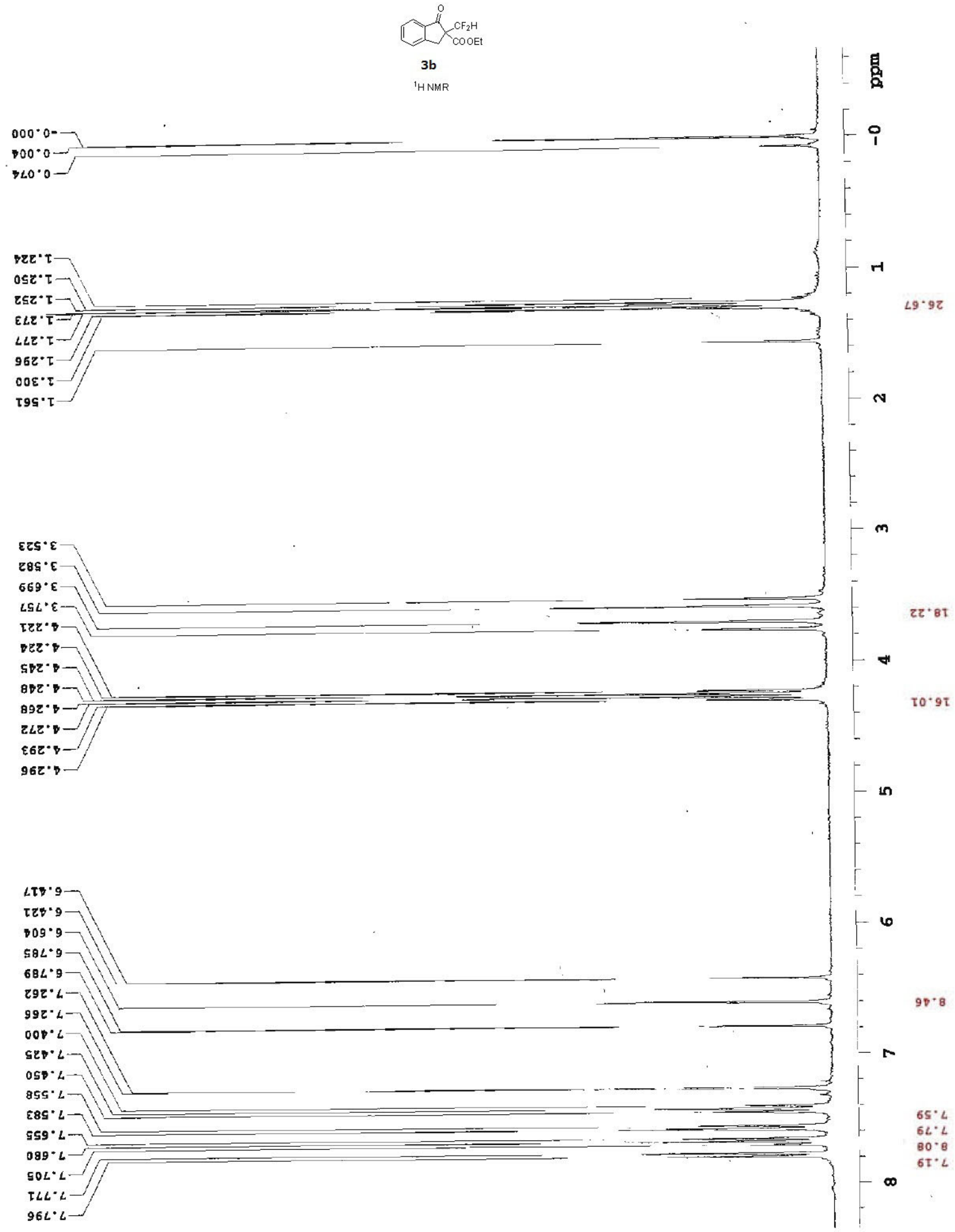
O
CF2H
COOEt
3b
1H NMR
-0.000
0.004
0.074
1.224
1.250
1.252
1.273
1.277
1.296
1.300
1.561
3.523
3.582
3.699
3.757
4.221
4.224
4.245
4.248
4.268
4.272
4.293
4.296
6.417
6.421
6.604
6.785
6.789
7.262
7.266
7.400
7.425
7.450
7.558
7.583
7.655
7.680
7.705
7.771
7.796
26.67
18.22
16.01
8.46
7.59
7.79
8.08
7.19
ppm
-0
1
2
3
4
5
6
7
8

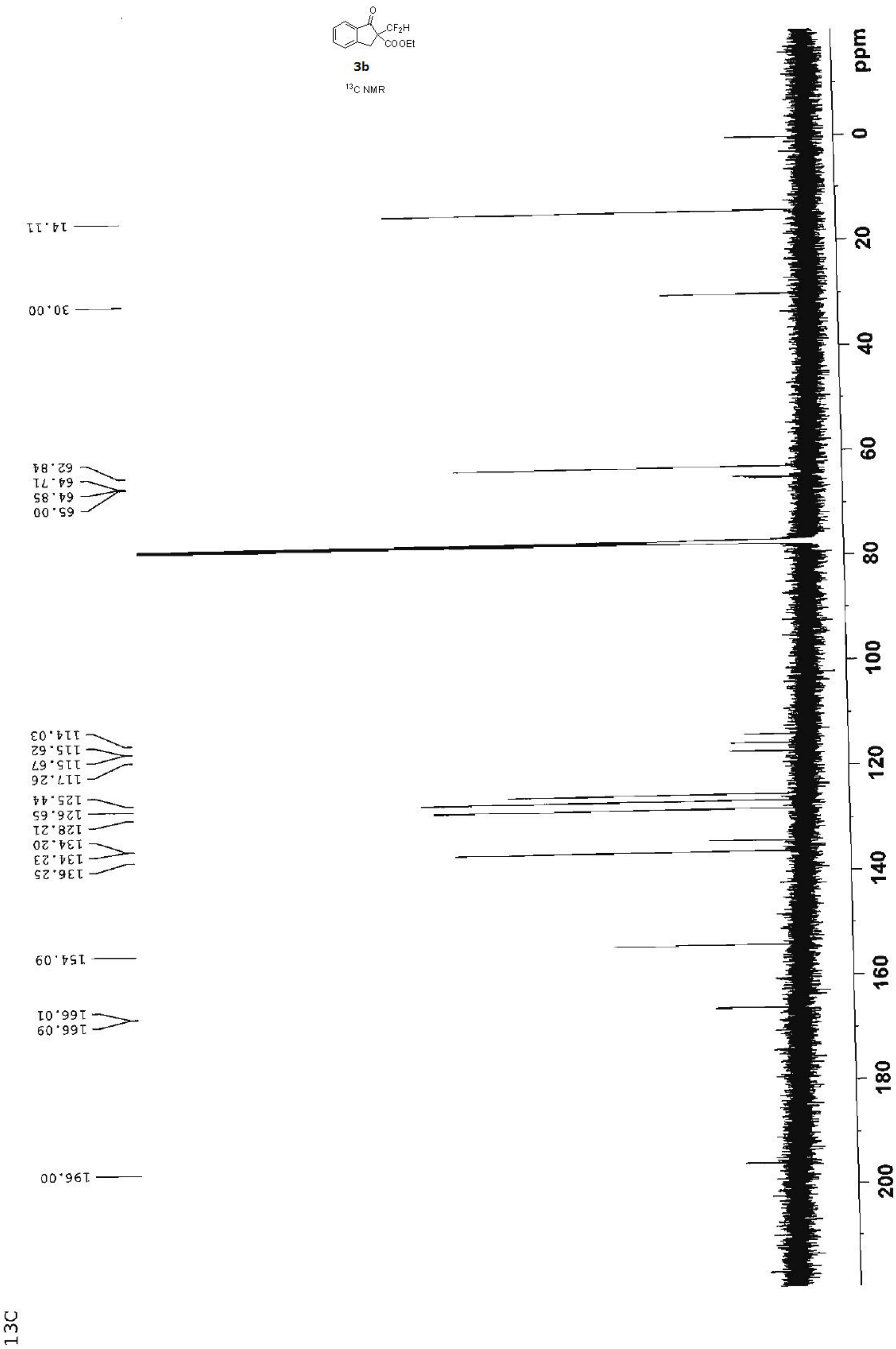
CF2H
COOEt
3b
13C NMR
13C
14.11
30.00
62.84
64.71
64.85
65.00
114.03
115.62
115.67
117.26
125.44
126.65
128.21
134.20
134.23
136.25
154.09
166.01
166.09
196.00
ppm
0
20
40
60
80
100
120
140
160
180
200

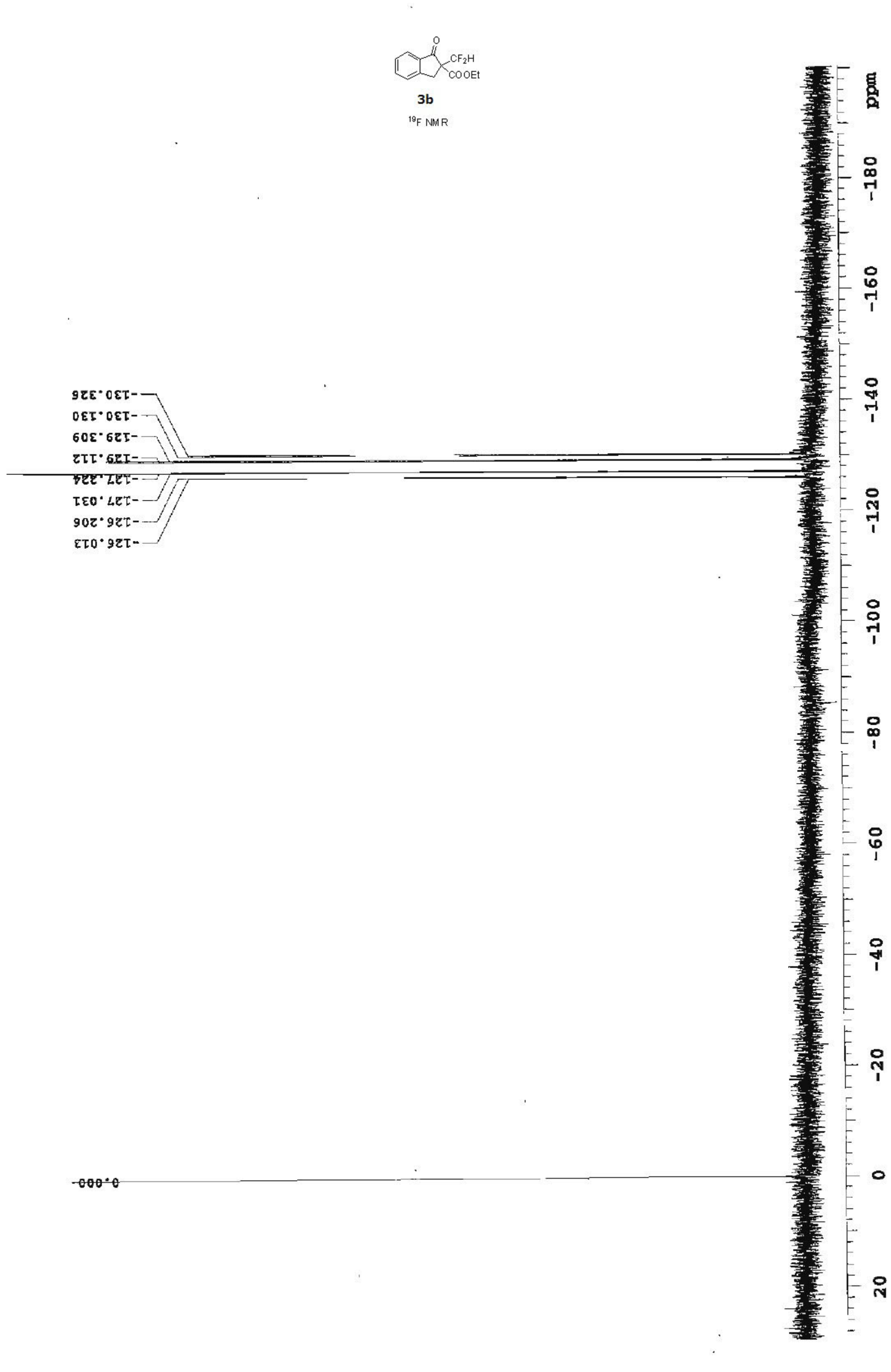
CF2H
COOEt
3b
19F NMR
-130.326
-130.130
-129.309
-129.112
-127.224
-127.031
-126.206
-126.013
0.000
ppm

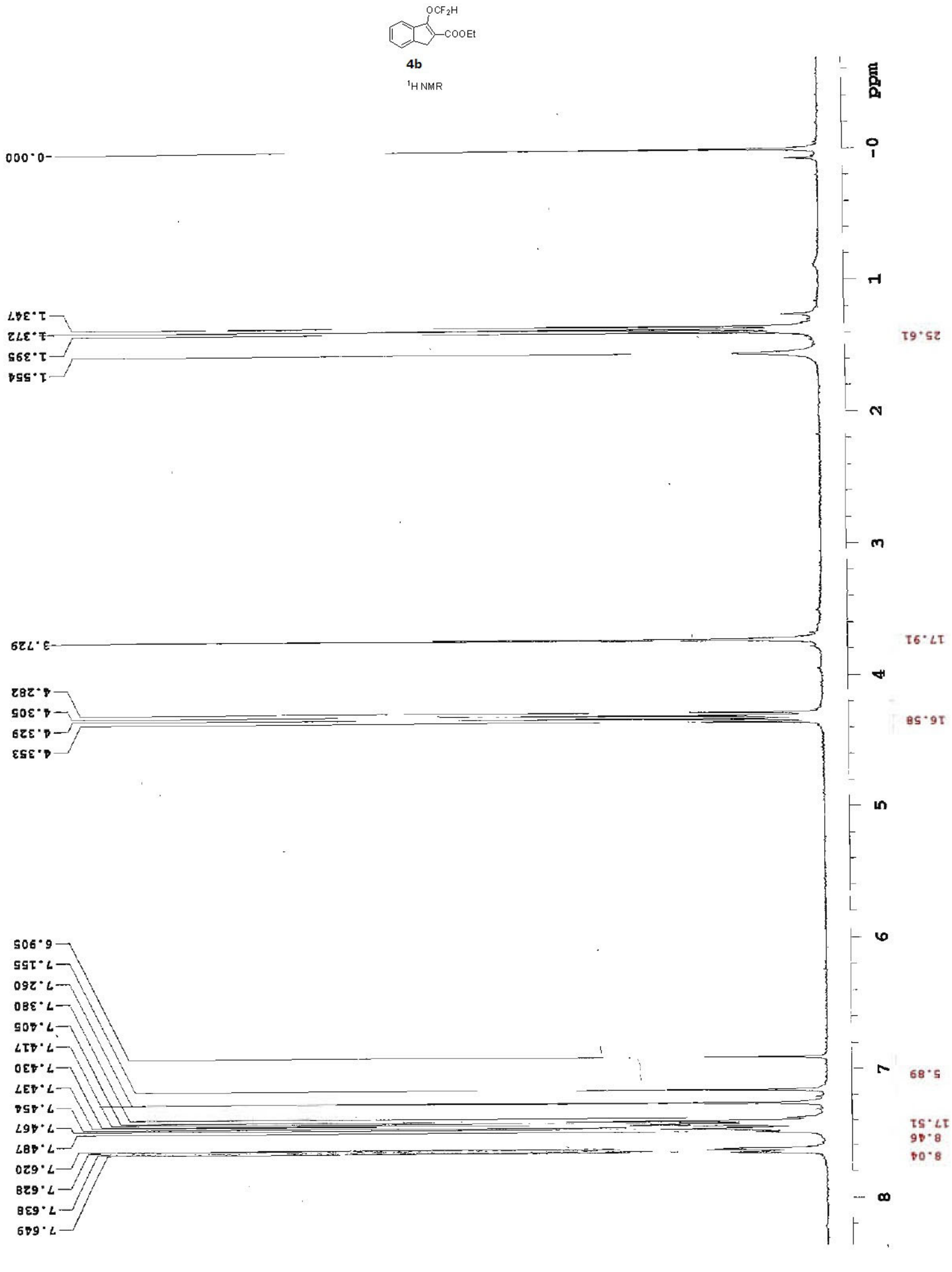
OCF2H
COOEt
4b
1H NMR
-0.000
1.347
1.372
1.395
1.554
3.729
4.282
4.305
4.329
4.353
6.905
7.155
7.260
7.380
7.405
7.417
7.430
7.437
7.454
7.467
7.487
7.620
7.628
7.638
7.649
25.61
17.91
16.58
5.89
17.51
8.46
8.04
ppm
0
1
2
3
4
5
6
7
8

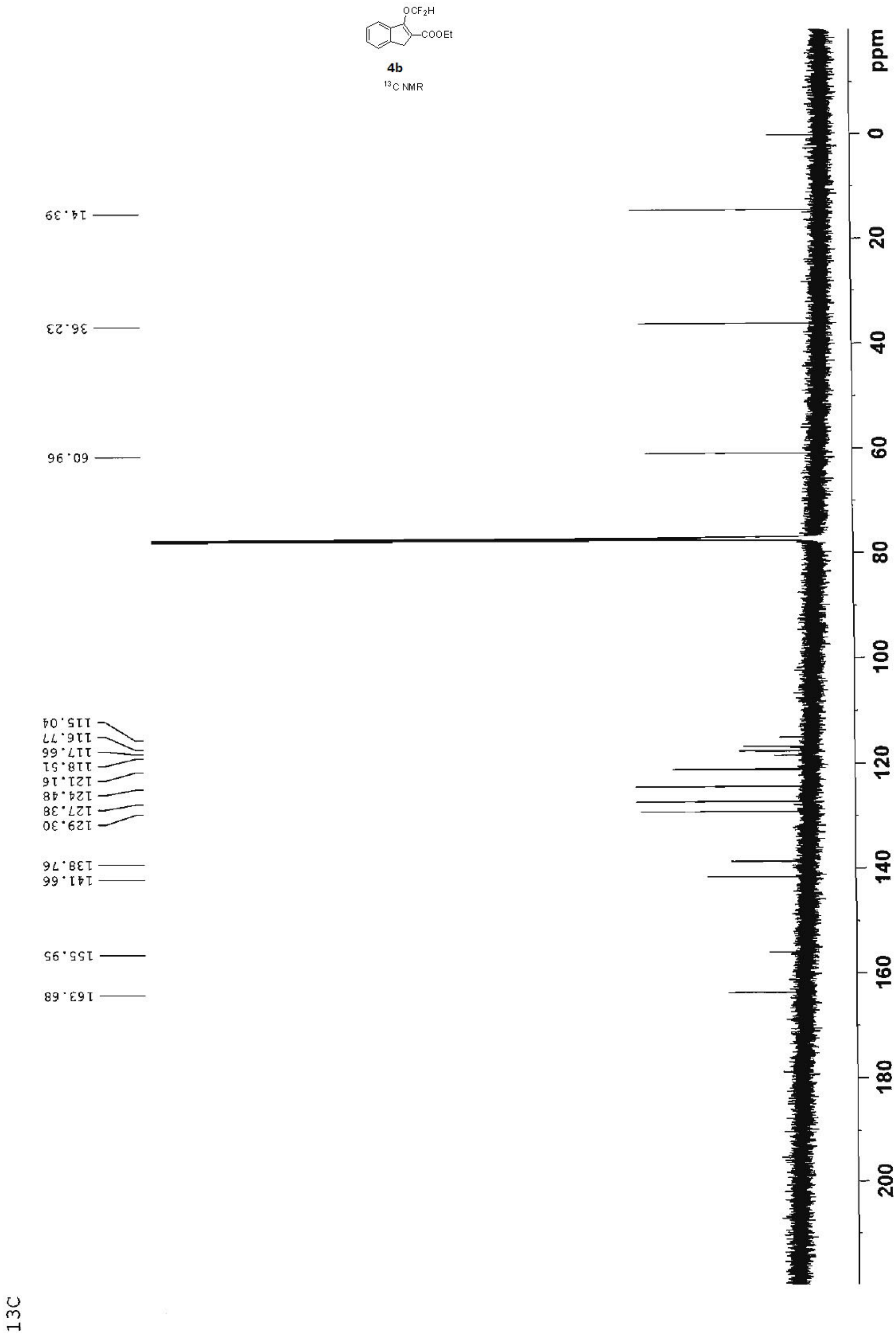
OCF2H
COOEt
4b
13C NMR
14.39
36.23
60.96
115.04
116.77
117.66
118.51
121.16
124.48
127.38
129.30
138.76
141.66
155.95
163.68
ppm
13C

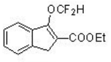
OCF2H
COOEt

4b

^{19}F NMR

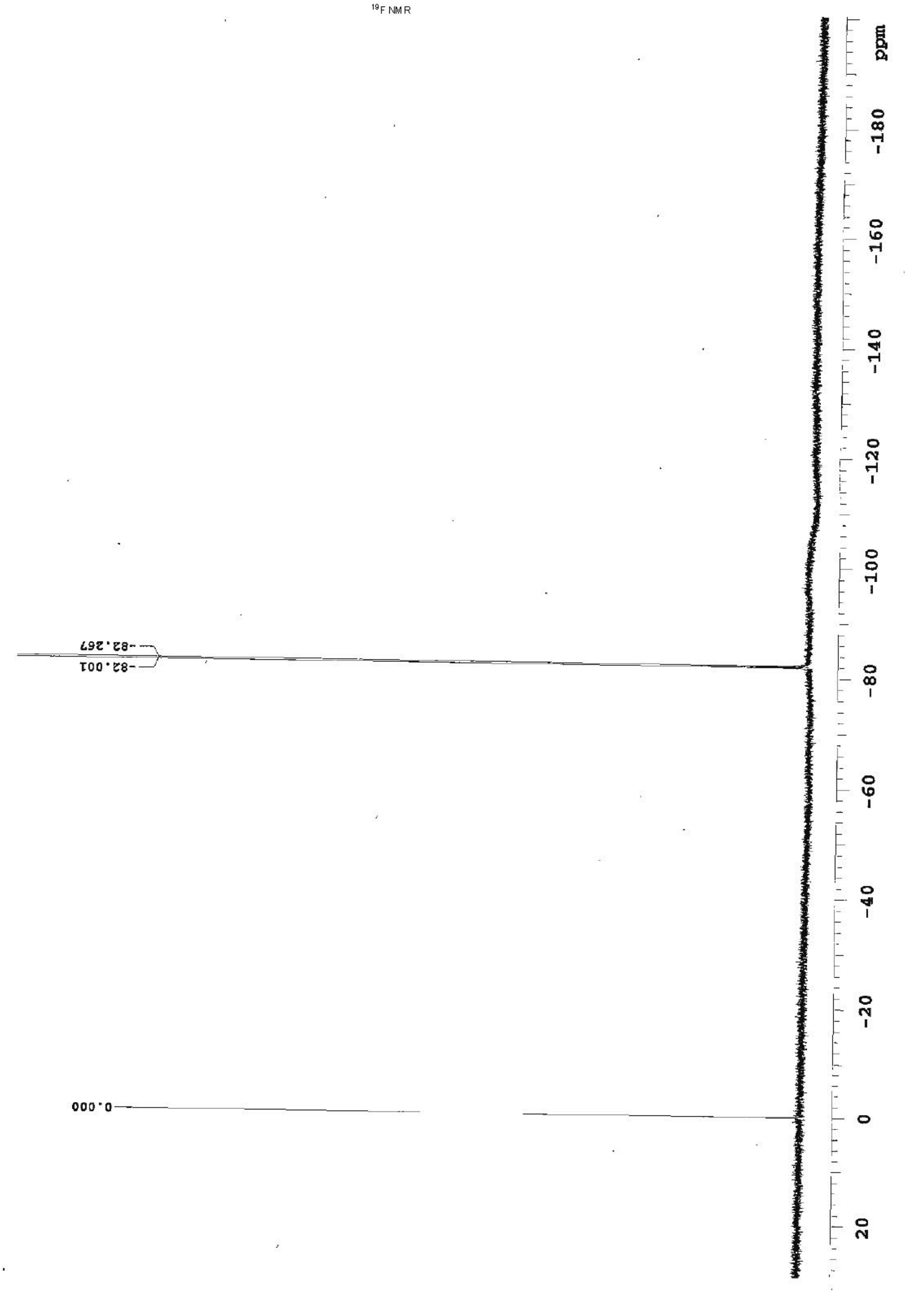
-82.267
-82.001
0.000
ppm
-180
-160
-140
-120
-100
-80
-60
-40
-20
0
20

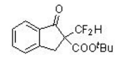

3c

^1H NMR

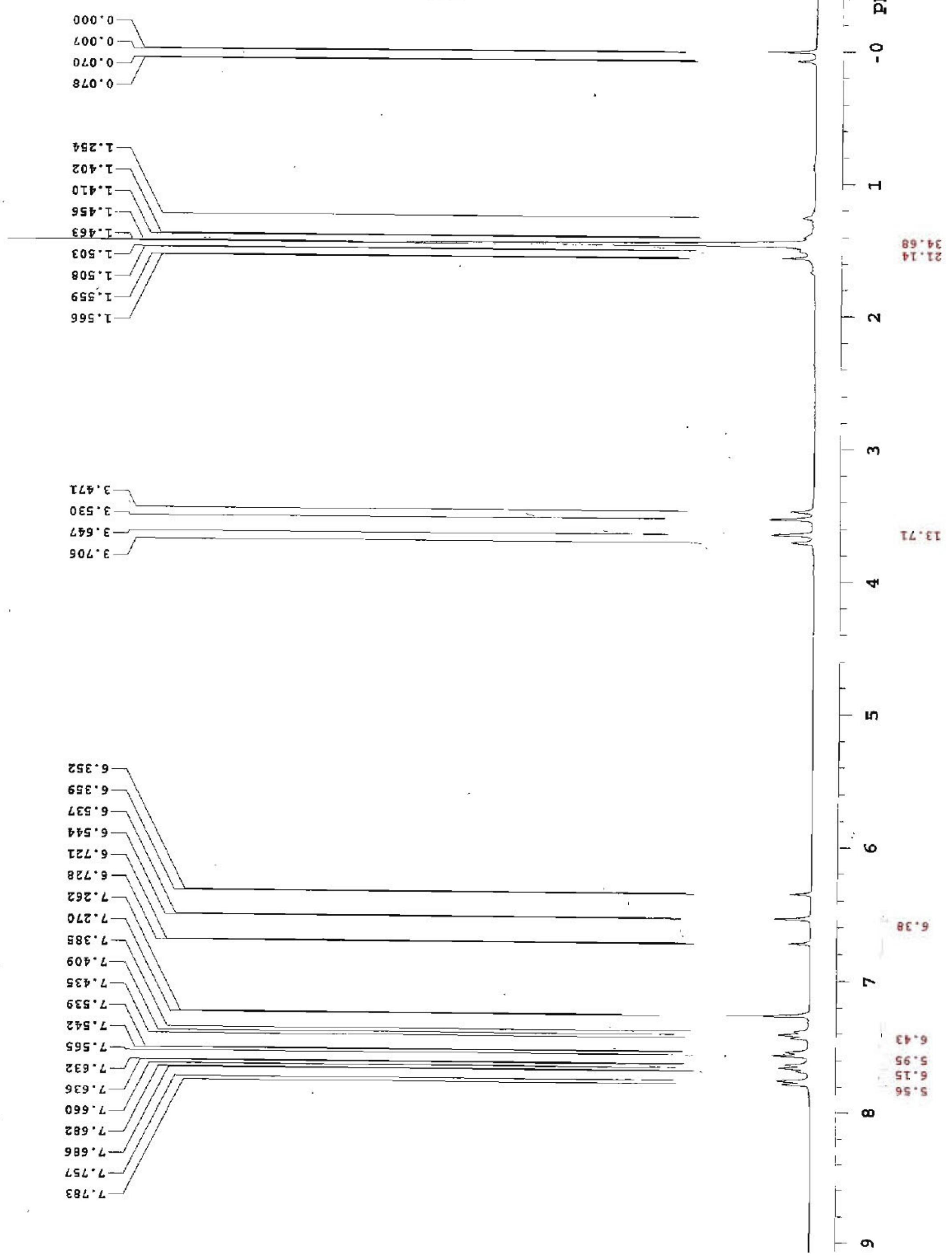

0.000
0.007
0.070
0.078
1.254
1.402
1.410
1.456
1.463
1.503
1.508
1.559
1.566
3.471
3.530
3.647
3.706
6.352
6.359
6.537
6.544
6.721
6.728
7.262
7.270
7.388
7.409
7.435
7.539
7.542
7.565
7.632
7.636
7.660
7.682
7.686
7.757
7.783
34.68
21.14
13.71
6.38
6.43
5.95
6.15
5.56
ppm
-0
1
2
3
4
5
6
7
8
9

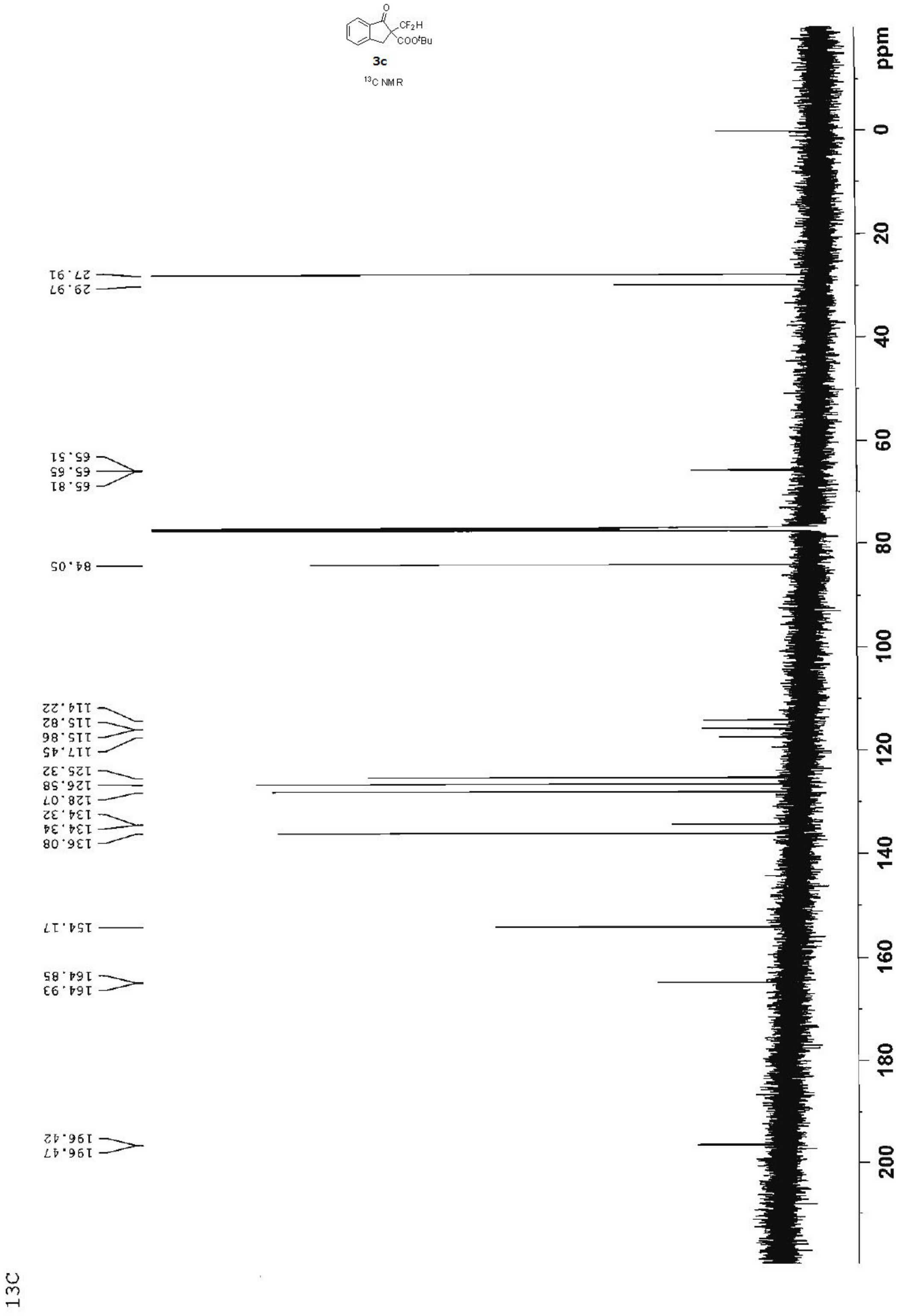
3c
13C NMR
ppm
13C
27.91
29.97
65.51
65.65
65.81
84.05
114.22
115.82
115.86
117.45
125.32
126.58
128.07
134.32
134.34
136.08
154.17
164.85
164.93
196.42
196.47

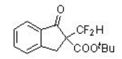

3c

^{19}F NMR

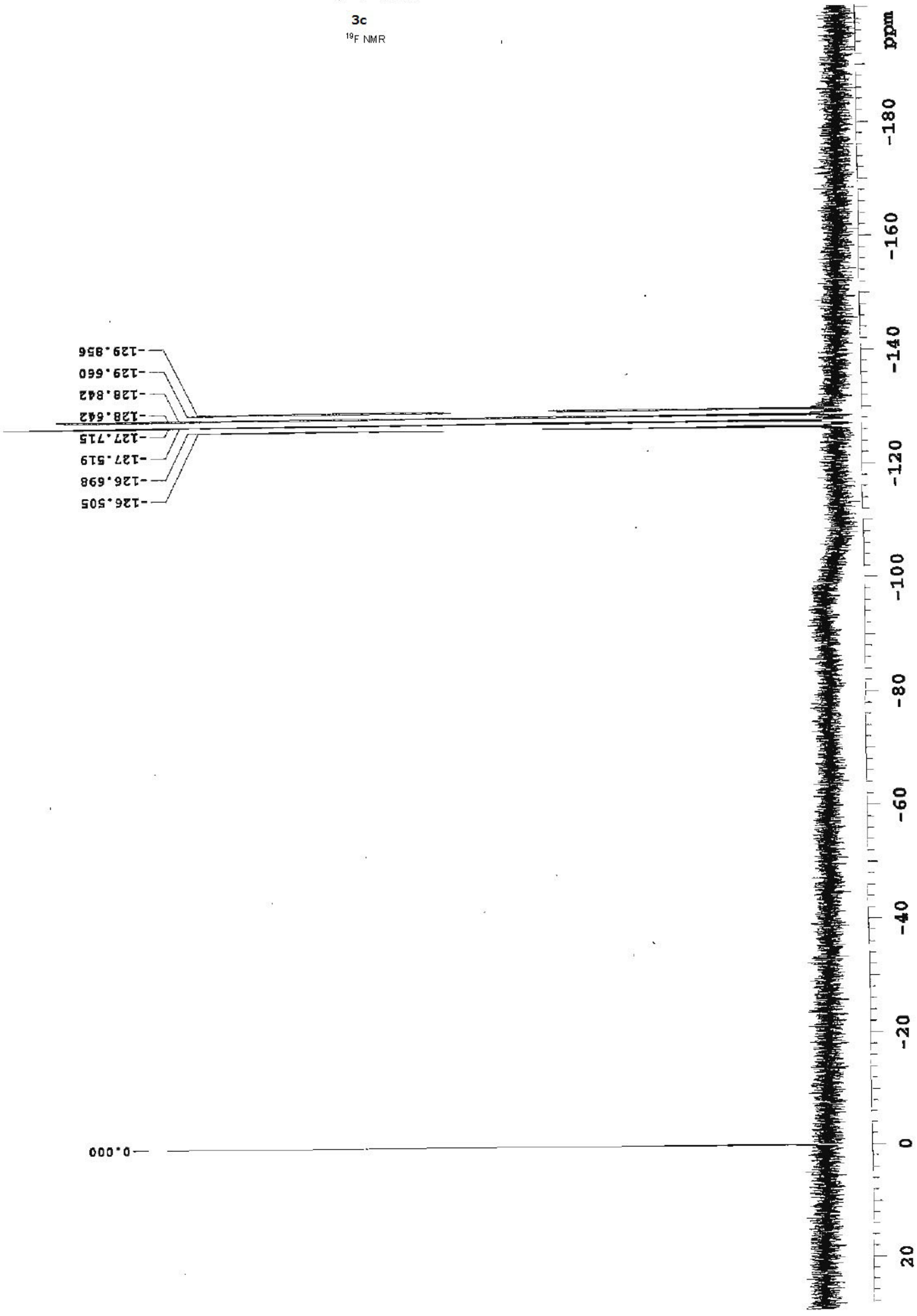
-129.856
-129.660
-128.842
-128.642
-127.715
-127.519
-126.698
-126.505
0.000
ppm

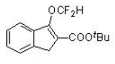
OCF2H
COOtBu

4c

^{1}H NMR

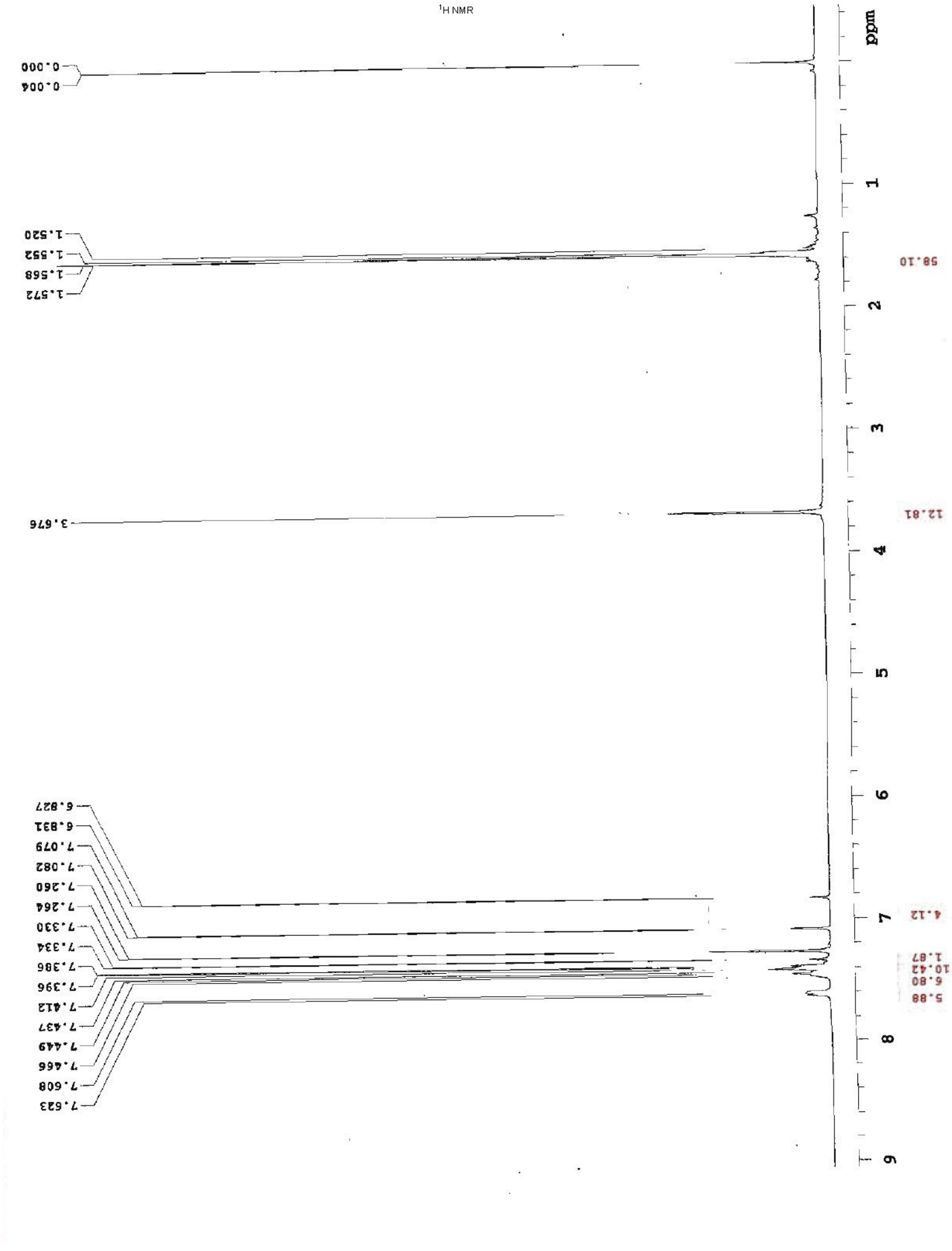
0.000
0.004
1.520
1.552
1.568
1.572
3.676
6.827
6.831
7.079
7.082
7.260
7.264
7.330
7.334
7.386
7.396
7.413
7.437
7.449
7.466
7.608
7.623
58.10
12.81
4.12
1.87
10.42
6.80
5.88
ppm
1
2
3
4
5
6
7
8
9

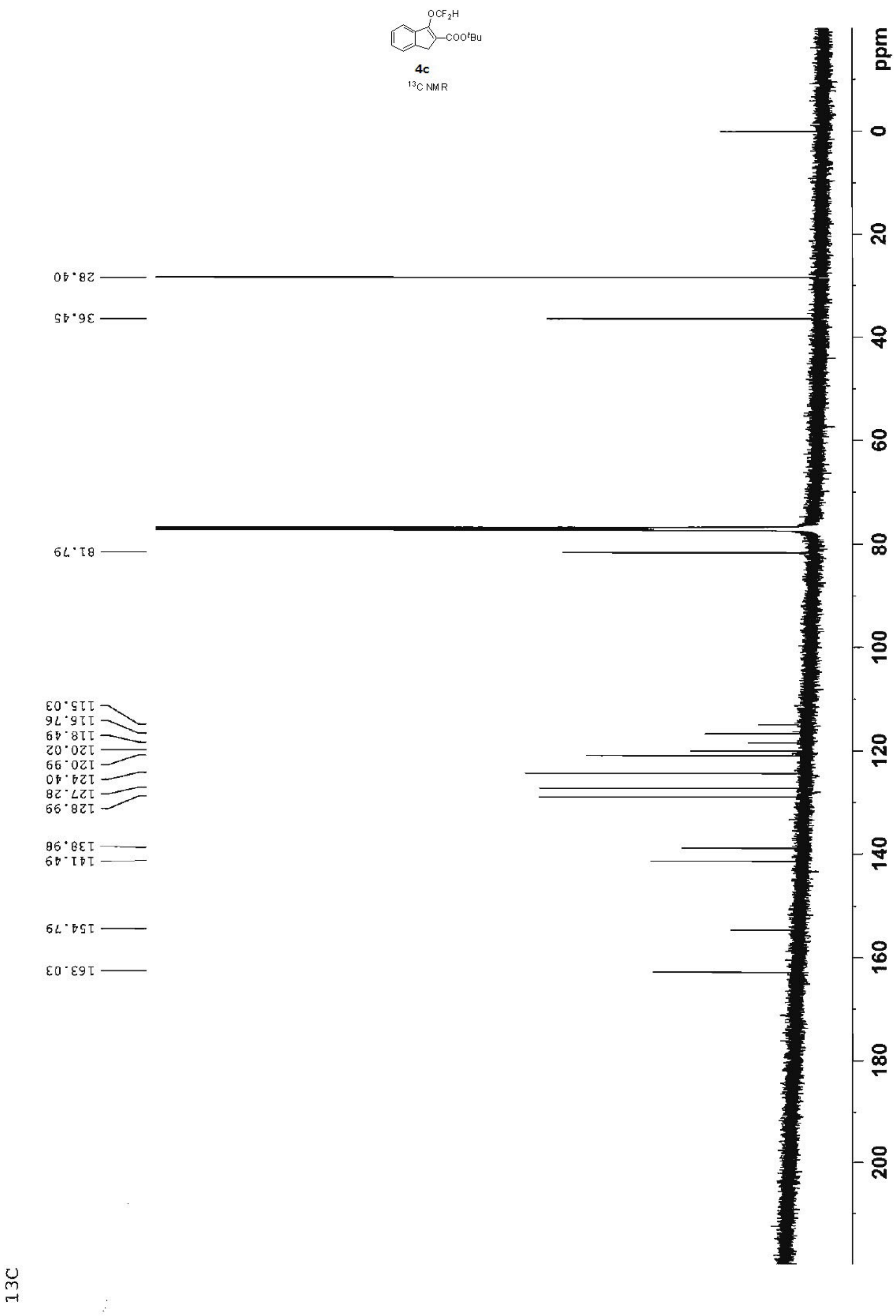
OCF2H
COOtBu
4c
13C NMR
28.40
36.45
81.79
115.03
116.76
118.49
120.02
120.99
124.40
127.28
128.99
138.98
141.49
154.79
163.03
ppm
0
20
40
60
80
100
120
140
160
180
200
13C

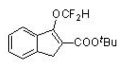
OCF2H
COOtBu

4c

19F NMR

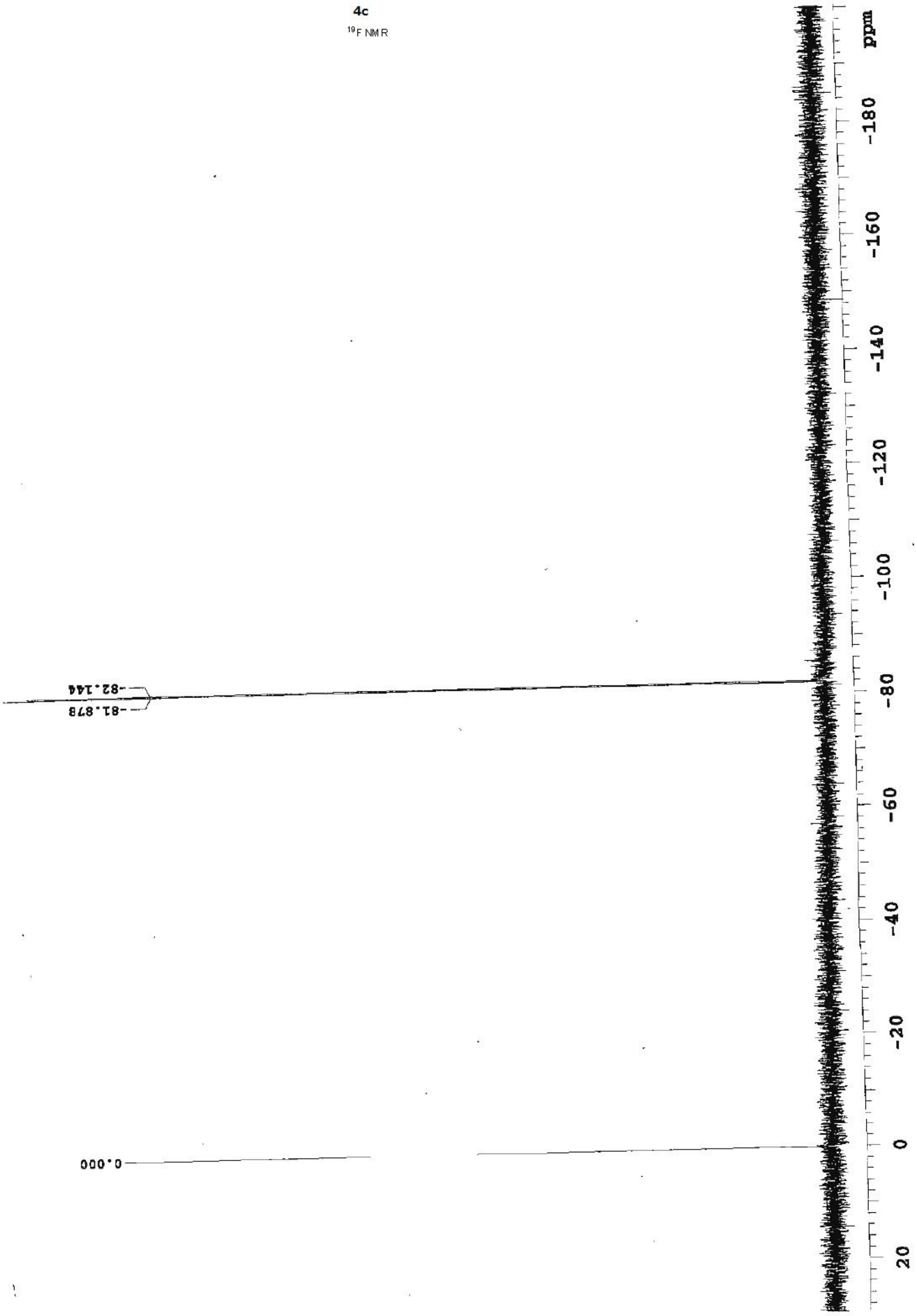
-82.144
-81.878
0.000
ppm
-180
-160
-140
-120
-100
-80
-60
-40
-20
0
20

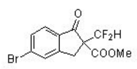
O
CF2H
COOMe
Br

3d

^{1}H NMR

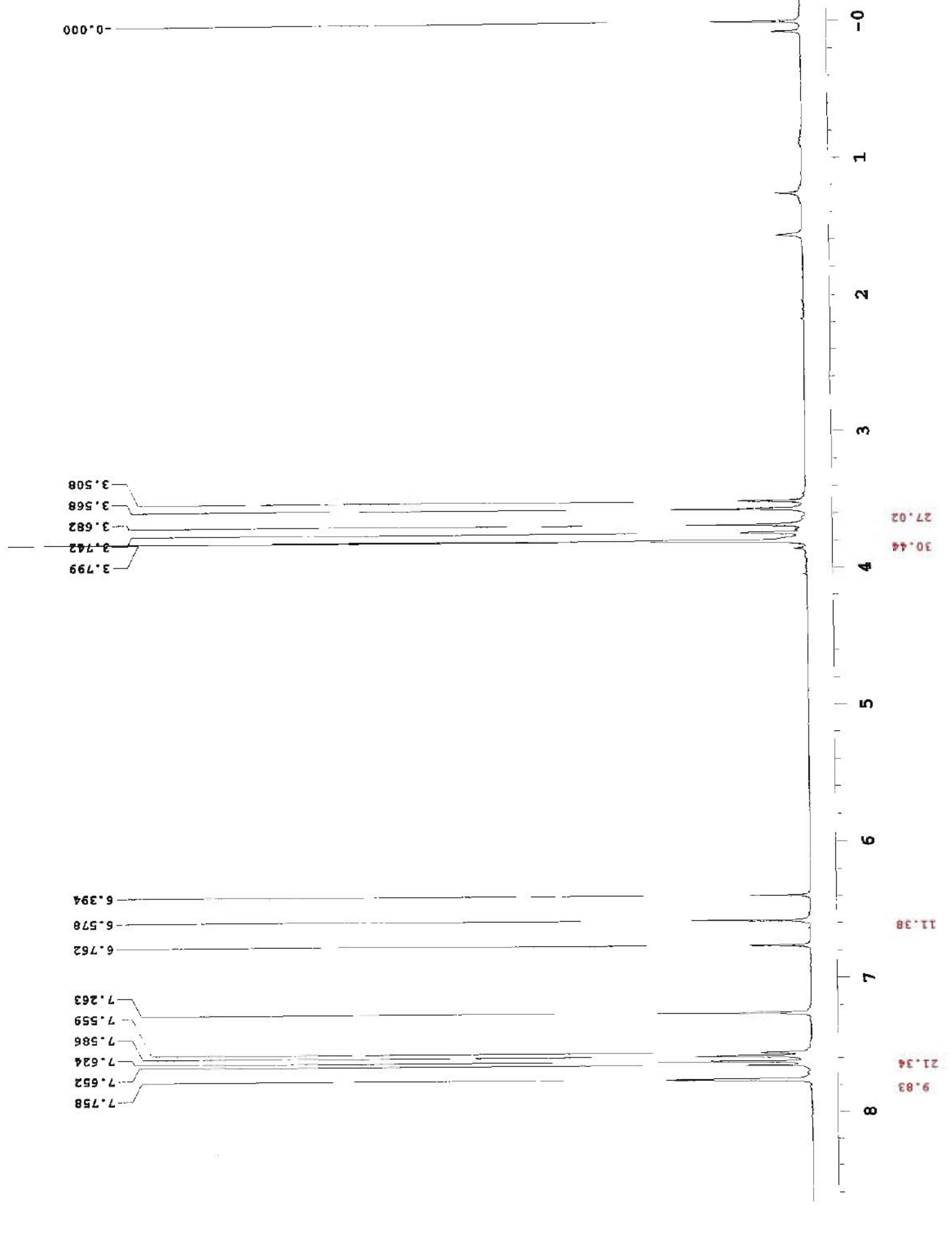
-0.000
3.508
3.568
3.682
3.742
3.799
6.394
6.578
6.762
7.263
7.559
7.586
7.634
7.652
7.758
27.02
30.44
11.38
21.34
9.83
ppm

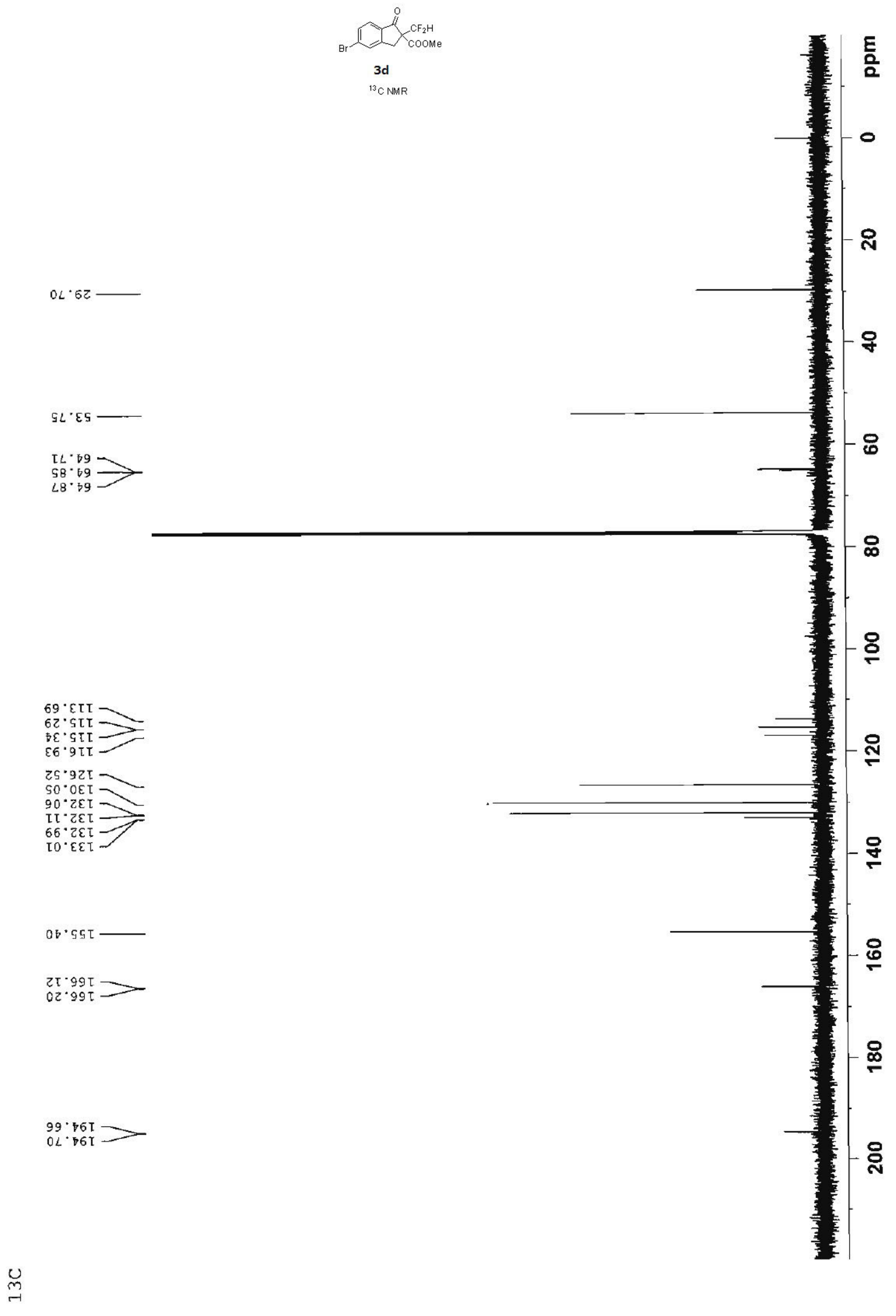
CF2H
COOMe
Br
O
3d
13C NMR
ppm
29.70
53.75
64.71
64.85
64.87
113.69
115.29
115.34
116.93
126.52
130.05
132.06
132.11
132.99
133.01
155.40
166.12
166.20
194.66
194.70
0
20
40
60
80
100
120
140
160
180
200
13C

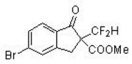
O
CF2H
COOMe
Br

3d

^{19}F NMR

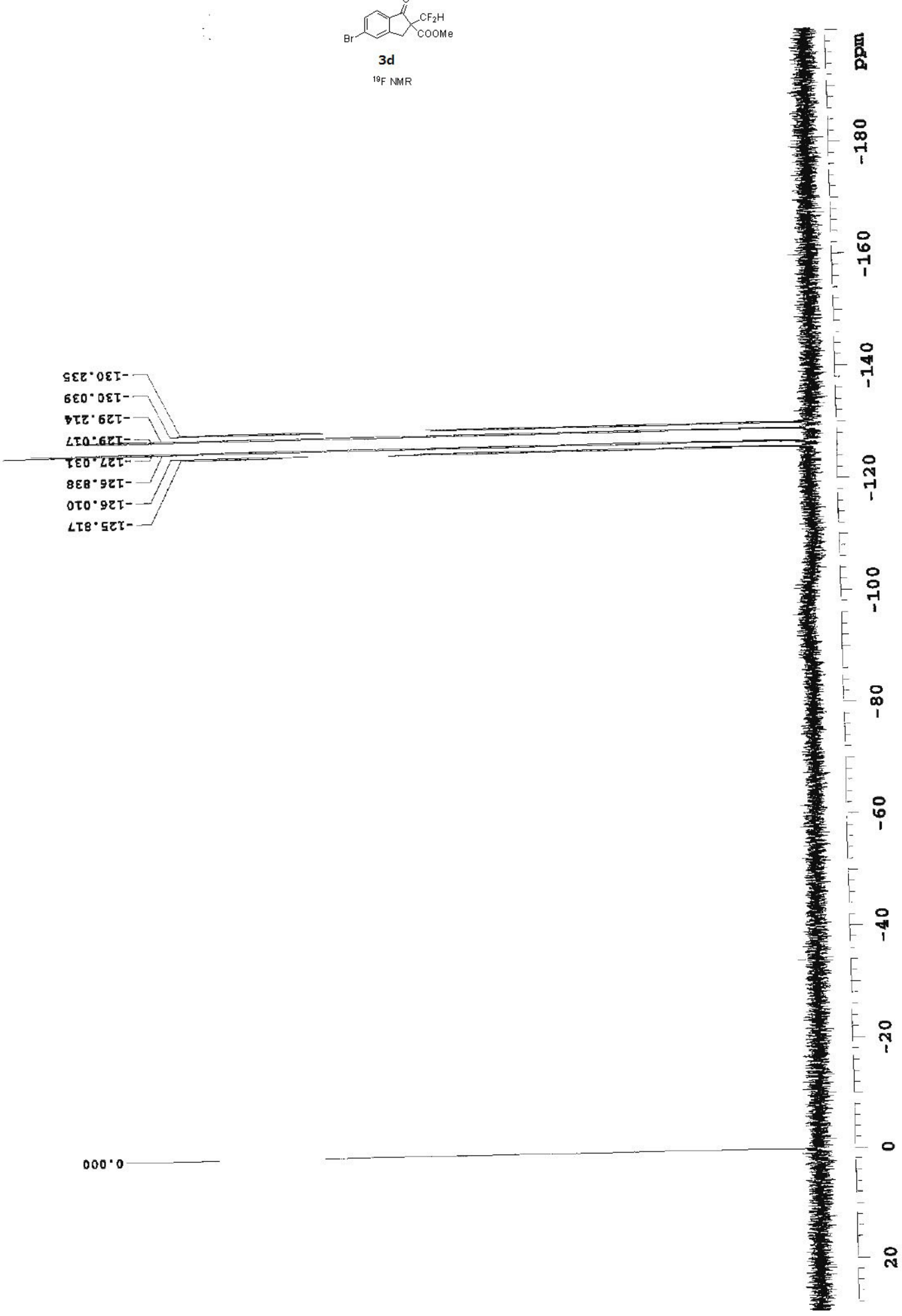
-130.235
-130.039
-129.214
-129.017
-127.031
-126.838
-126.010
-125.817
0.000
ppm
-180
-160
-140
-120
-100
-80
-60
-40
-20
0
20

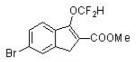
OCF2H
COOMe
Br

4d

^1H NMR

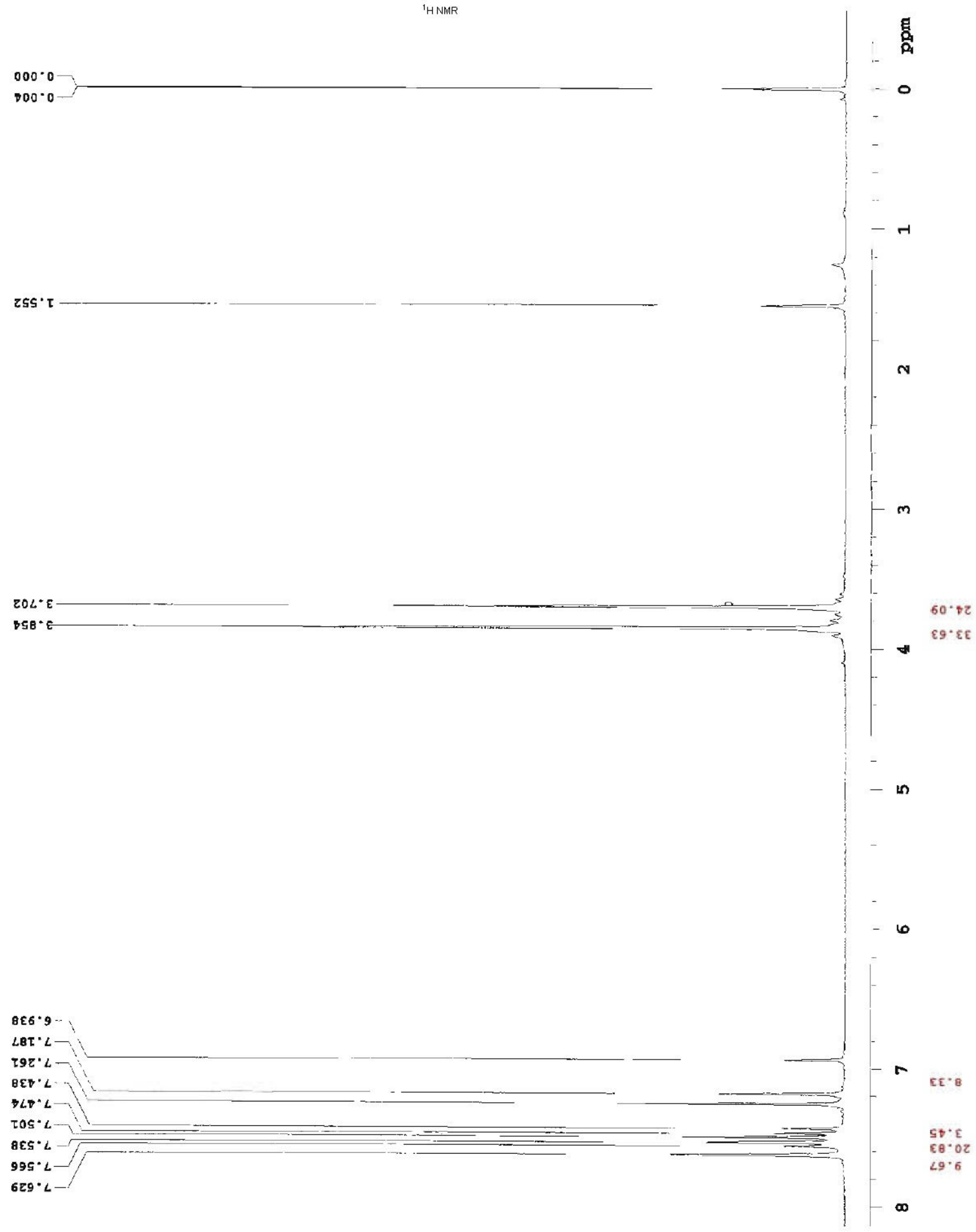
0.000
0.004
1.552
3.702
3.854
6.938
7.187
7.261
7.438
7.474
7.501
7.538
7.566
7.629
24.09
33.63
8.33
3.45
20.83
9.67
ppm
0
1
2
3
4
5
6
7
8

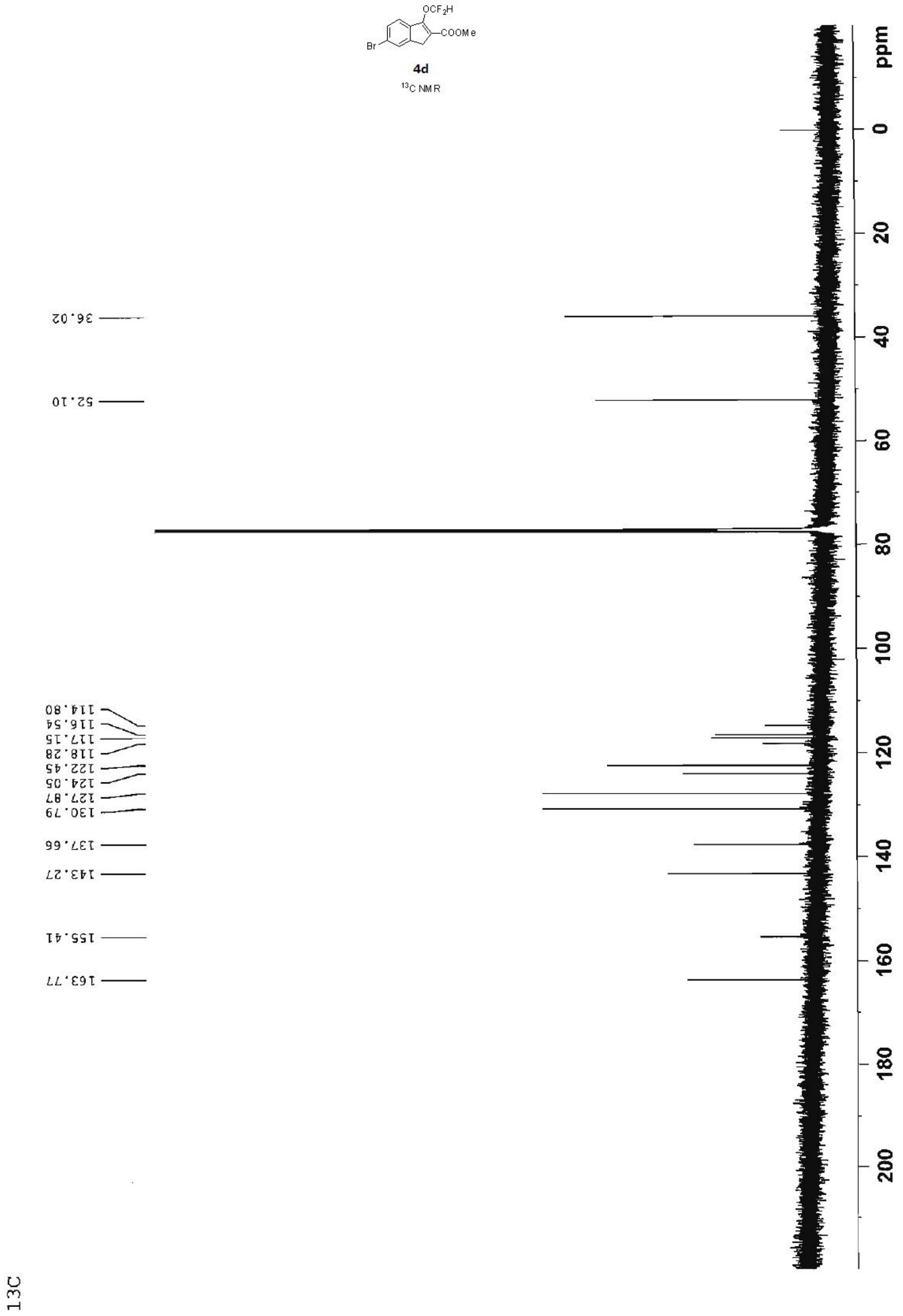
Br
4d
^{13}C NMR
36.02
52.10
114.80
116.54
117.15
118.28
122.45
124.05
127.87
130.79
137.66
143.27
155.41
163.77
ppm
13C

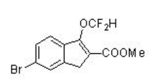
OCF2H
COOMe
Br

4d

^{19}F NMR

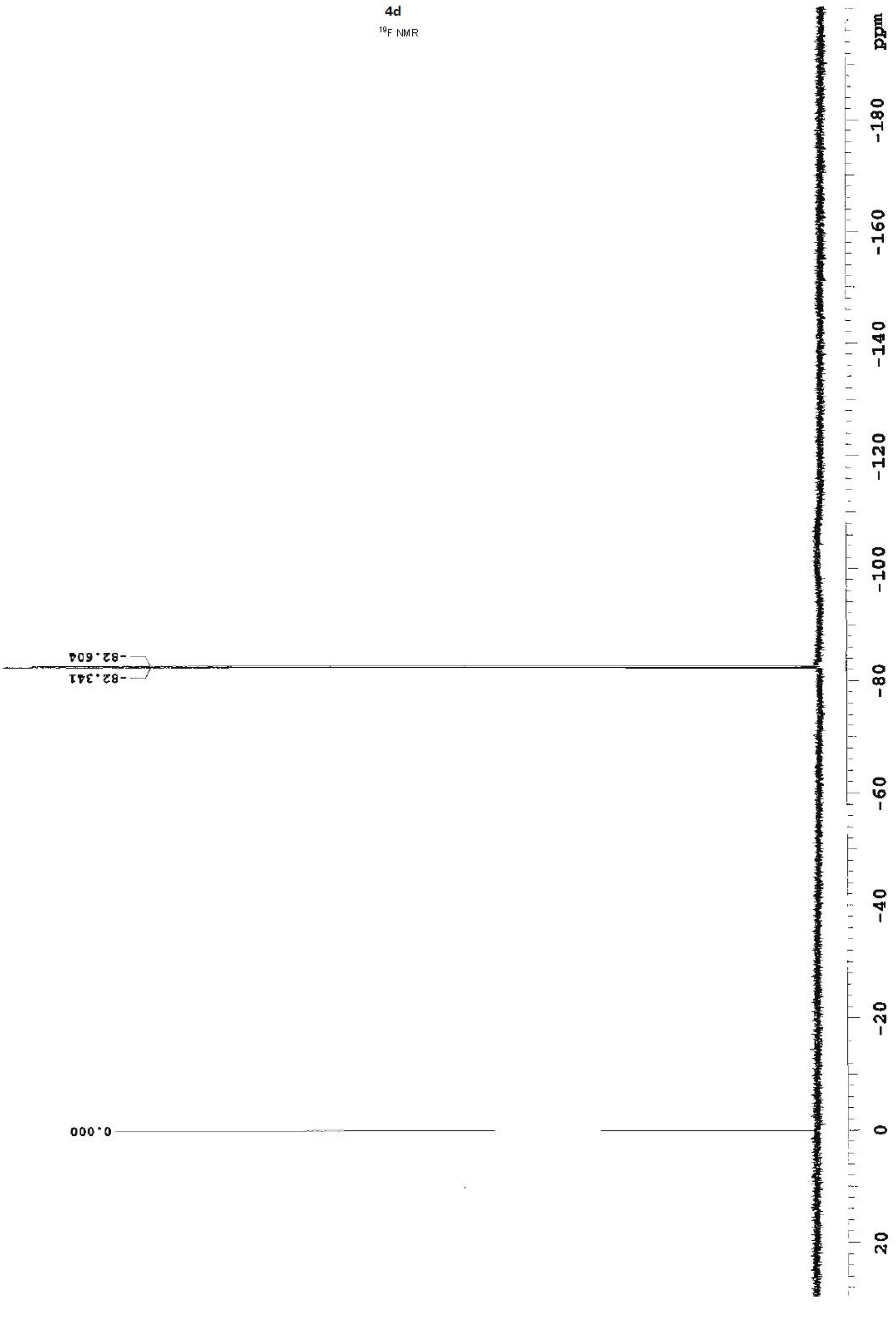
-82.604
-82.341
0.000
ppm
-180
-160
-140
-120
-100
-80
-60
-40
-20
0
20

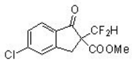
CF2H
COOMe
Cl
O

3e

^{1}H NMR

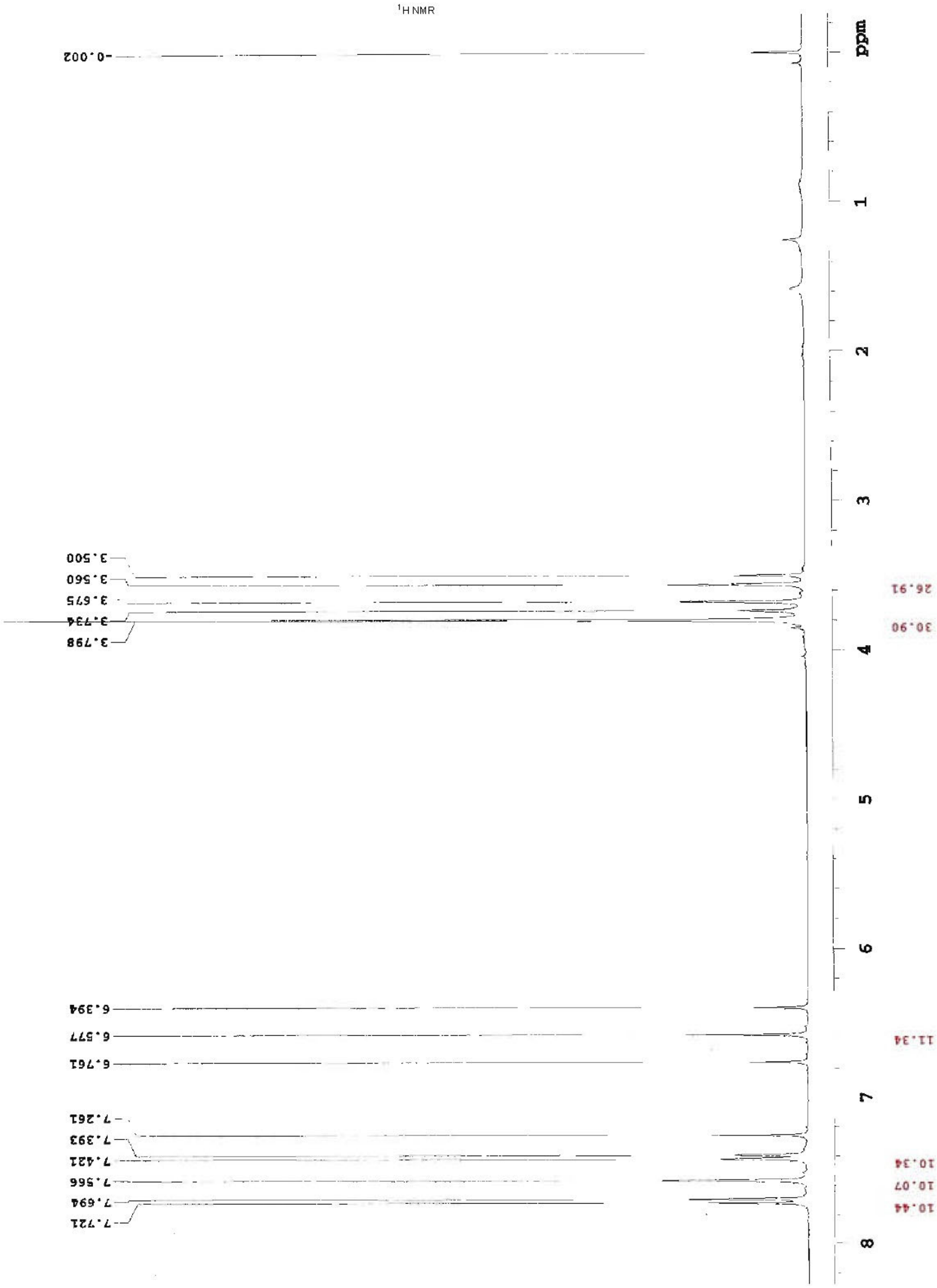
-0.002
3.500
3.560
3.675
3.734
3.798
6.394
6.577
6.761
7.261
7.393
7.431
7.566
7.694
7.721
26.91
30.90
11.34
10.34
10.07
10.44
ppm
1
2
3
4
5
6
7
8

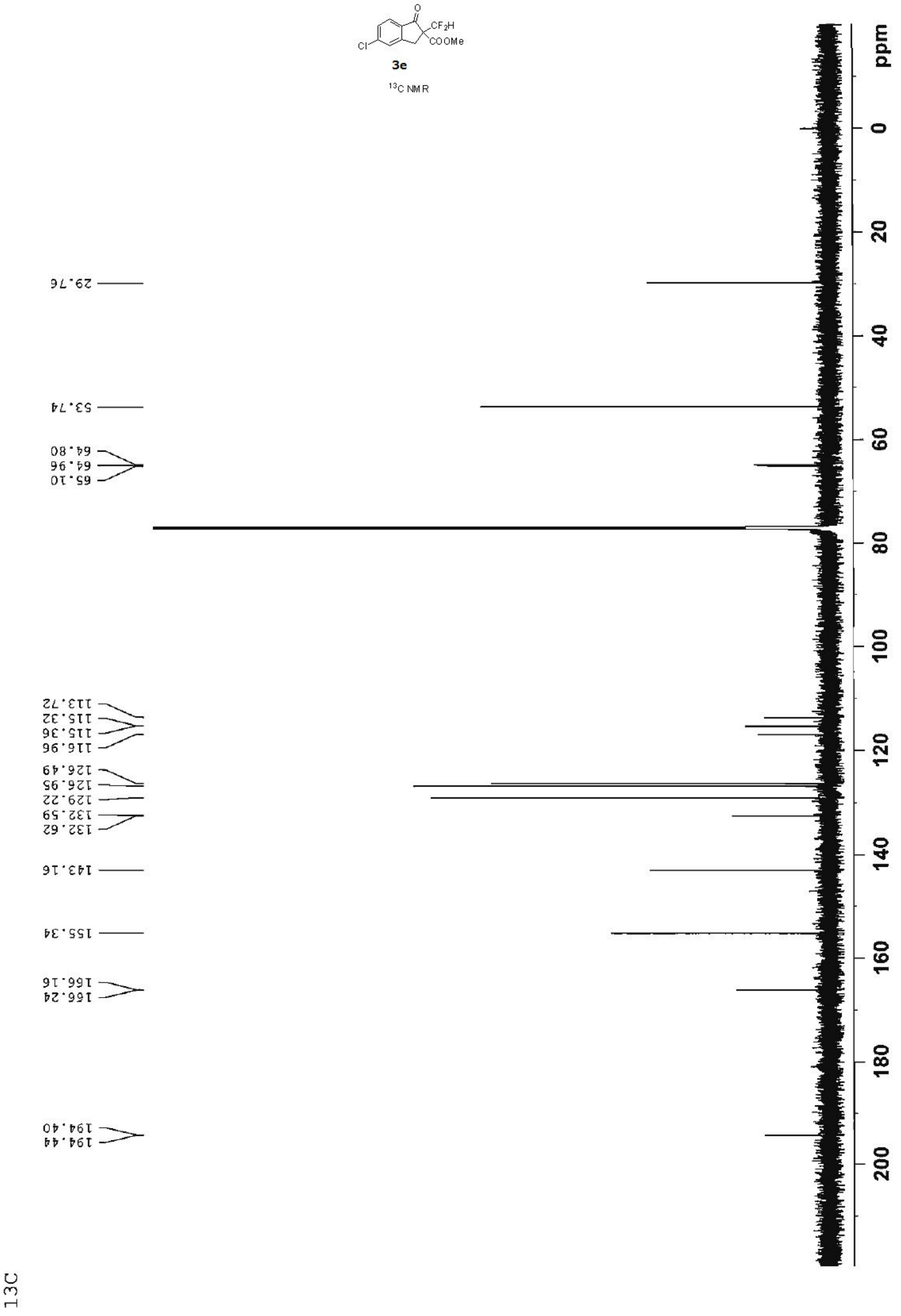
3e
13C NMR
13C
ppm
29.76
53.74
64.80
64.96
65.10
113.72
115.32
115.36
116.96
126.49
126.95
129.22
132.59
132.62
143.16
155.34
166.16
166.24
194.40
194.44

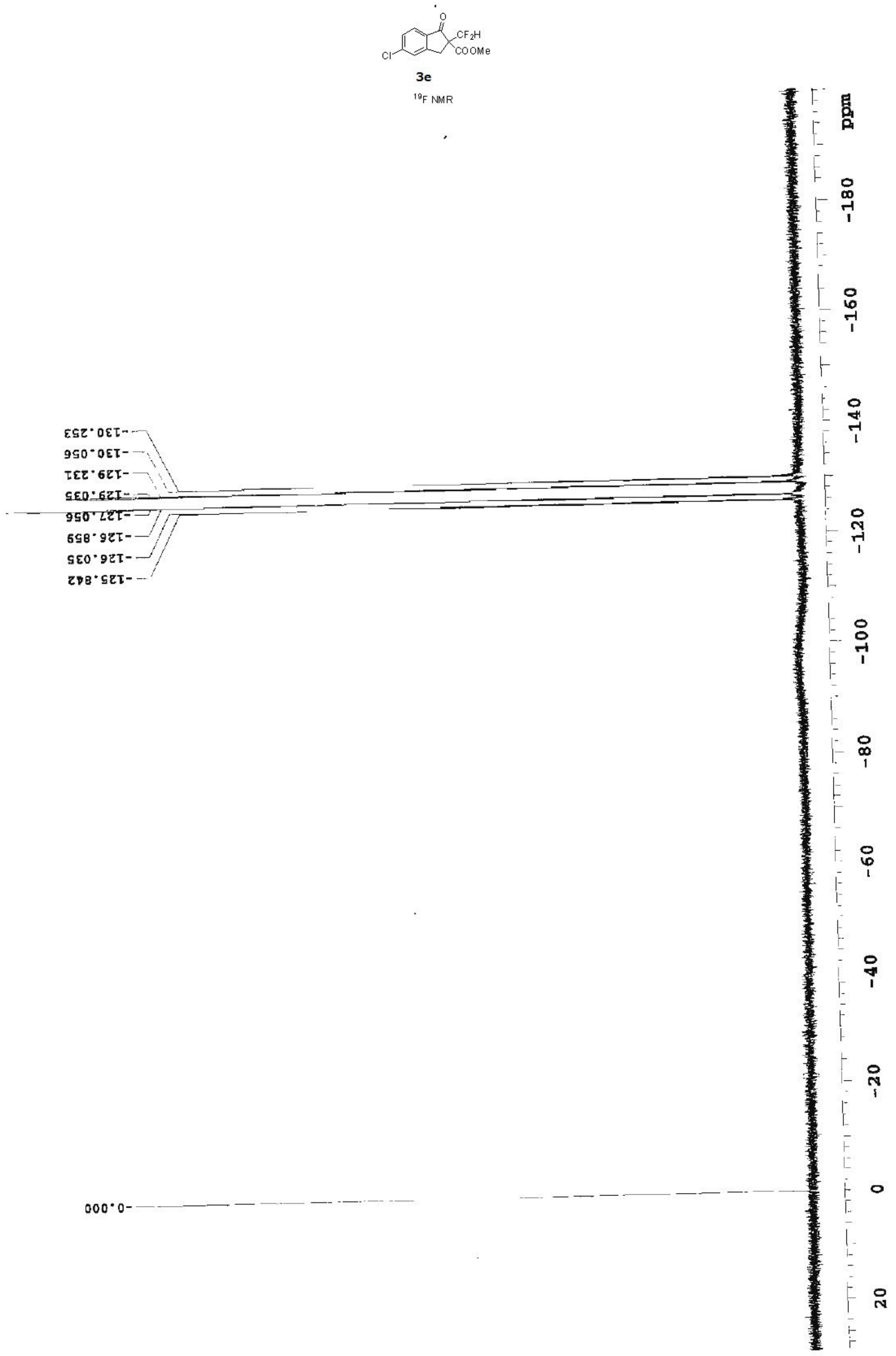
O
CF_2H
COOMe
Cl
3e
19F NMR
-130.253
-130.056
-129.231
-129.035
-127.056
-126.859
-126.035
-125.842
0.000
ppm
-180
-160
-140
-120
-100
-80
-60
-40
-20
0
20

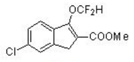
OCF2H
COOMe
Cl

4e

^{1}H NMR

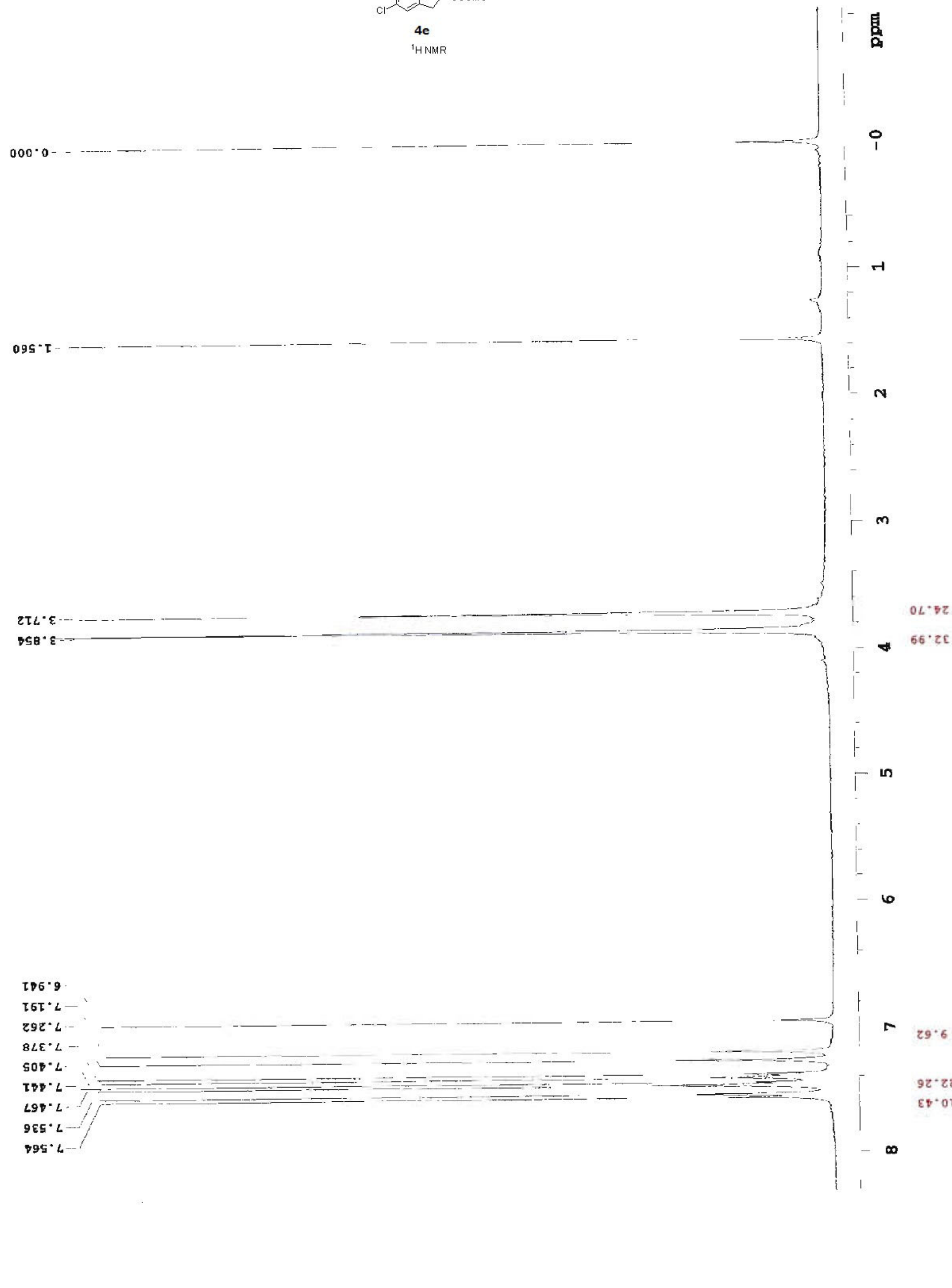
0.000
1.560
3.712
3.854
6.941
7.191
7.262
7.378
7.405
7.441
7.467
7.536
7.564
24.70
32.99
9.62
22.26
10.43
ppm
0
1
2
3
4
5
6
7
8

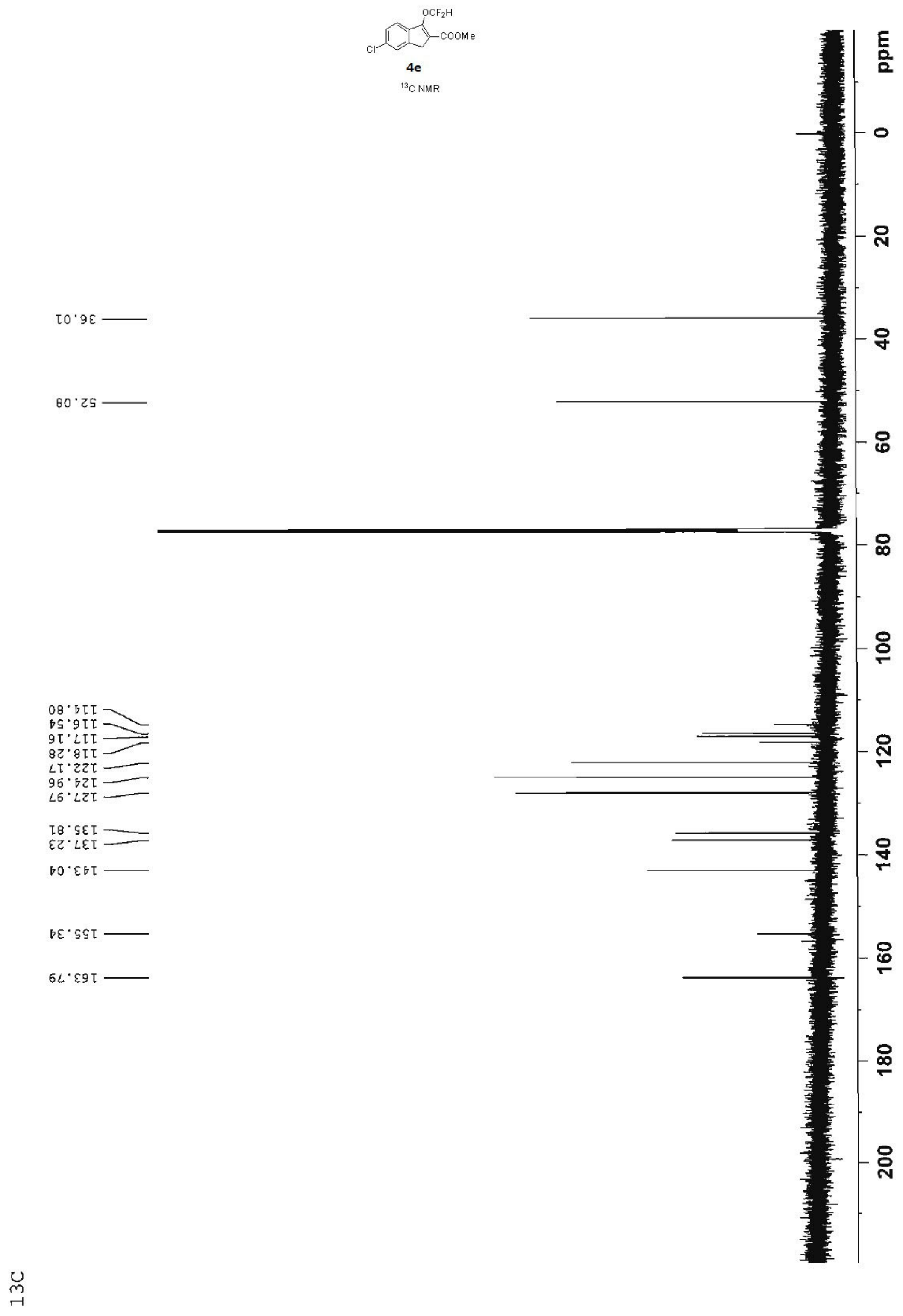
OCF2H
COOMe
Cl
4e
13C NMR
36.01
52.08
114.80
116.54
117.16
118.28
122.17
124.96
127.97
135.81
137.23
143.04
155.34
163.79
13C
ppm
0
20
40
60
80
100
120
140
160
180
200

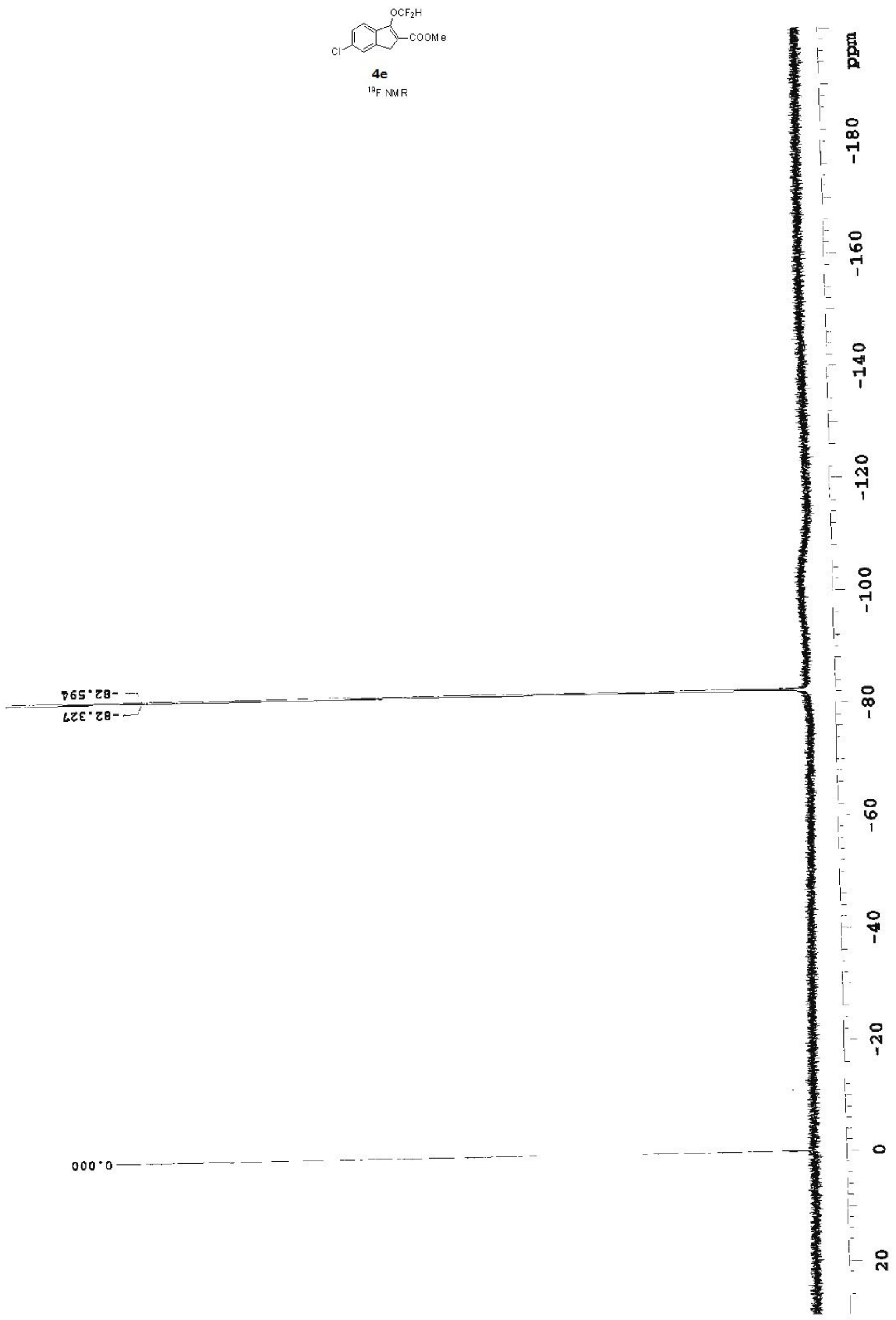
OCF2H
COOMe
Cl
4e
19F NMR
-82.594
-82.327
0.000
ppm
-180
-160
-140
-120
-100
-80
-60
-40
-20
0
20

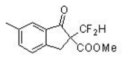
O
CF2H
COOMe

3f

^{1}H NMR

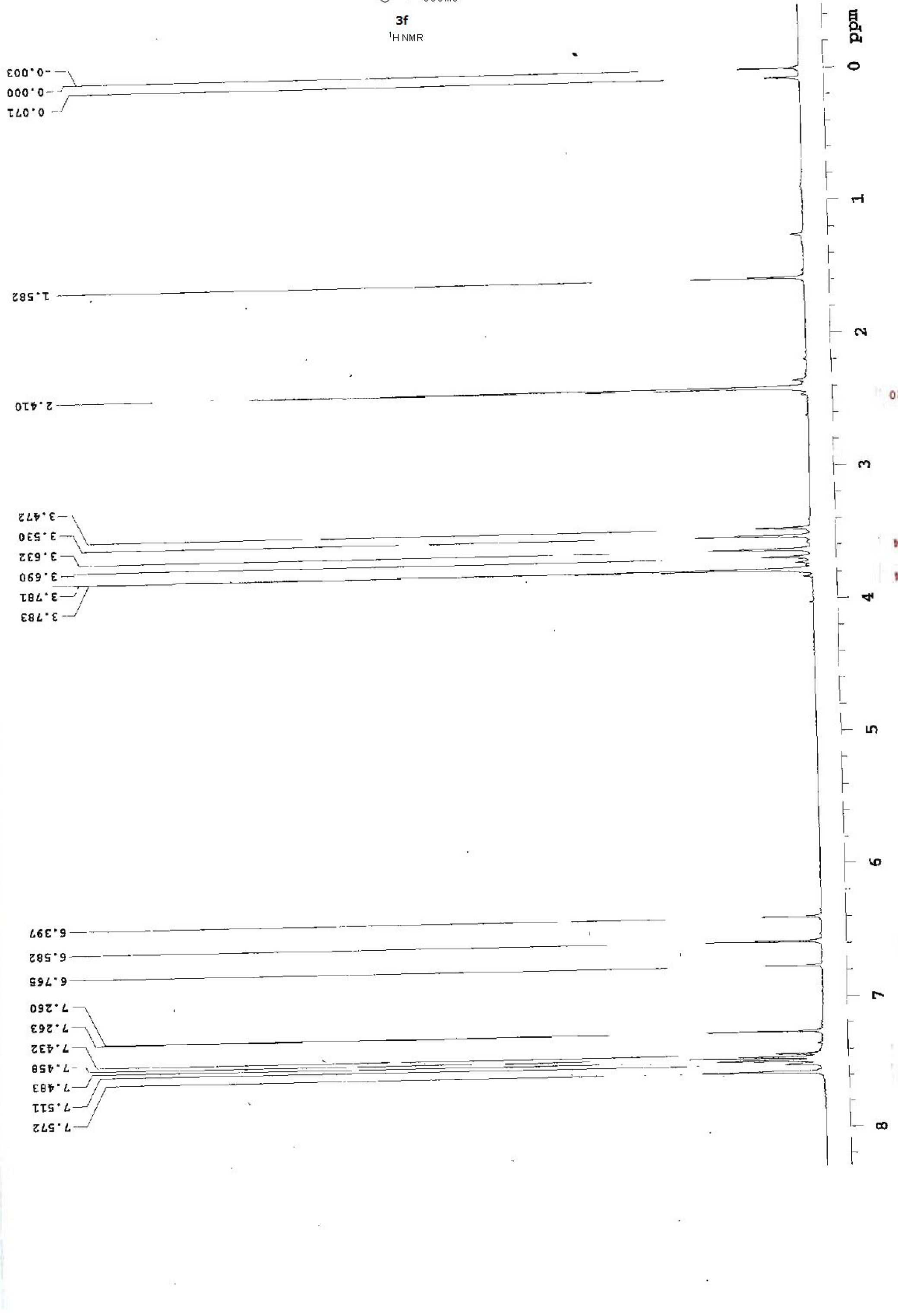
-0.003
0.000
0.071
1.582
2.410
3.472
3.530
3.632
3.690
3.781
3.783
6.397
6.582
6.765
7.260
7.263
7.432
7.458
7.483
7.511
7.572
24.20
19.74
23.94
8.12
16.51
7.49
ppm
0
1
2
3
4
5
6
7
8

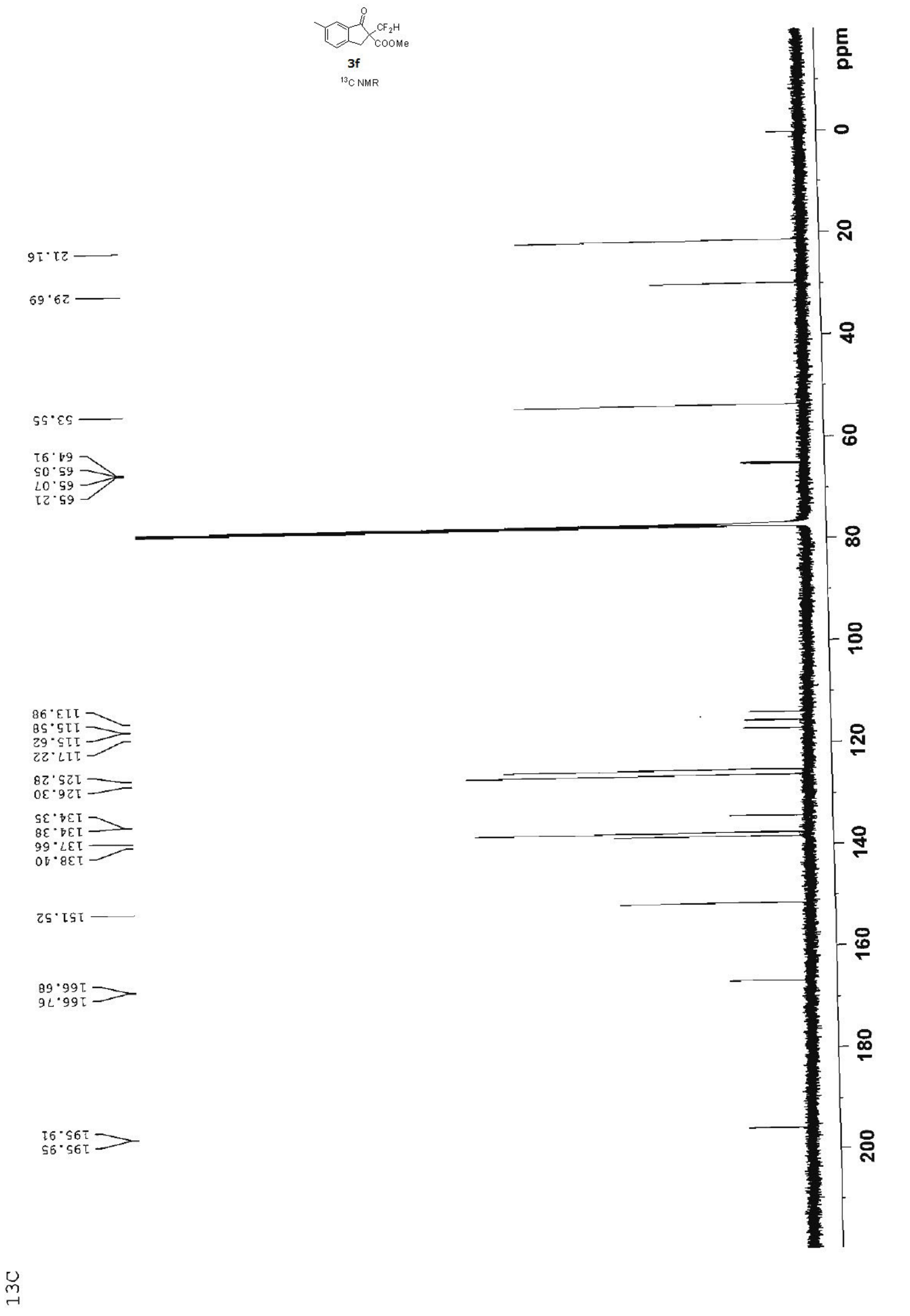
CF2H
COOMe
3f
13C NMR
21.16
29.69
53.55
64.91
65.05
65.07
65.21
113.98
115.58
115.62
117.22
125.28
126.30
134.35
134.38
137.66
138.40
151.52
166.68
166.76
195.91
195.95
ppm
0
20
40
60
80
100
120
140
160
180
200
13C

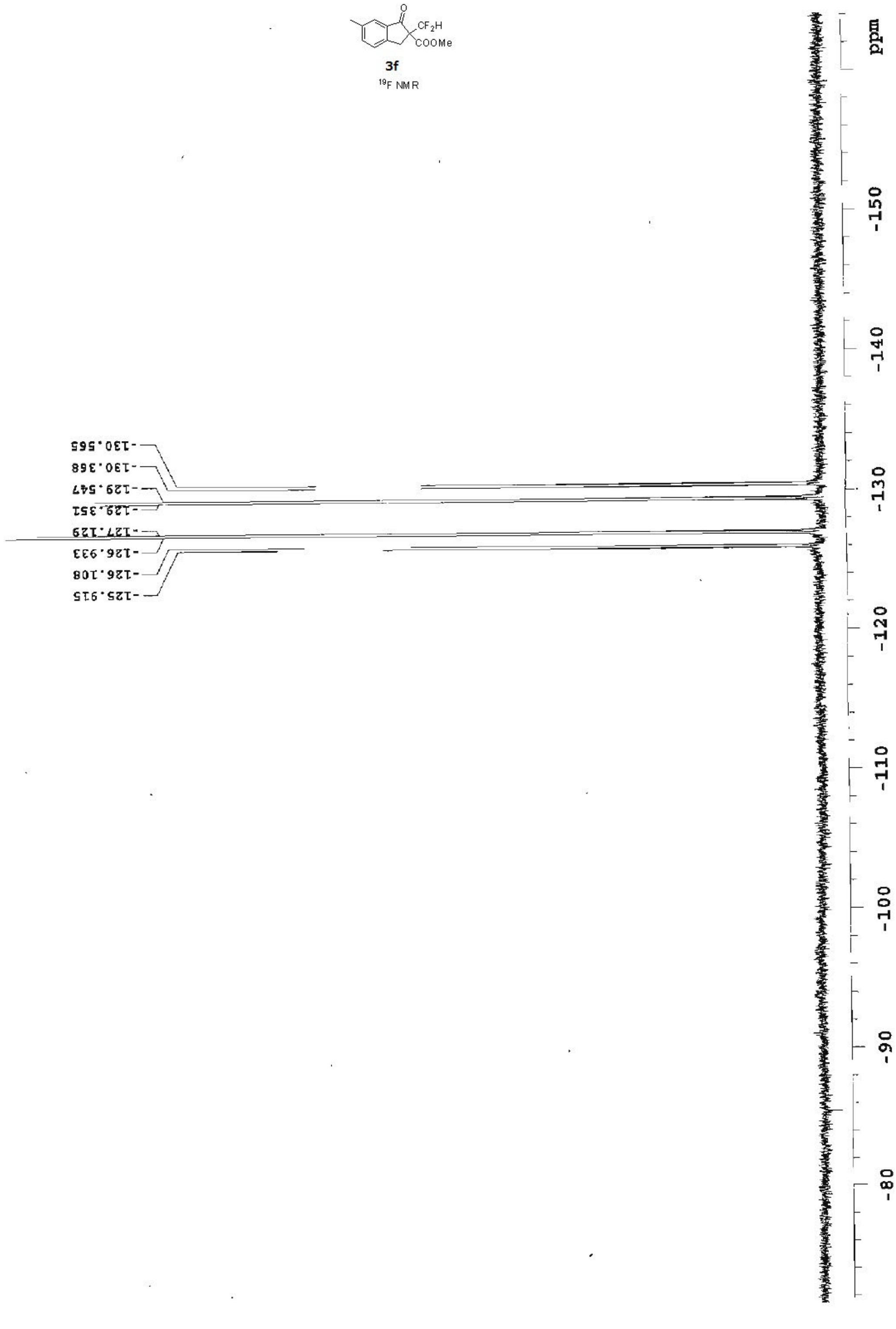
O
CF2H
COOMe
3f
19F NMR
-130.565
-130.368
-129.547
-129.351
-127.129
-126.933
-126.108
-125.915
ppm
-150
-140
-130
-120
-110
-100
-90
-80

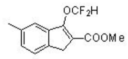
OCF$_2$H
COOMe

4f

^{1}H NMR

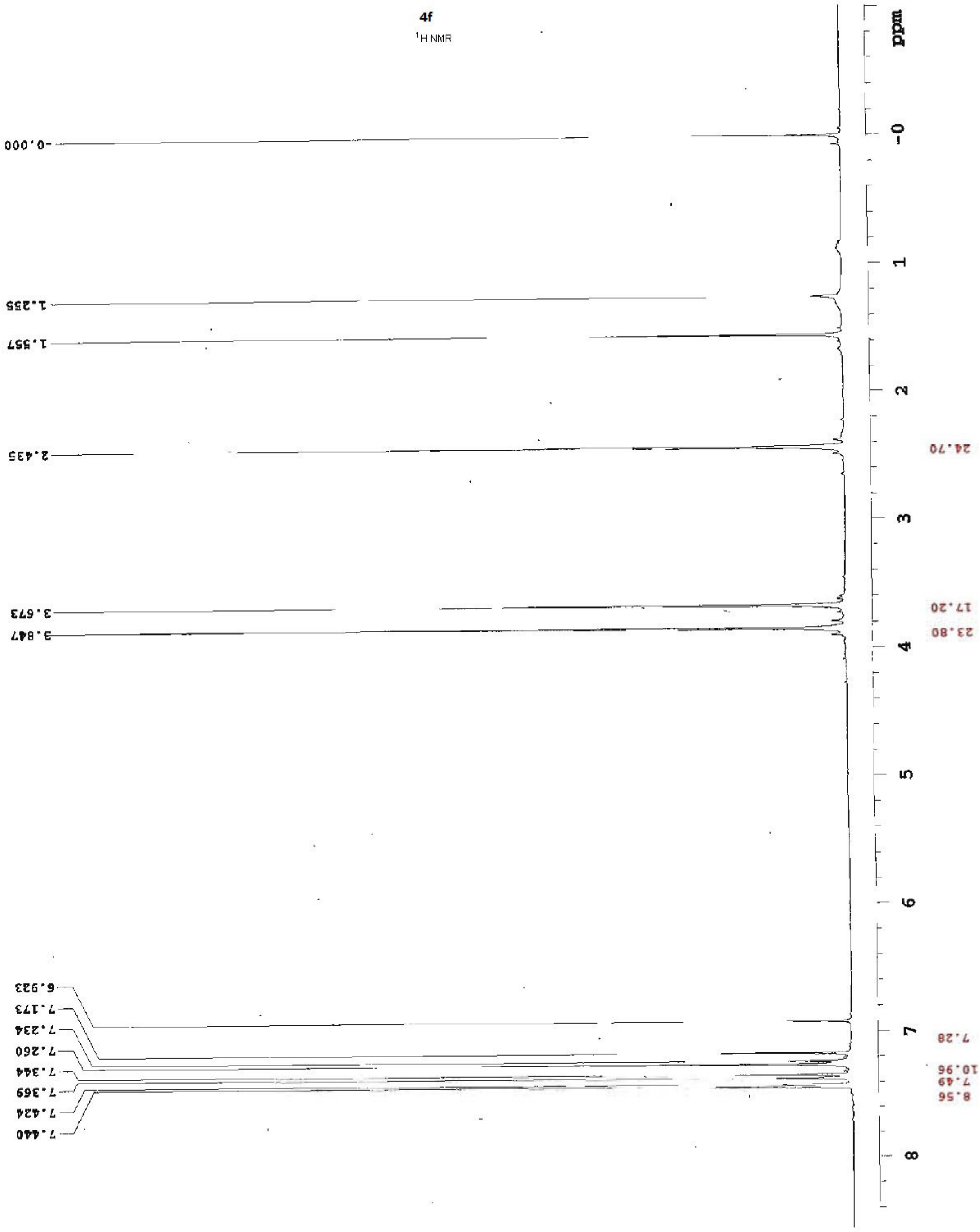
-0.000
1.255
1.557
2.435
3.673
3.847
6.923
7.173
7.234
7.260
7.344
7.369
7.424
7.440
24.70
17.20
23.80
7.28
10.96
7.49
8.56
ppm
-0
1
2
3
4
5
6
7
8

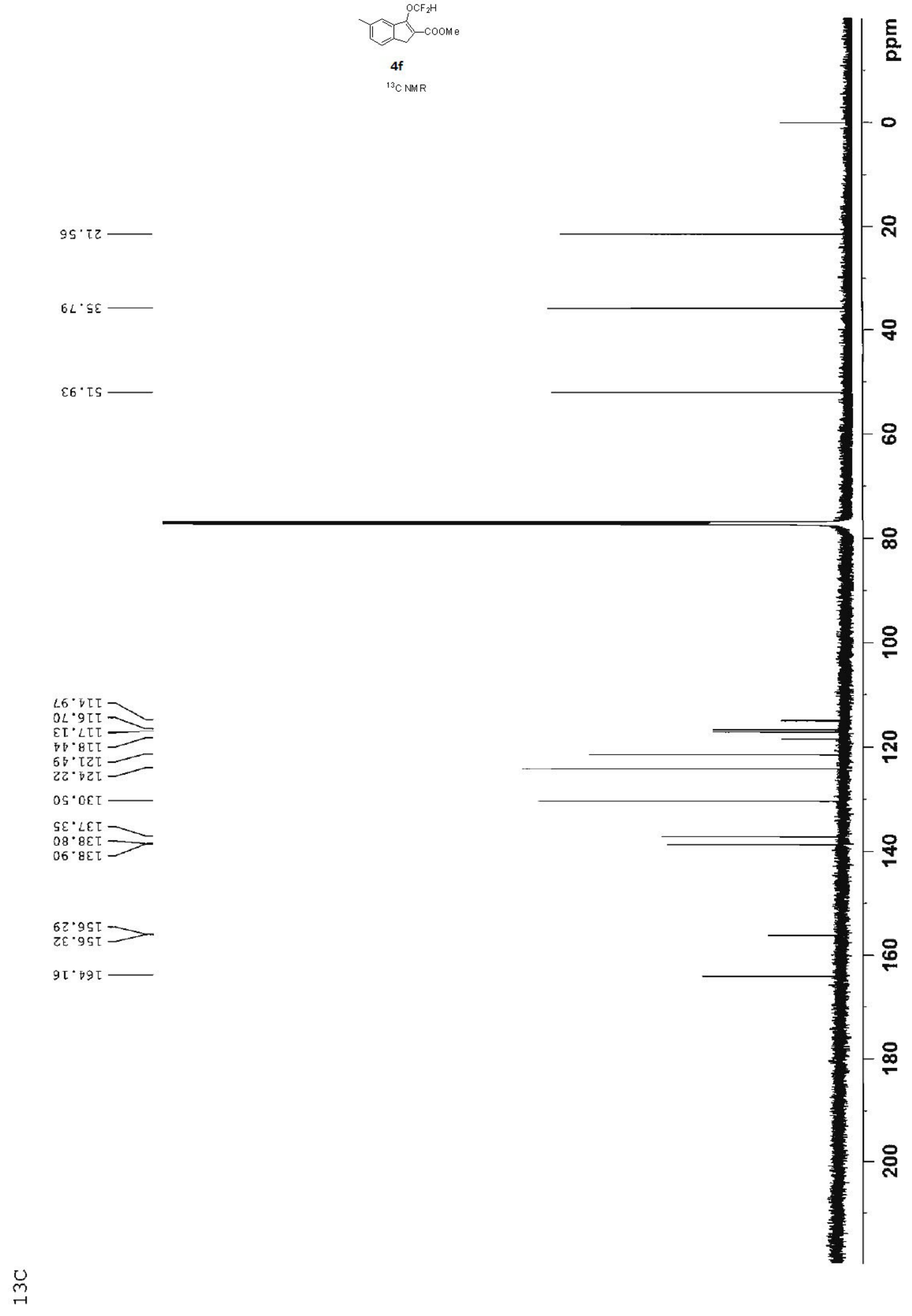
OCF2H
COOMe
4f
13C NMR
13C
21.56
35.79
51.93
114.97
116.70
117.13
118.44
121.49
124.22
130.50
137.35
138.80
138.90
156.29
156.32
164.16
ppm
0
20
40
60
80
100
120
140
160
180
200

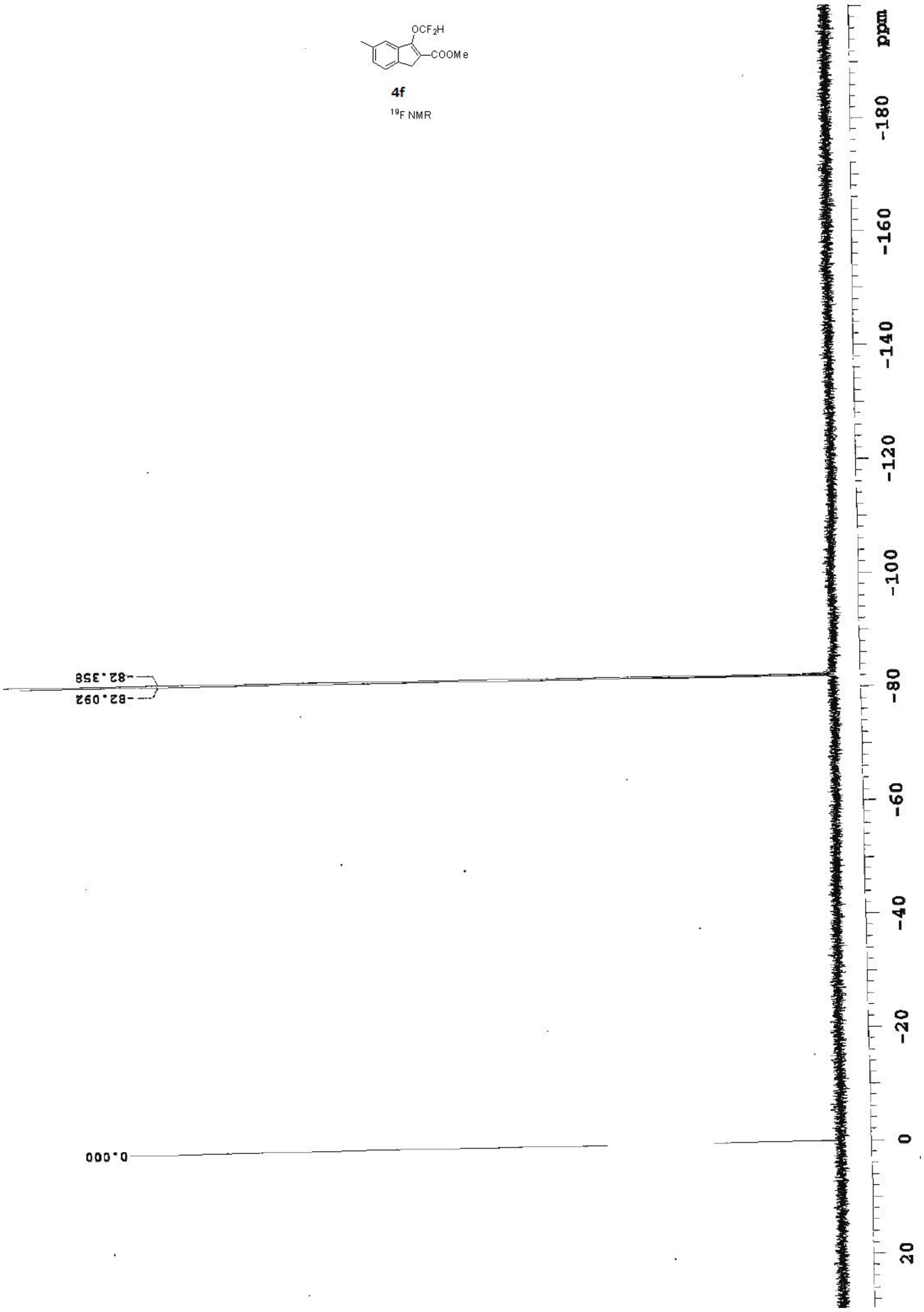
OCF$_2$H
COOMe
4f
^{19}F NMR
-82.358
-82.092
0.000
ppm
-180
-160
-140
-120
-100
-80
-60
-40
-20
0
20

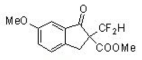

3g

^{1}H NMR

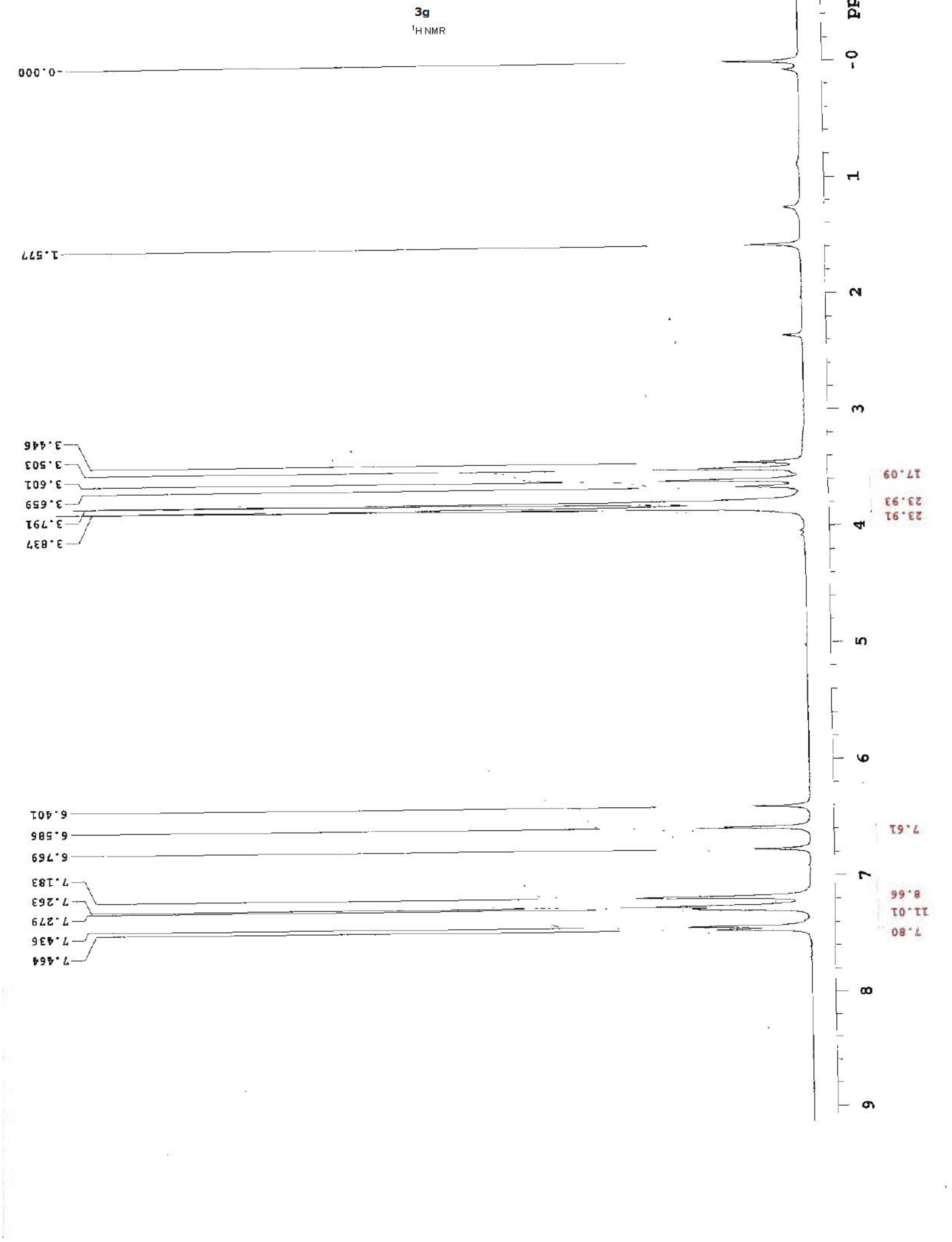

-0.000
1.577
3.446
3.503
3.601
3.659
3.791
3.837
6.401
6.586
6.769
7.183
7.263
7.279
7.436
7.464
17.09
23.93
23.91
7.61
8.66
11.01
7.80
ppm

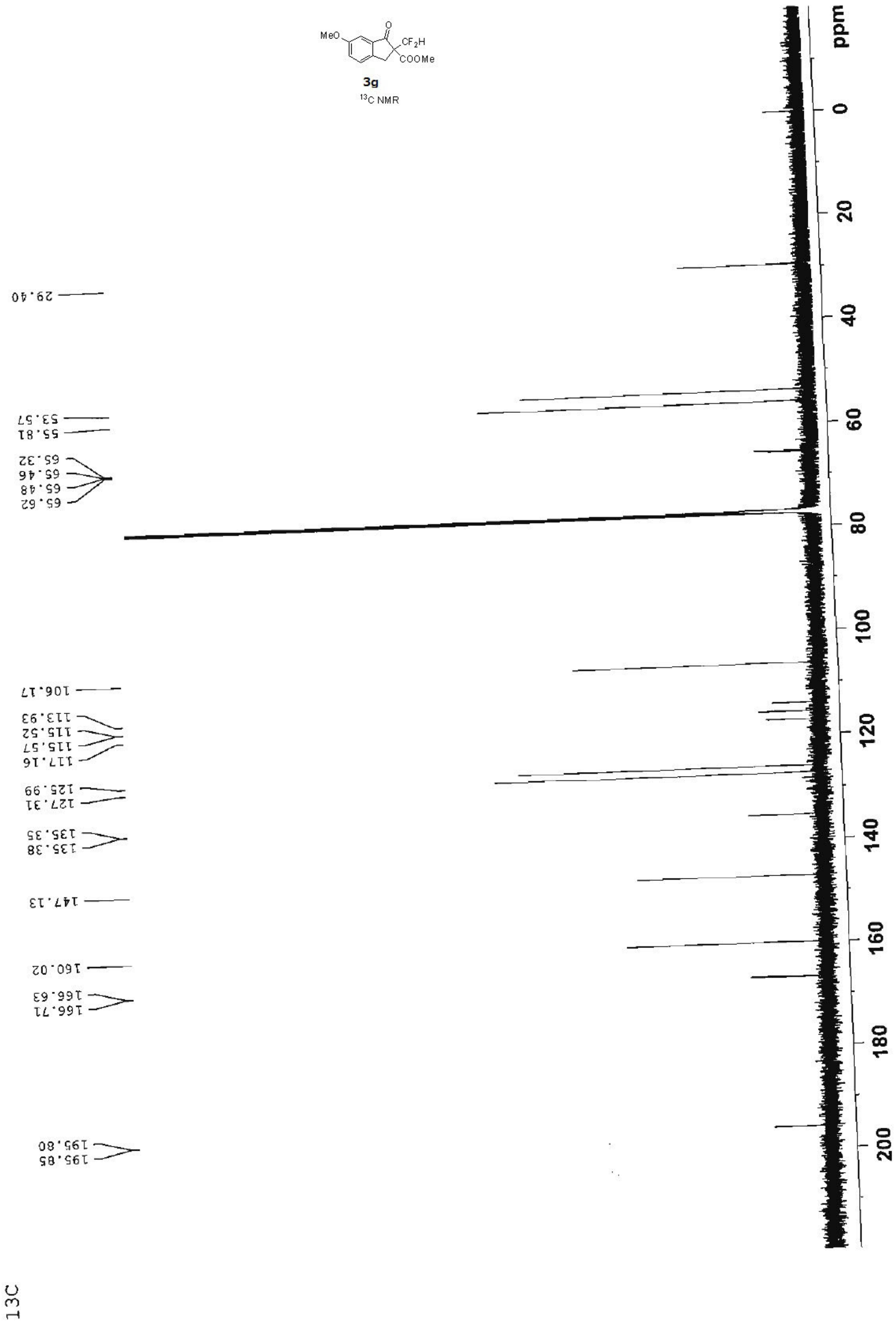
MeO
CF2H
COOMe
3g
13C NMR
29.40
53.57
55.81
65.32
65.46
65.48
65.62
106.17
113.93
115.52
115.57
117.16
125.99
127.31
135.35
135.38
147.13
160.02
166.63
166.71
195.80
195.85
13C
ppm
0
20
40
60
80
100
120
140
160
180
200

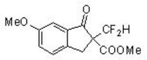
MeO
O
CF_2H
COOMe

3g

^{19}F NMR

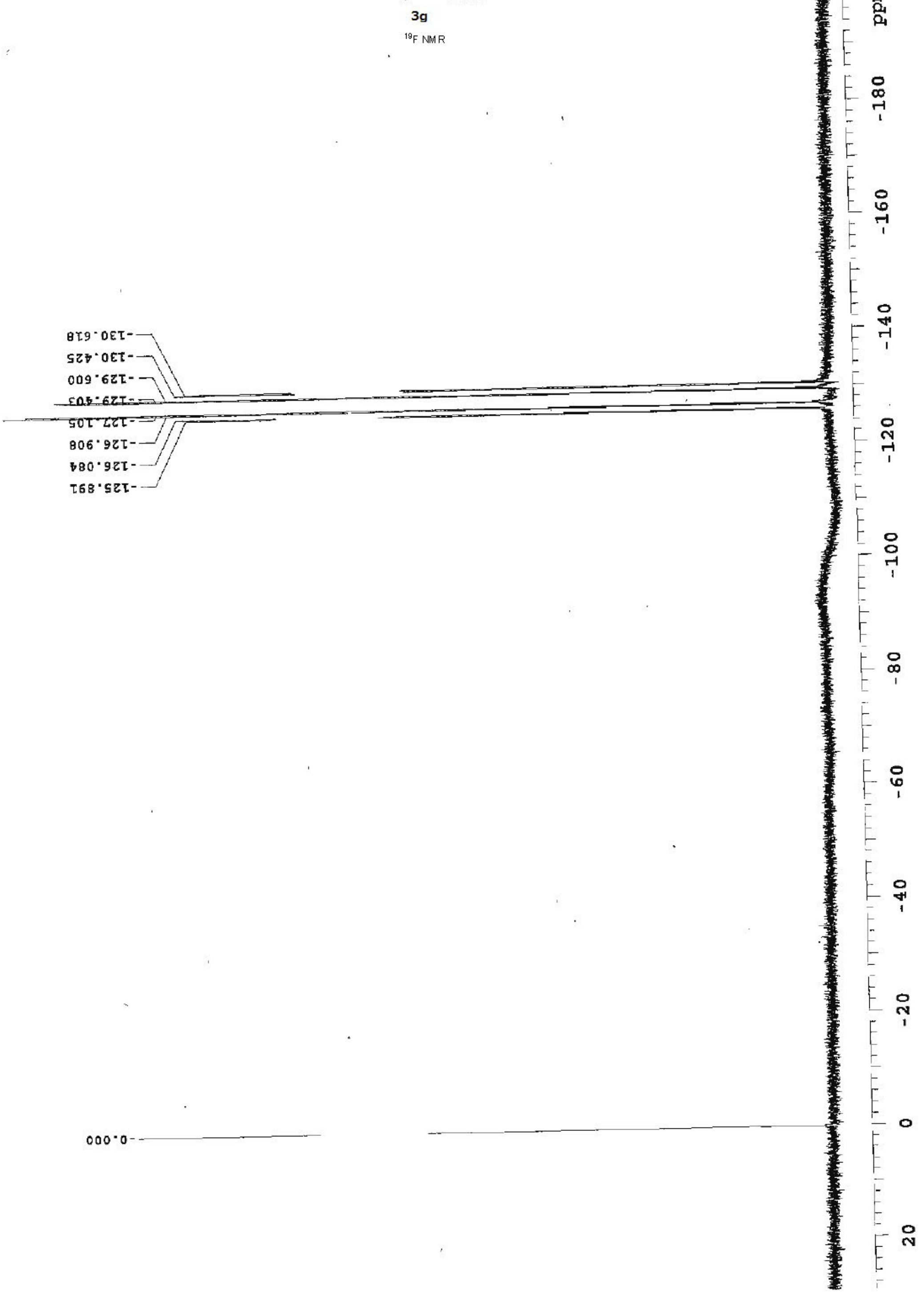
-130.618
-130.425
-129.600
-129.403
-127.105
-126.908
-126.084
-125.891
-0.000
ppm
-180
-160
-140
-120
-100
-80
-60
-40
-20
0
20

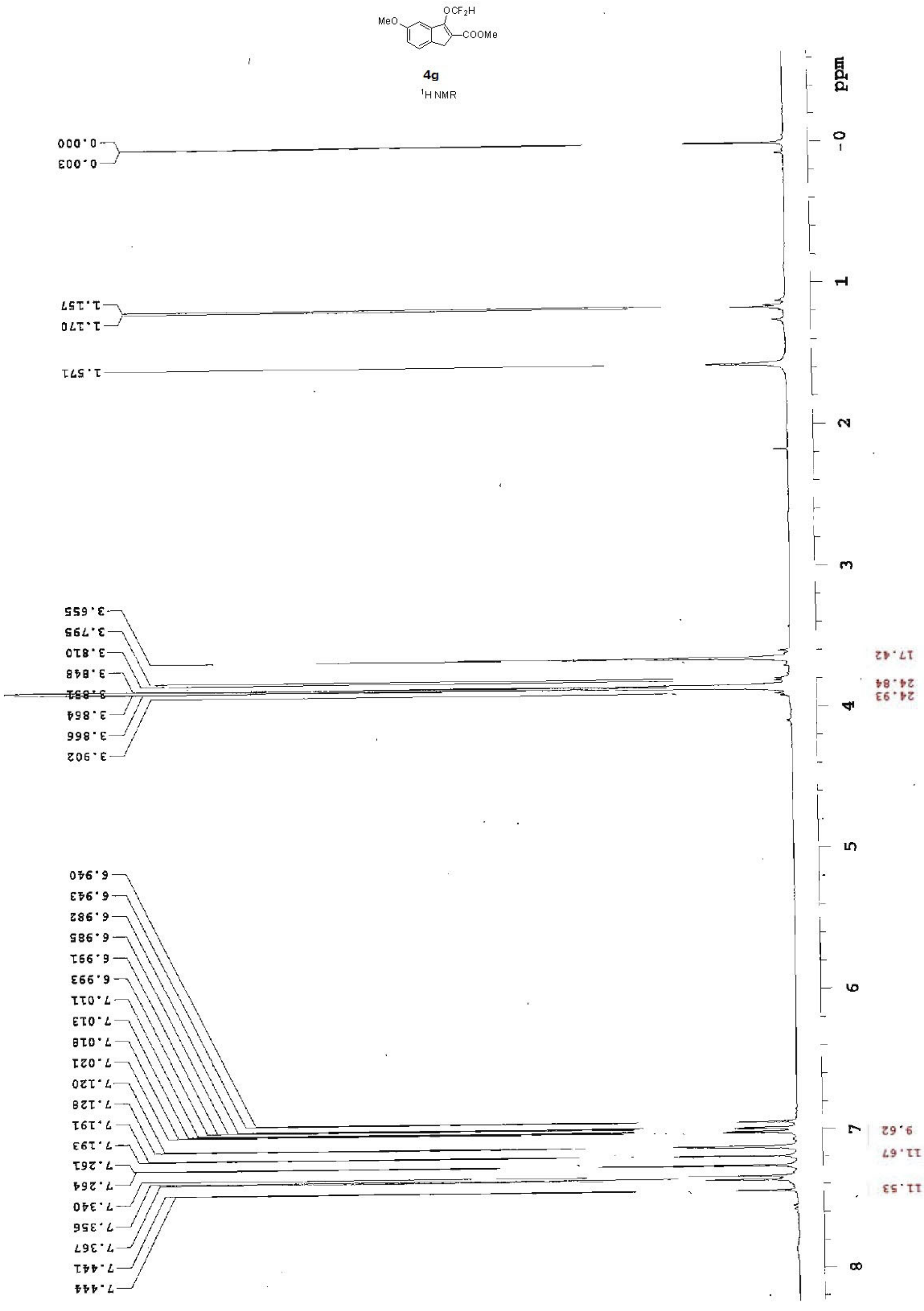
MeO
OCF2H
COOMe
4g
1H NMR
ppm
0.000
0.003
1.157
1.170
1.571
3.655
3.795
3.810
3.848
3.881
3.894
3.866
3.902
6.940
6.943
6.982
6.985
6.991
6.993
7.011
7.013
7.018
7.021
7.120
7.128
7.191
7.193
7.261
7.264
7.340
7.356
7.367
7.441
7.444
17.42
24.84
24.93
9.62
11.67
11.53

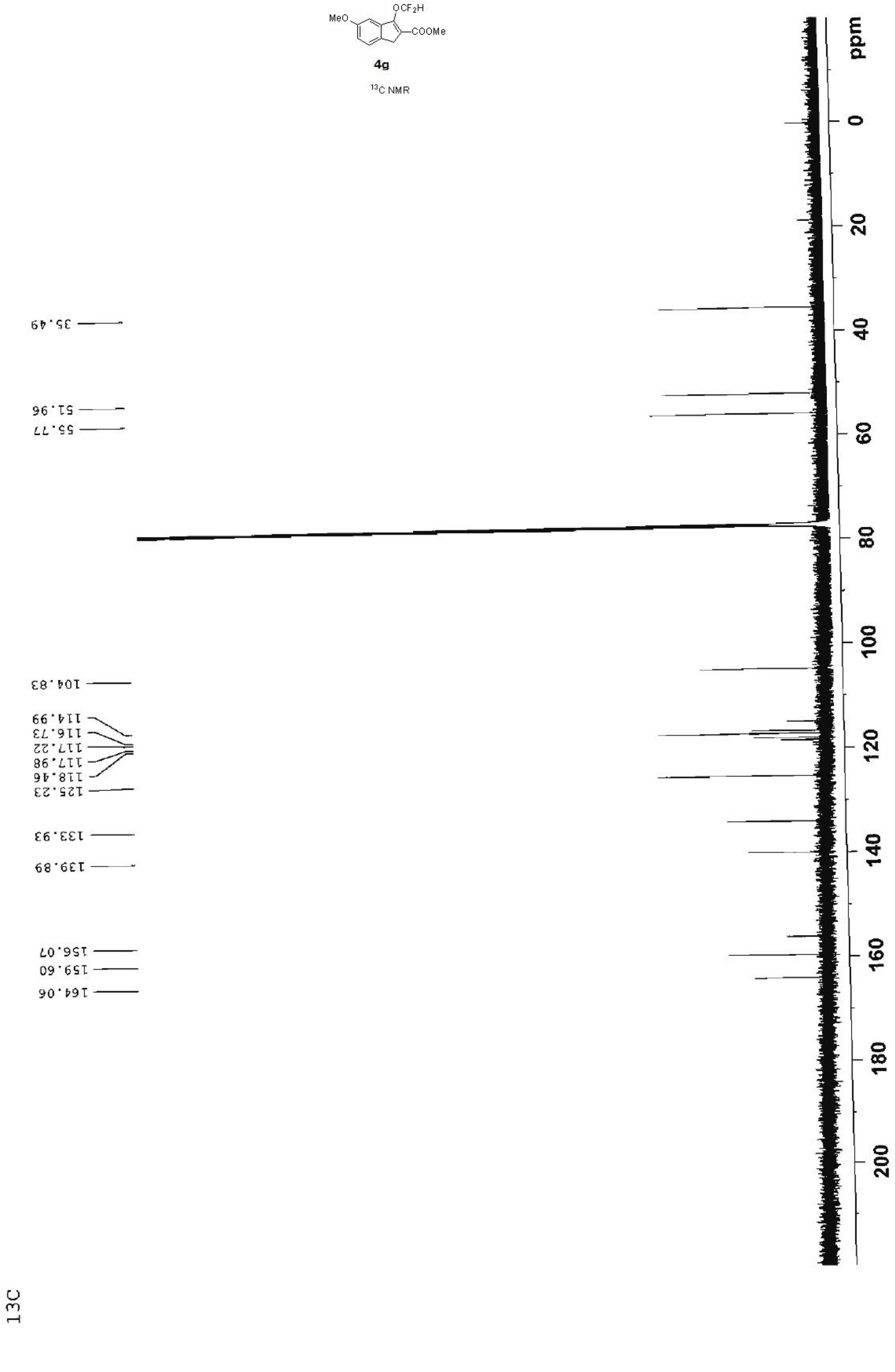
MeO
OCF2H
COOMe
4g
13C NMR
35.49
51.96
55.77
104.83
114.99
116.73
117.22
117.98
118.46
125.23
133.93
139.89
156.07
159.60
164.06
ppm
0
20
40
60
80
100
120
140
160
180
200
13C

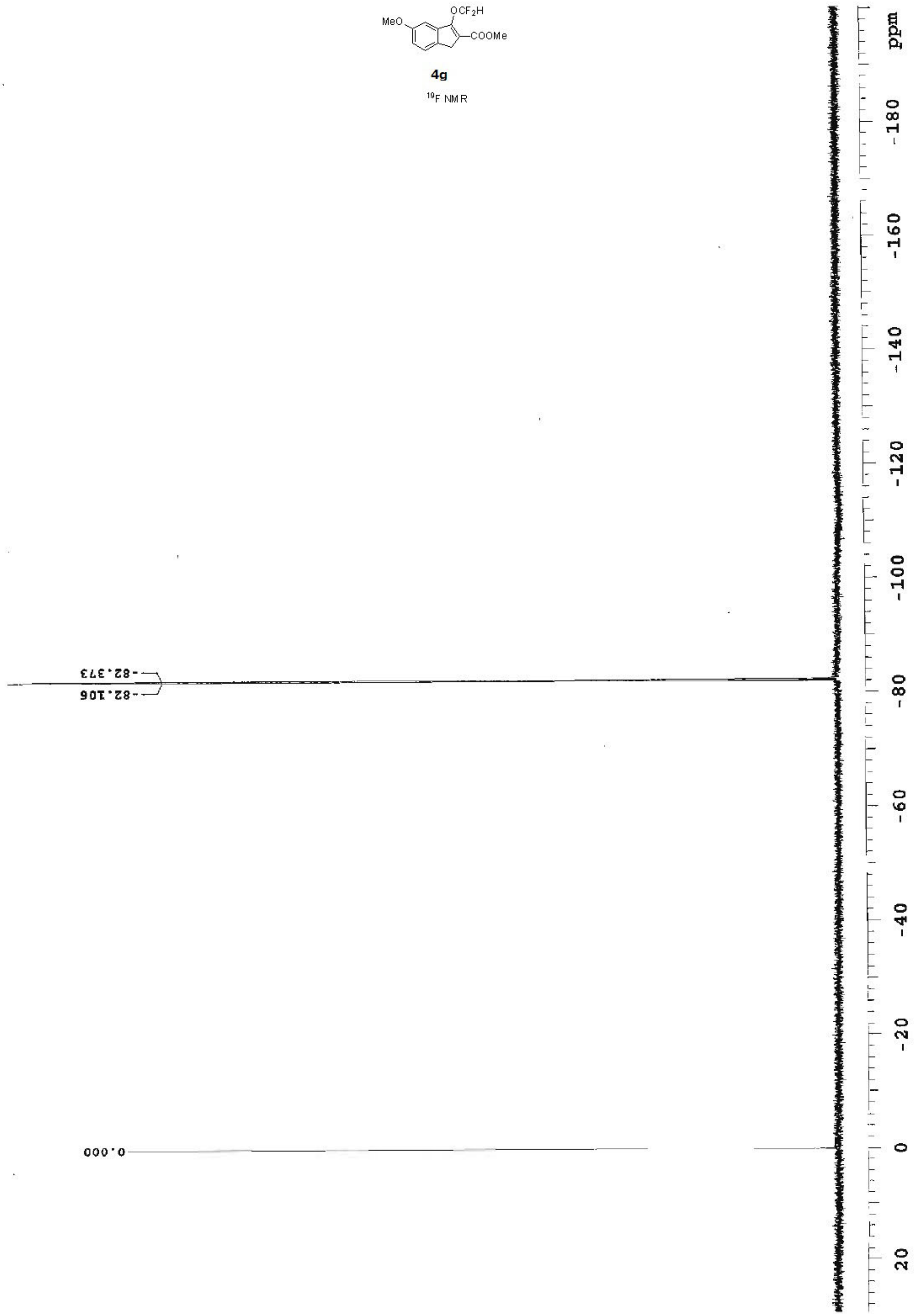
MeO
OCF$_2$H
COOMe
4g
^{19}F NMR
-82.106
-82.373
0.000
ppm
